# Black Queen Hypothesis, partial privatization, and quorum sensing evolution

**DOI:** 10.1371/journal.pone.0278449

**Published:** 2022-11-30

**Authors:** Lucas Santana Souza, Yasuhiko Irie, Shigetoshi Eda

**Affiliations:** 1 Department of Ecology & Evolutionary Biology, University of Tennessee, Knoxville, Tennessee, United States of America; 2 Department of Physics, Chemistry, and Biology, Linköping University, Linköping, Sweden; 3 Department of Forestry, Wildlife and Fisheries, University of Tennessee Institute of Agriculture, Knoxville, Tennessee, United States of America; 4 Department of Microbiology, University of Tennessee, Knoxville, Tennessee, United States of America; University of Bath, UNITED KINGDOM

## Abstract

Microorganisms produce costly cooperative goods whose benefit is partially shared with nonproducers, called ‘mixed’ goods. The Black Queen Hypothesis predicts that partial privatization has two major evolutionary implications. First, to favor strains producing several types of mixed goods over nonproducing strains. Second, to favor the maintenance of cooperative traits through different strains instead of having all cooperative traits present in a single strain (metabolic specialization). Despite the importance of quorum sensing regulation of mixed goods, it is unclear how partial privatization affects quorum sensing evolution. Here, we studied the influence of partial privatization on the evolution of quorum sensing. We developed a mathematical population genetics model of an unstructured microbial population considering four strains that differ in their ability to produce an autoinducer (quorum sensing signaling molecule) and a mixed good. Our model assumes that the production of the autoinducers and the mixed goods is constitutive and/or depends on quorum sensing. Our results suggest that, unless autoinducers are costless, partial privatization cannot favor quorum sensing. This result occurs because with costly autoinducers: (1) a strain that produces both autoinducer and goods (fully producing strain) cannot persist in the population; (2) the strain only producing the autoinducer and the strain producing mixed goods in response to the autoinducers cannot coexist, i.e., metabolic specialization cannot be favored. Together, partial privatization might have been crucial to favor a primordial form of quorum sensing—where autoinducers were thought to be a metabolic byproduct (costless)—but not the transition to nowadays costly autoinducers.

## 1 Introduction

The maintenance of microbial strains producing costly beneficial goods (cooperators) is challenging to explain because nonproducing strains (cheaters) are expected to outcompete producing strains [1, 2] by taking advantage of the benefits of goods without paying the cost of producing goods. Growing evidence shows that benefits are not always equally shared among producers and nonproducers [3–5]. These goods, providing privatized and public benefits, have been called “mixed” goods [6]. Morris et al. proposed the Black Queen Hypothesis which predicts that the growth of mixed good producers is favored over that of nonproducers whenever the privatized benefits offset the costs of producing goods [3–5, 7].

The production of mixed goods is regulated by the population density-dependent [8] (and/or frequency-dependent [9]) gene regulation of bacteria, called quorum sensing (QS) and/or a QS-independent mechanism. One example of mixed goods is pyoverdine, an iron-scavenging siderophores of *Pseudomonas aeruginosa* [7, 10, 11]. The benefit of siderophore is partially privatized as only a proportion of the molecules is secreted from the bacteria [7, 10]. The partially secreted siderophore provides benefits to nonproducing strains. Another mechanism of partial privatization includes the intracellular cleavage of sucrose into monosaccharides. Some monosaccharides remain within the cell, i.e., they are privatized, and others leak into the extracellular environment [3].

The Black Queen Hypothesis predicts that cooperation can be maintained in two main ways: by favoring a strain producing all cooperative goods (a fully producing strain) [12–14] or by favoring different complementary strains, each producing a cooperative good (i.e., metabolic specialization) [12, 13]. These two predictions were also found in models that involved constitutive (QS-independent) production of goods. However, despite evidence that QS regulates mixed goods [7, 10], no model analyzed whether these two predictions are valid for QS evolution.

There are two reasons for why it is unclear whether partially privatized benefits can favor QS via a fully producing strain which produces both QS signaling molecules (autoinducers) and goods. First, while QS-independent regulation of mixed goods might be costless, QS-regulation itself is costly. Thus, if a mixed good is regulated by a costless, QS-independent mechanism (e.g., constitutively produced), then benefits only need to offset the mixed goods’ costs [3]. However, when mixed goods are QS regulated, privatized benefits need to offset not only the costs of mixed goods but also autoinducers’ cost. Second, experiments that involve QS regulation of a mixed good have successfully shown that the partially privatized benefits suppress the invasion of strains that are not producing mixed goods [15]. The problem is that these studies focus on the interactions between two strains, which might not reflect the case when more than two strains are present [16]. Thus, it is not clear whether partial privatization supports the growth of a fully producing strain.

Additionally, whether partial privatization favors QS via metabolic specialization (i.e., via one strain only producing autoinducers and the other only responding to it) is unclear. That is because metabolic specialization has only been demonstrated by mathematical models assuming a costless regulation of two mixed goods [12, 13, 17]. The problem is that while a QS-independent regulation of two mixed goods might be costless, QS-regulation requires the production of autoinducers, which are costly [18–21]. However, if autoinducers were costless (a metabolic byproduct), as it is thought to be in its origins [22–26], then partial privatization might have fostered a primordial form of QS. Despite metabolic specialization being a prediction of the Black Queen Hypothesis [12, 14], whether QS can be maintained through metabolic specialization have not been tested.

Here, we built the first mathematical model to examine whether QS can be maintained in the population due to the partial privatization of benefits from a mixed good. Our model evaluated whether QS is favored by either the maintenance in the population of (A) a strain that produces both autoinducers and mixed goods (fully producing strain); or (B) through the coexistence of two strains, one only producing autoinducers and the other only producing mixed goods (metabolic specialization). Our results suggest that partial privatization cannot provide a complete explanation for quorum sensing evolution because partial privatization could have favored the evolution of QS systems based on costless autoinducers but not costly ones.

## 2 Results

In our model, we assumed that two types of costly molecules are produced, namely an autoinducer (QS signaling molecule) and a mixed good. We considered four strains, each carrying one of two different alleles in two gene loci (Table 1). At locus ***A***, allele *A* produces an autoinducer that diffuses throughout the environment. Allele *a* is incapable of producing the autoinducer. At locus ***G***, allele *G* recognizes the autoinducers and produces a mixed good through a QS-independent (constitutive production) and QS-dependent (i.e., in response to autoinducers frequency) mechanisms. Allele *g* cannot recognize the autoinducers and cannot produce the mixed good.

**Table 1:** Strain types and a description of their allele functions.

STRAIN	ALLELE FUNCTION
*AG*	*Autoinducer-producer/Good-producer*
*aG*	*Autoinducer-nonproducer/Good-producer*
*Ag*	*Autoinducer-producer/Good-nonproducer*
*ag*	*Autoinducer-nonproducer/Good-nonproducer*

In nature, mixed goods can provide benefits by reducing cell death [5, 7], or promoting cell growth [3]. Here, we assume that the private and public benefits of the mixed good promote growth. The production of the mixed goods and the autoinducers might occur solely by QS-independent mechanism or together with QS-dependent mechanism. The four strains considered in this work are summarized in the Table 1. The strain *AG* regulates the production of the autoinducer and the mixed good by QS-independent and QS-dependent mechanisms. The strain *aG* cannot produce the autoinducer but produces the mixed good by QS-independent and QS-dependent regulation. The strain *Ag* produces the autoinducer through a QS-independent mechanism but not the mixed good. The strain *ag* neither produces autoinducer nor the mixed good. For simplicity, hereafter, mixed goods will be just referred as goods.

Here, we analyzed the evolution of the population genetics by tracking each strain’s frequency. To evaluate whether partially privatized benefits favor QS, we first checked whether the strain *AG* could be maintained when in pairwise interaction with *aG*, *Ag*, or *ag*. Since *AG* can produce and recognize the autoinducer, we consider QS is maintained when *AG* is present in the population. QS could also be maintained by the coexistence between *Ag* and *aG*, we tested whether selection favors their coexistence (favor metabolic specialization). Lastly, we tested whether partially privatized benefits can favor QS, the maintenance of alleles *A* and *G*, when all strains are simultaneously present in the population. For a detailed description of the model, see the model section. Parameters, variables, and functions are detailed on Table 2.

**Table 2:** List of parameters and variables and their meaning and range.

**Parameter**	**Meaning**	**Range**
*e*	Proportion of benefits privatized	0 ≤ *e* ≤ 1
*C*_*G*_	Per capita cost of producing goods	0 ≤ *C*_*G*_ < 0.5
*C*_*A*_	Per capita cost of producing autoinducers	0 ≤ *C*_*A*_ < 0.5
*L*	Proportion of autoinducers secreted through a QS-independent mechanism	0 ≤ *L* ≤ 1
*q*	Rate of production in response to autoinducers (QS-dependent regulation)	0 ≤ *q* ≤ 0.5
*q*_0_	Rate of constitutive production (QS-independent regulation)	0 ≤ *q*_0_ ≤ 0.5
**Variable**	**Meaning**	**Range**
*A*_*t*_	Frequency of autoinducers at time *t*	0 ≤ *A*_*t*_ ≤ 1
*G*_*t*_	Frequency of goods at time *t*	0 ≤ *G*_*t*_ ≤ 1
*x*_*ij*,*t*_	Frequency of strain *ij* at time *t*	0 ≤ *x*_*ij*_,_*t*_ ≤ 1

### (A) Pairwise analysis of *AG* and *ag*

**Fig 1:**
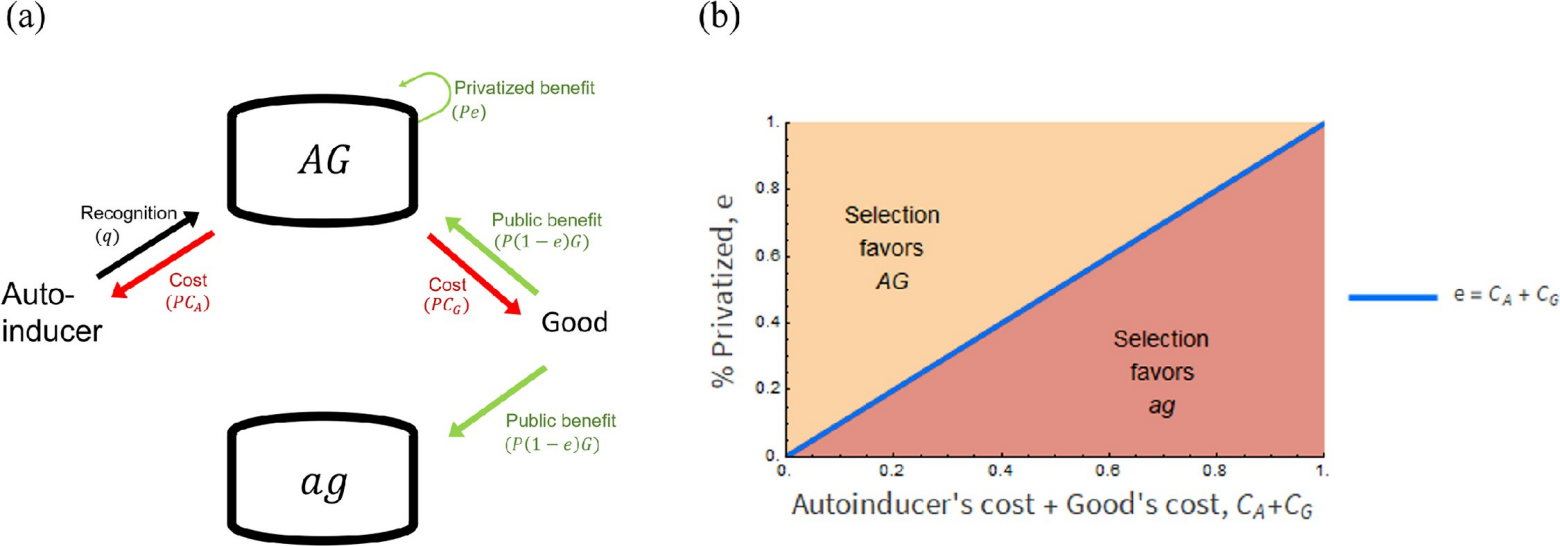
The partially privatized benefit enables *AG* to outcompete *ag*. **(a)** A schematic of the social interaction between strains *AG* and *ag*. Red arrows indicate a costly production of a good and an autoinducer. The black arrow indicates that the autoinducer triggers the production of functional alleles (*A* and *G*). Green arrows indicate the access to the good’s benefits. *AG* accesses both public and private benefits, while *ag* accesses only public benefit. **(b)** A geometric visualization of which strain selection favors. In yellow and red areas, selection favors pure populations of *AG* and *ag*, respectively. The blue line is where both strains are equally fit, which happens when the per capita partially privatized benefit equalizes to the sum of the per capita costs of producing an autoinducer and a good. This result is valid for parameters: 0 ≤ *L* ≤ 1 and having either *q* ≠ 0, or *q*_0_ ≠ 0 or 0 < *q*_0_, *q* ≤ 0.5. Please see Table 2 and Section 6A for the definition of letters (*q*, *C*_*A*_ etc.) and the equations used for the analysis, respectively. All mathematical analyses assumed an unstructured population.

As depicted in Fig 1A, *ag* exploits *AG* by neither producing the autoinducer nor the good. Here, we analyzed whether QS can be maintained in a population by checking if selection favors pure populations of *AG* or the coexistence of *AG* and *ag*.

We found that—at any initial ratio of both strains—selection favors pure populations of *AG* if the per capita partially privatized benefit offsets the per capita cost of producing both the good and the autoinducer. Otherwise, if the per capita partially privatized benefit cannot offset the per capita cost of good and autoinducer production, selection favors pure populations of *ag* (Fig 1B). This selection outcome occurs because while both strains equally access the shared benefit, only *AG* accesses privatized benefits and pays the production costs. In sum, the factor that determines the outcome of selection is the relative amount of privatized benefit to cost—not the absolute amount of privatized benefit (Section 6A).

Moreover, we found that the ability to produce autoinducers and goods in response to autoinducers neither favors nor disfavors any of the two strains (Eq 5). This occurs because while *AG* accesses the net difference between the privatized benefits and costs for both QS-independent and -dependent mechanisms, *ag* does not access this net difference at all. Similarly, the ability to produce autoinducers and goods by a QS-independent mechanism (constitutively) neither favors nor disfavors any of the two strains. The type of regulation, QS-dependent or QS-independent does not matter because only *AG* produces autoinducers and goods.

### (B) Pairwise analysis of *AG* and *Ag*

**Fig 2:**
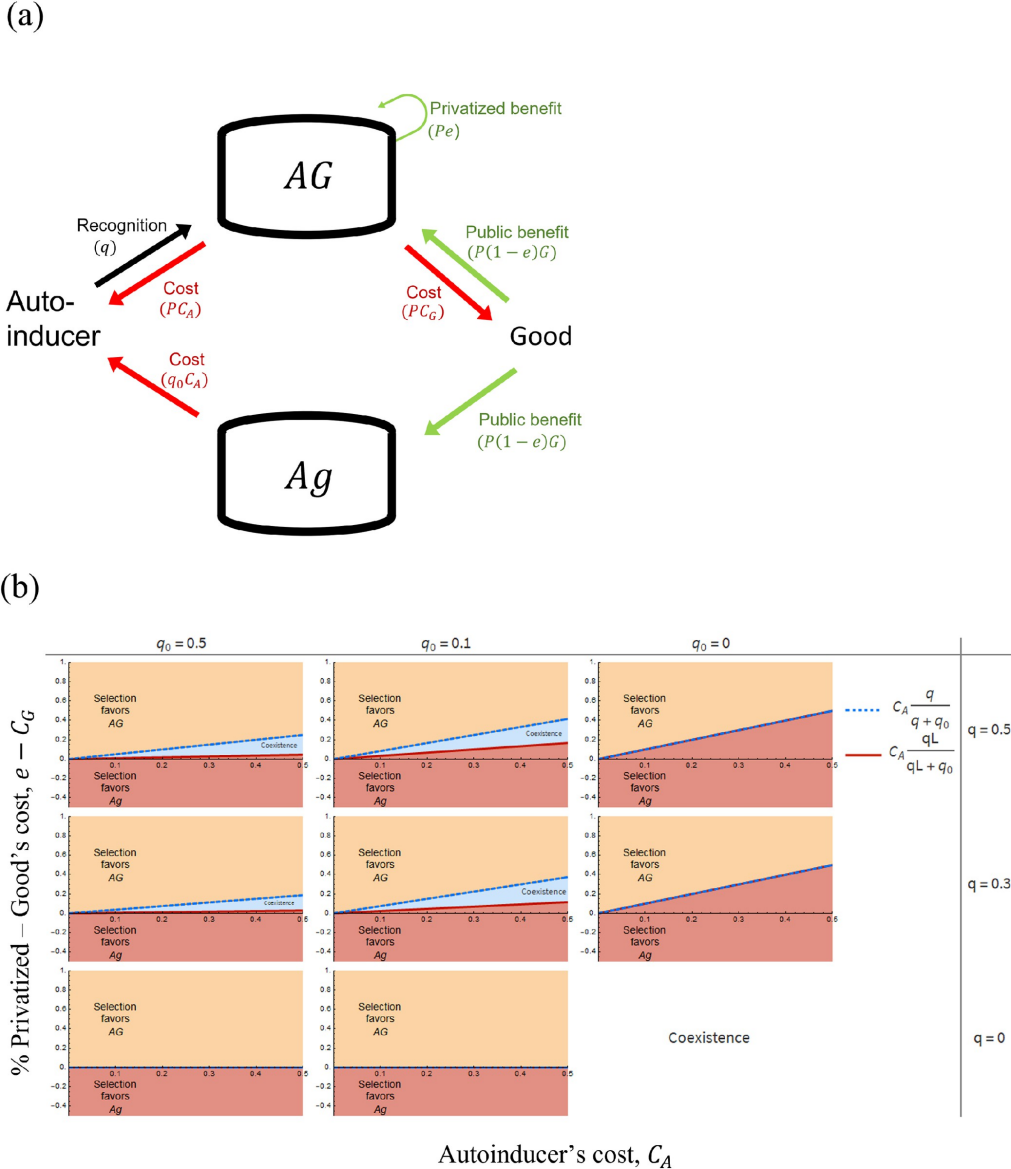
The partially privatized benefit favors QS through pure populations of *AG* or through mixed populations of *AG* and *Ag*. **(a)** A schematic social interaction between strains *AG* and *Ag*. **(b)** Selection either favors (i) pure populations of *AG* (yellow area); (ii) pure populations of *Ag* (red area); (iii) coexistence of *AG* and *Ag* (blue area). Selection favors the coexistence if the rare strain outcompetes the common one (i.e., negative frequency-dependent selection). The frequency of each strain within the blue is not uniform (S1 Fig). Negative frequency-dependent selection emerges from the co-regulation of QS (*q*) and QS-independent (*q*_0_) mechanisms, the existence of privatization (*e*) and having privatized benefits offsetting the minimum autoinducer’s cost, $C_{A}\frac{qL}{q_{0}+qL}$ (which occurs when the population is solely composed by *Ag* such that *A*_*t*_ = *L*), but not the maximum autoinducer’s cost, $C_{A}\frac{q}{q_{0}+q}$ (which occurs when the population is solely composed by *AG* such that *A*_*t*_ = 1). The x-axis is the per capita autoinducer’s cost, *C*_*A*_. The y-axis is the difference between the per capita partially privatized benefit and the per capita good’s cost, *e* − *C*_*G*_. Each subgraph captures the effect of regulatory architecture, via QS-dependent and QS-independent mechanisms. *q* = 0 implies absence of QS regulation. *q*_0_ = 0 implies absence of QS-independent regulation. Parameter: *L* = 0.1. Please see Table 2 and Section 6B for the definition of letters (*q*, *C*_*A*_ etc.) and the equations used for the analysis, respectively. All mathematical analyses assumed an unstructured population.

As depicted in Fig 2A, *Ag* exploits *AG* by not producing the good and by producing fewer autoinducers. Here, we analyzed whether QS can be maintained in a population by checking if selection favors pure populations of *AG* or the coexistence of *AG* and *Ag*.

At any initial ratio of both strains, we found that selection can favor three outcomes. First, *AG* always outcompetes *Ag* if the difference between the total partially privatized benefit and the total good’s cost offsets the maximum autoinducer’s cost (yellow area in Fig 2B). The maximum autoinducer cost that *AG* pays and *Ag* does not, *C*_*A*_*q*, occurs when the autoinducer frequency is at its maximum value, which occurs when the population is solely composed of *AG* individuals.

Second, *Ag* always outcompetes *AG* if the difference between the total partially privatized benefit and the total good’s costs cannot offset the minimum autoinducer’s cost (red area in Fig 2B). The minimum autoinducer cost that *AG* pays and *Ag* does not, *C*_*A*_*qL*, occurs when the autoinducer frequency is at its minimum value, which occurs when the population is solely composed of *Ag* individuals, such that all autoinducers are only produced constitutively.

Lastly, selection favors the coexistence of *AG* and *Ag* if the difference between the total partially privatized benefit and total good’s cost offsets the minimum autoinducer’s cost but not the maximum autoinducer’s cost. In this case, selection favors the rare strain over the common one (blue area in Fig 2B), i.e., negative frequency-dependent selection [27].

Moreover, we found that the ability to produce good in response to autoinducers, *q*, favors *Ag* over *AG*. This is because while both strains pay for autoinducer costs from QS-independent regulation, only *AG* pays for autoinducer costs from QS-dependent regulation (Section 6B). This is graphically noticeable in Fig 2B: the area where *Ag* outcompeted *AG*, red area, increases as the relative contribution of QS regulation increases (as *q* increases).

### (C) Pairwise analysis of *AG* and *aG*

**Fig 3:**
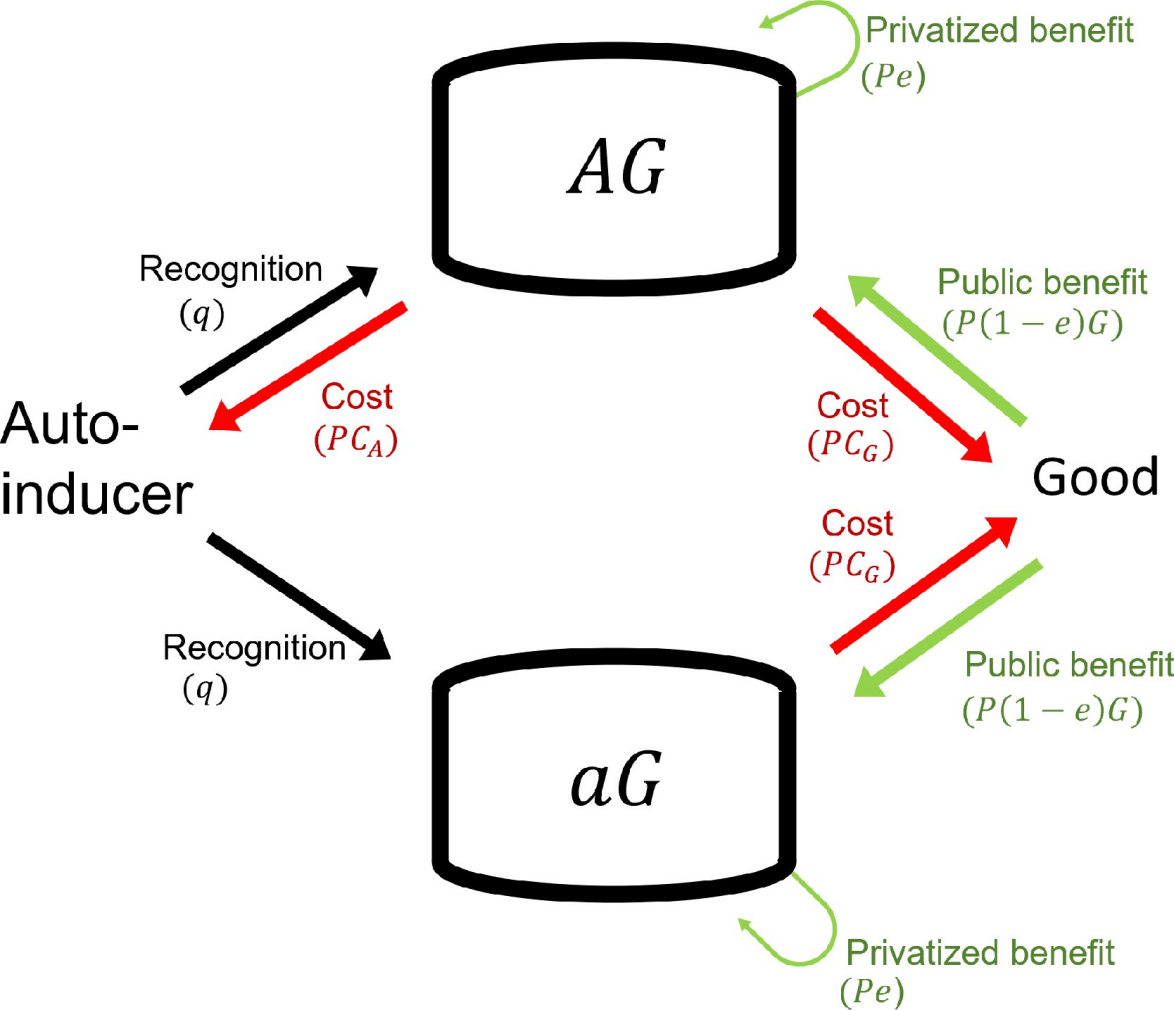
Partial privatization is ineffective against *aG* strains. Selection favors *aG* over *AG* because both strains access the private and public benefits and pay the good’s cost, but only *AG* pays the autoinducer’s cost. *aG* can use autoinducers to trigger the production of goods. *AG* can use autoinducers to trigger the production of more autoinducers and goods. Please see Table 2 and Section 6C for the definition of letters (*q*, *C*_*A*_ etc.) and the equations used for the analysis, respectively. All mathematical analyses assumed an unstructured population.

As depicted in Fig 3, *aG* exploits *AG* by not producing autoinducers while use them to trigger the production of the good. Here, we analyzed whether QS can be maintained in a population by checking if selection favors pure populations of *AG* or the coexistence of *AG* and *ag*.

At any initial ratio of *AG* and *aG*, we found that unless autoinducers are costless, selection always favors *aG* over *AG* (Eq 7). This is because both strains have the same access to benefits—including the partially privatized one—but only *AG* pays the autoinducer’s cost. Consequently, independently of the privatized benefit offsetting, or not, the goods’ cost, *aG* is always expected to outcompete *AG* in unstructured populations. This implies that the lower the per capita autoinducer’s cost, the more time is required for *aG* to eliminate *AG*.

### (D) Pairwise analysis of *Ag* and *aG*

**Fig 4:**
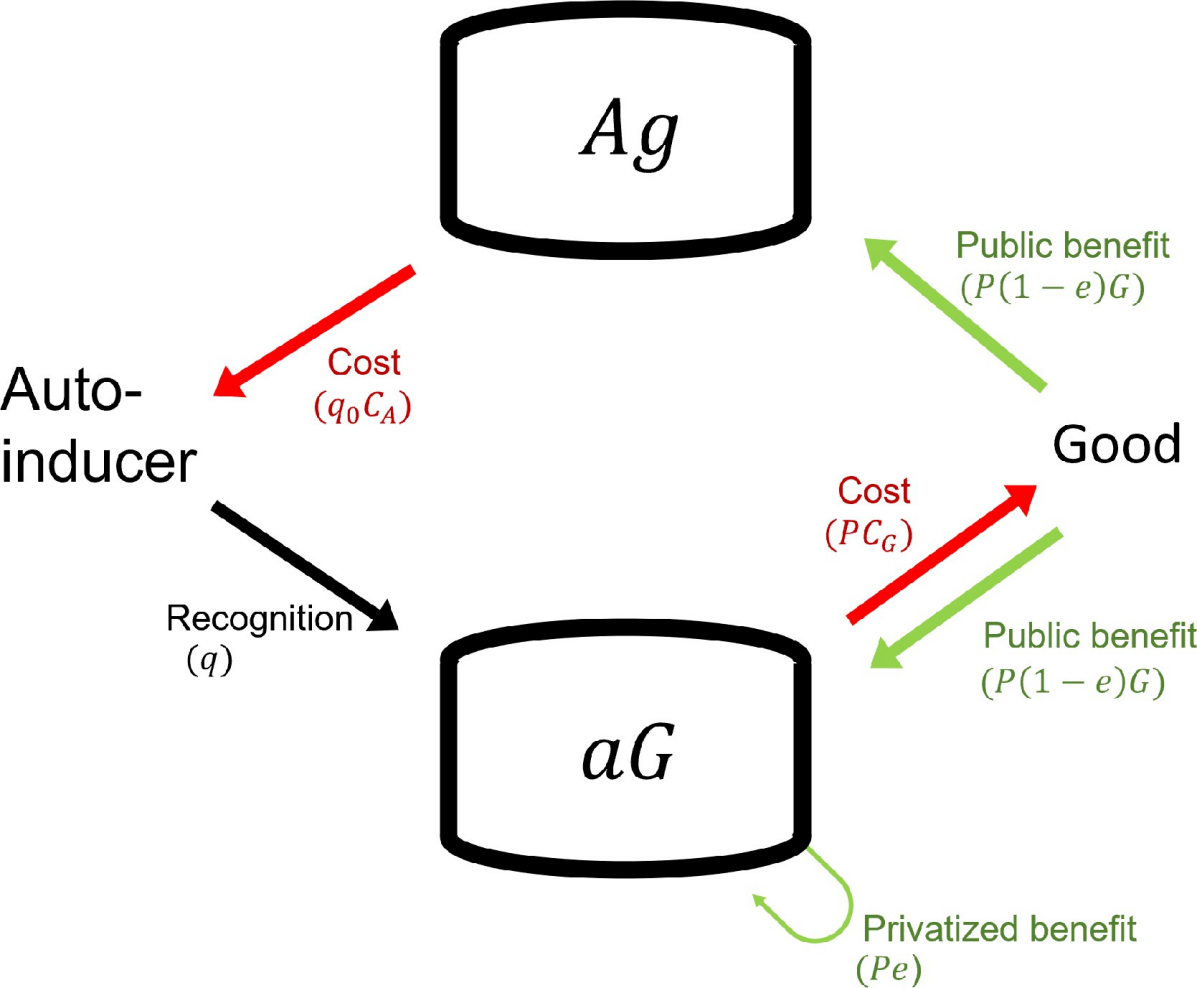
A schematic relationship for the social interaction between functionally complementary strains, i.e., the autoinducer-producer/good-nonproducer (*Ag*) and the autoinducer-nonproducer/good-producer (*aG*). Please see Table 2 and Section 6D for the definition of letters (*q*, *C*_*A*_ etc.) and the equations used for the analysis, respectively. All mathematical analyses assumed an unstructured population.

As depicted in Fig 4, *Ag* exploits *aG* by not producing goods and yet accessing its public benefit. *aG* exploits *Ag* by not producing autoinducers and yet using them to trigger the production of goods. Hence, both strains have aligned interests in accessing each other’s molecules. Here, we analyzed whether QS can be maintained in a population by checking if selection favors the coexistence of *Ag* and *aG*.

**Fig 5:**
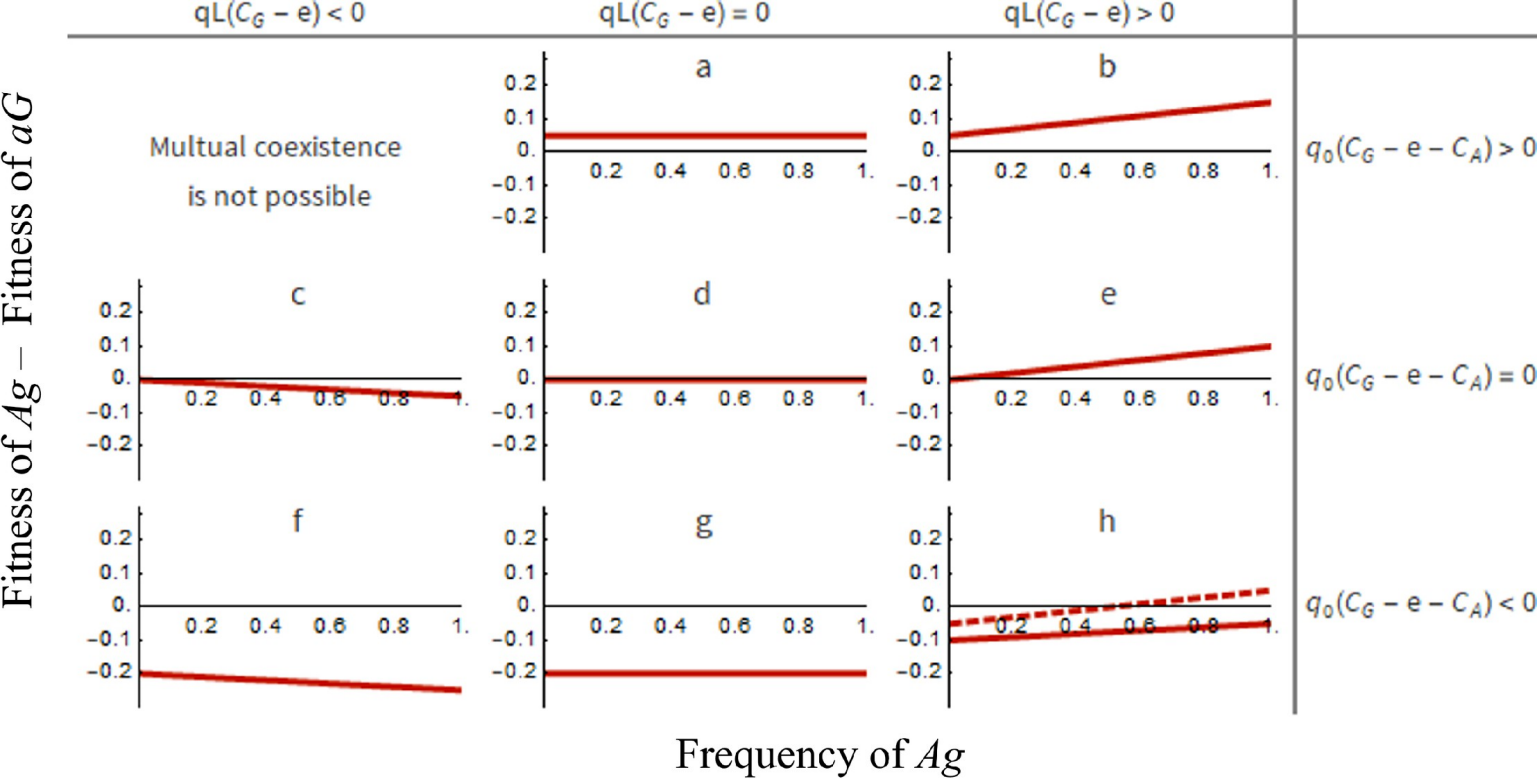
Partial privatization cannot favor coexistence of functionally complementary strains. The interaction between *Ag* and *aG* will always lead to pure populations of one of them. If the line is entirely below the x-axis, then *aG* always outcompetes *Ag*. If the line is entirely above the x-axis, then *Ag* always outcompetes *aG*. The point of intersection between the line and the x-axis indicates that both strains are equally fit. Selection cannot favor coexistence because the point of intersection, dotted line, only exists when the most common strain has the advantage over the rare strain (hence we have positive frequency-dependent selection, not negative frequency-dependent selection). *qL*(*C*_*G*_ − *e*) is the inclination of the line and represents the metabolic balance from QS regulation. *q*_0_(*C*_*G*_−*e*−*C*_*A*_) is the point of intersection with the y-axis and represents the metabolic balance from QS-independent regulation. Regarding the effect of QS regulation (*q*): *qL*(*C*_*G*_−*e*) < 0 indicates that the per capita partially privatized benefit offsets the per capita good’s cost; *qL*(*C*_*G*_−*e*) = 0 indicates a lack of QS regulation or a lack of QS-independent autoinducer secretion; *qL*(*C*_*G*_−*e*) > 0 indicates that the per capita partially privatized benefit cannot offset the per capita good’s cost. Regarding the effect of QS-independent regulation (*q*_0_): *q*_0_ (*C*_*G*_−*e*−*C*_*A*_) > 0 indicates that the per capita good’s cost is higher than the sum of the per capita partially privatized benefit and the per capita autoinducer’s cost; *q*_0_ (*C*_*G*_−*e*−*CA*) = 0 indicates a lack of QS-independent regulation; *q*_0_ (*C*_*G*_−*e*−*C*_*A*_) < 0 indicates that the per capita good’s cost is lower than the sum of the per capita partially privatized benefit and the per capita autoinducer’s cost. All mathematical analyses assumed an unstructured population.

We found that selection cannot favor coexistence of *Ag* and *aG* through negative frequency-dependent selection. Thus, partial privatization cannot favor metabolic specialization, i.e., one strain produces good only and the other produces autoinducer only (Fig 5).

*Ag* and *aG* cannot coexist via negative frequency-dependent selection because while *Ag* only outcompetes *aG* if the good’s cost is higher than the privatized benefit and the autoinducer’s cost, *C*_*G*_−*e* > *C*_*A*_, *aG* only outcompetes *Ag* if the opposite occurs, *C*_*G*_−*e* < *C*_*A*_. Hence, the alignment of interests in accessing each other’s metabolites cannot outweigh the incompatibility risen from their competition. The coexistence between both strains is only possible in a very specific scenario (Fig 5D), where the privatized benefit is exactly the same as the cost of producing goods (*C*_*G*_ = *e*), and either autoinducers are costless (*C*_*A*_ = 0) or QS is the sole mechanism regulating goods production (*q*_0_ = 0 and *q* ≠ 0). Nevertheless, these conditions listed might be biologically implausible as changes in the genetic architecture can affect the cost of producing goods [5], and tiny fluctuations in environmental condition might lead to changes on the per-capita privatized benefit, leading to privatized benefits being different than the costs [7]. Another equally unlikely biological condition with selection favoring the coexistence between *Ag* and *aG* would require the cost of goods production to be exactly the same as sum of the privatized benefit and the cost of signaling, *C*_*G*_ = *e* + *C*_*A*_, which for the same arguments present earlier this condition is quite implausible.

Moreover, we found that if the per capita privatized benefit offsets the per capita good’s cost that is QS-regulated, *qL*(*C*_*G*_−*e*) < 0, then *aG* always outcompetes *Ag* (Fig 5C and 5F). Otherwise, if the per capita privatized benefit does not offset the per capita good’s cost that is QS-regulated, *qL*(*C*_*G*_−*e*) ≥ 0, then three outcomes are possible.

First, *Ag* always outcompetes *aG* (line above x-axis). This will happen if the per capita good’s cost is higher than the sum of the per capita privatized benefit and the per capita autoinducer’s cost regulated by the QS-independent mechanism (Fig 5A and 5B), i.e., *q*_0_ (*C*_*G*_−*e*−*C*_*A*_) > 0. *Ag* also always outcompetes *aG* if good’s synthesis is not regulated by the QS-independent mechanisms (Fig 5E), i.e., *q*_0_ (*C*_*G*_−*e*−*C*_*A*_) = 0.

Second, *aG* always outcompetes *Ag* (line below x-axis). This will happen if the metabolic balance of QS-independent regulation is larger than the metabolic balance caused by QS-dependent regulation (Fig 5G and 5H, solid line), i.e., *q*_0_ (*C*_*G*_−*e*−*C*_*A*_) > *qL*(*C*_*G*_−*e*). The metabolic balance of QS-independent regulation is the total difference between the per capita good’s cost and the sum of the per capita privatized benefit and the per capita autoinducer’s cost resulting from QS-independent regulation. The metabolic balance of QS regulation is the total difference between the per capita good’s cost and the per capita privatized benefit resulting from QS regulation.

Third, the common strain outcompetes the rare strain (i.e., positive frequency-dependent selection). This will happen if the metabolic balance of QS-independent regulation is smaller than the metabolic balance caused by QS-dependent regulation, i.e., *q*_0_ (*C*_*G*_−*e*−*C*_*A*_) < *qL*(*C*_*G*_−*e*) (Fig 5H, dotted line). This condition reveals that coexistence between both strains is always unstable because: (1) the more autoinducers there are, the larger will be the overall net deficit on good production (*C*_*G*_ > *e*) favoring *Ag* over *aG*; (2) the cost of autoinducer production by *Ag* is constitutive and cannot outweigh the net deficit on good production.

### (E) Analysis of a population in which all the four strains exist

**Fig 6:**
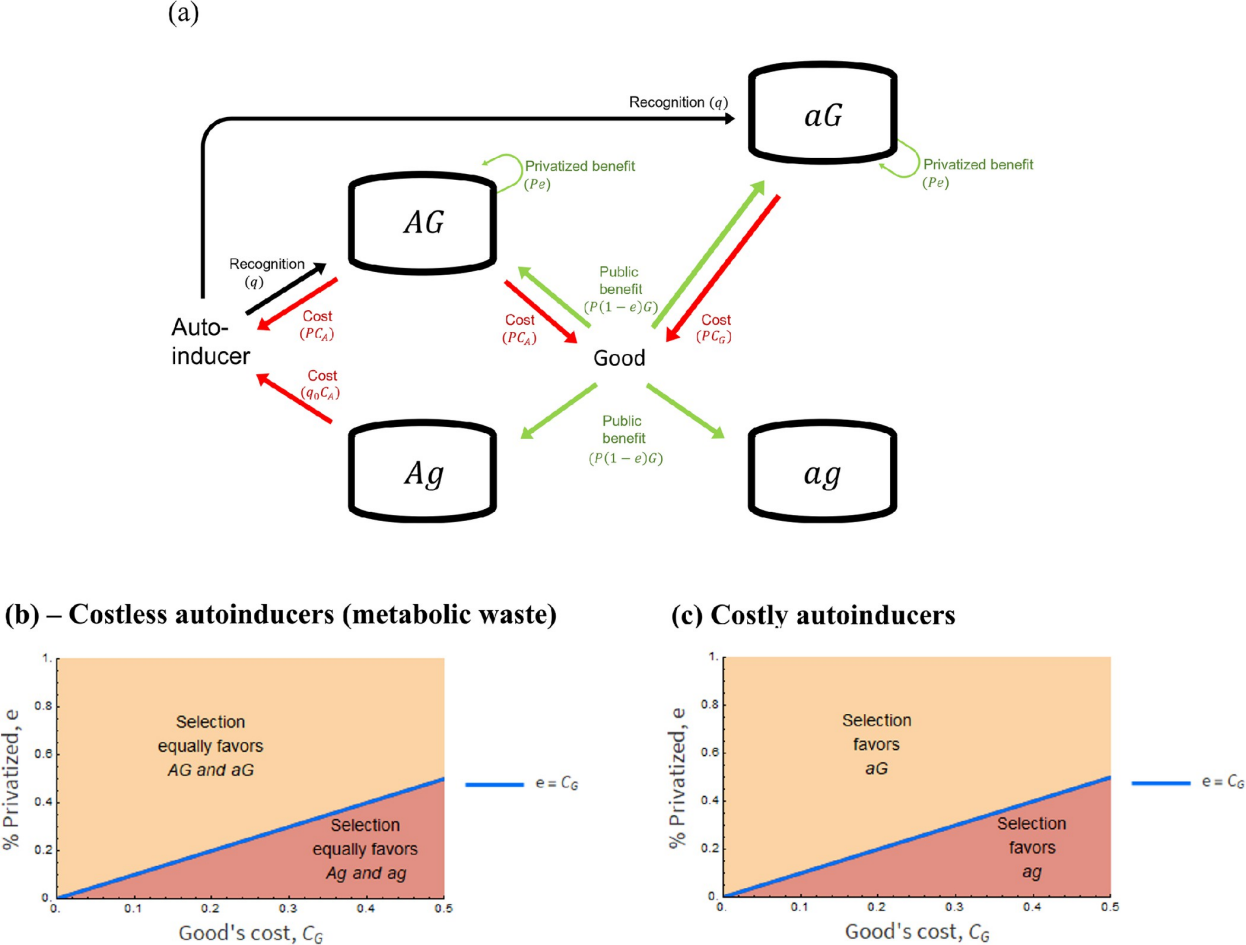
When all strains interact, partial privatization only favors QS if autoinducers are costless. **(a)** A schematic relationship of when all strains are simultaneously interacting. **(b)** With costless autoinducers, partial privatization favors QS. Selection equally favors *AG* and *aG* (yellow area) or *Ag* and *ag* (red area). Selection equally favors *AG* and *aG* if the per capita partially privatized benefit offsets the per capita good’s cost (*e* > *C*_*G*_). Otherwise (*e* < *C*_*G*_), selection equally favors *Ag* and *ag*. When signals are costless, *AG* and *aG* are equally fit and *Ag* and *ag* are equally fit. This result holds for 0 ≤ *L* ≤ 1, 0 ≤ *e* ≤ 1, 0 < *q*_0_ < 0.5, 0 ≤ *q* ≤ 0.5, 0 ≤ *C*_*G*_ < 0.5, and *C*_*A*_ = 0. **(c)** With costly autoinducers, partial privatization cannot favor QS. Selection either favors pure populations of *aG* (yellow area) or pure populations of *ag* (red area). Selection always favors pure populations of *aG* if the per capita partially privatized benefit offsets the per capita good’s cost (*e* > *C*_*G*_). Otherwise (*e* < *C*_*G*_), selection always favors pure populations of *ag*. This result holds for 0 ≤ *L* ≤ 1, 0 ≤ *e* ≤ 1, 0 < *q*_0_ < 0.5, 0 ≤ *q* ≤ 0.5, 0 ≤ *C*_*G*_ < 0.5, and 0 < *C*_*A*_ < 0.5. The blue line is when *aG* and *ag* are equally fit. Please see Table 2 and Section 6E for the definition of letters (*q*, *C*_*A*_ etc.) and the equations used for the analysis, respectively. All mathematical analyses assumed an unstructured population.

Above, we presented the analytical evolutionary outcome for pairwise interactions. However, the evolutionary dynamic in pairwise interactions need not be the same as when all strains are simultaneously interacting [16]. Here, we analyzed whether partial privatization favors QS when all strains are simultaneously interacting (Fig 6A). To analyze whether QS can be maintained in a population, we examined if selection eliminates alleles *A* and *G* from the population, which would occur if *AG* were eliminated and if both *Ag* and *aG* were eliminated.

We found that partial privatization cannot favor QS if autoinducers are costly. This is because selection leads to the loss of either allele *A* or both *A* and *G* alleles. On the one hand, if autoinducers are costless, selection will either favor *AG* and *aG* equally, or *Ag* and *ag* (Fig 6B). Under costless autoinducers, both *AG* and *aG* have the same access to shared and private benefits and costs on good production; hence *AG* and *aG* are equally fit. Likewise, both *Ag* and *ag* can only access shared benefits without incurring in any costs; hence *Ag* and *ag* are equally fit. Together, good producers (*AG* and *aG*) are favored over good non-producers (*Ag* and *ag*) if the per capita partially privatized benefit offsets the per capita good’s costs. Otherwise, selection favors good non-producers over good producers.

On the other hand, if selection is costly, selection either favors pure populations of *aG* or *ag* (Fig 6C). This occurs because *aG* has the same access to benefits as *AG*, but *aG* does not bear any autoinducer costs, while *AG* does. Similarly, *ag* and *Ag* have the same access to benefits, but *ag* does not bear any autoinducer costs, while *Ag* does. Together, selection favors pure populations of *aG* if the per capita partially privatized benefit offsets the per capita good’s costs. Otherwise, selection favors pure populations of *ag*.

After comparing these results with the pairwise interaction models, we noticed that pure populations of *AG*, pure populations of *Ag*, and mixed populations of *AG* and *Ag* are no longer favored by selection.

## 3 Discussion

Our work is the first to analyze the role of partial privatization in quorum sensing (QS) evolution. Our analytical results suggest partial privatization might have favored a primordial form of QS—where autoinducers were a metabolic byproduct, i.e., costless. Nevertheless, partial privatization cannot explain the maintenance of QS systems, as signaling is costly. Our results show that when autoinducers are costly, an *autoinducer-producer/good-producer* (fully producing, *AG*) strain is always eliminated from the population. Moreover, independently of autoinducers being costly or costless, our results indicated that partial privatization could not favor the coexistence of an *autoinducer-nonproducer/good-producer* (*aG*) and an *autoinducer-producer/good-nonproducer* (*Ag*), i.e., the metabolic specialization of QS. Together, our results differ from the earlier Black Queen Hypothesis’ predictions in which partially privatized benefits favor fully producing strains and metabolic specialization.

We found that partial privatization of a good’s benefit cannot favor QS via a fully producing strain (Fig 6). This result occurs because both the fully producing strain (*AG*) and the *autoinducer-nonproducer/good-producer* strain (*aG*) access partially privatized benefits, but only the fully producing strain pays signaling costs (Eq 8). Thus, whenever the fully producing strain is interacting with *aG*, selection cannot favor the fully producing strain. Moreover, our finding that partial privatization favors QS when autoinducers are costless suggests that partial privatization might have played a crucial role in the evolutionary origins of QS. That is because autoinducers are thought to be metabolic byproducts (costless) that were co-opted for communication [22–26, 28]. Taken together, the evolutionary origin of autoinducers and their transition from costless to costly molecules requires further research.

Sociomicrobial experiments are tightly associated with gram-negative bacteria, which typically produce AI2 and HSL autoinducers [9, 19–21, 29–38]. AI2 and HSL are small molecules relative to goods regulated by QS [39, 40], such as elastase and exopolysaccharides. Hence, autoinducers are usually considered relatively cheap to produce, and far less metabolically costly than the production of QS-regulated goods [41, 42]. Evolutionary mathematical models incorporated cheap autoinducers by assuming that signaling is costless [40, 43], coupling the cost of signaling and good production [9, 44], having the cost of signaling strictly lower than the cost of good production [45, 46], and not incorporating defective strains on signaling production [28, 42, 47]. These models provided foundational insights into the evolutionary transition from costless to cheap autoinducers (HSL and AI2). Nevertheless, these assumptions might not reflect the whole spectrum of QS evolution because gram-positive bacteria produce oligopeptides as autoinducers [48], which are far larger molecules than HSL and AI2. These oligopeptide autoinducers can be more than 20-fold costlier than HSL and AI2 autoinducers [18]. However, mathematical models incorporating the possibility of highly cost signaling relative to cooperation are far limited [49]. By incorporating different degrees of costly autoinducer production, our model revealed two main findings. First, that partial privatization can maintain QS for costless autoinducers but not costly ones. Second, the evolutionary outcomes from highly costly autoinducers are not found on slightly costly autoinducers. Specifically, for highly costly autoinducers, we found a negative frequency-dependent selection between *AG* and *Ag*, and positive frequency-dependent selection between *Ag* and *aG*.

Although partial privatization is ineffective against the *aG* strain, partial privatization favors the fully producing strain (*AG*) against the other two cheating strains (a*g* and *Ag*). This result occurs whenever the partially privatized benefit outweighs the cost of producing the mixed good and the autoinducer (Fig 1B and 2B). Additionally, our results show that negative frequency-dependent selection enables the stable coexistence between *AG* and *Ag* (blue area in Figs 2B and S1). The negative frequency-dependent selection is known to stabilize coexistence in nature [27, 50–52], which was also shown in some studies on QS evolution [9, 20] and the mixed good-producing strain’s evolution [5, 12, 14]. In *P*. *aeruginosa*, experiments with the partial privatization of siderophores support our conclusion [7].

Here, we also found that partial privatization cannot favor QS (the coexistence of *A* and *G* alleles) via metabolic specialization. Specifically, QS cannot be maintained by the coexistence between one strain specializing in autoinducer production (*Ag*) and one strain specializing in responding to it by producing a mixed good (*aG*) (Figs 5 and S1). This prediction is in accordance with the absence of QS specialization in natural systems and experiments [9, 53]. Also, our prediction agrees with the absence of a population that is mostly composed of *Ag-* and *aG*-like strains. [9, 53]. However, the strength of this result is inconclusive as previous studies addressing pairwise interactions [6, 7, 9, 19, 20, 31, 41, 54–56] have not tested the pairwise interaction of *Ag-* and *aG*-like strains. Additionally, we found that a three-way interaction among *Ag*, *aG*, and *ag* does not alter predictions found when all strains are simultaneously interacting (SI). The inexistence of stable 3-way interactions, found on classical rock-paper-scissors games, results from *ag* always outcompeting *Ag*, as both have the same access to benefits but only *Ag* produces costly autoinducers.

Why is metabolic specialization supported in previous models but not in ours [12, 13, 17]? There are two possible reasons that may explain this. First, while we have one mixed good and one autoinducer whose production is QS-dependent and QS-independent, earlier Black Queen models had two mixed goods being produced without the regulation by QS. Second, in earlier studies, metabolic specialization might occur because these models assumed that complementary strains are equally fit in their analysis [12, 13, 17]. While being equally fit is a justifiable assumption when each strain only produces one of two mixed goods, this assumption is unsuitable in in QS systems because while mixed goods generate benefits directly, autoinducers generate benefits indirectly through the goods that autoinducers regulate.

In our model, the effect of partial privatization was obtained by assuming linear benefits (the effect of benefits on bacteria is expressed as a linear function) and costs in an unstructured population. Therefore, a limitation of our model is that it does not include non-linear benefits/cost or the differential allocation of benefits towards genetically related individuals (i.e., kin selection). Earlier models (which assumed QS-independent regulation of mixed goods production) revealed coupling effects of nonlinear benefits from goods and kin selection. For instance, in yeast, while nonlinearity of benefits from sucrose metabolism explains selection favoring the coexistence between cooperators and cheaters, linearity of benefits does not [3]. Moreover, the coupled effect of partial privatization and spatial allocation of goods favored cooperation more than partial privatization alone [12]. Additionally, earlier QS models (which assumed no partial privatization) revealed that nonlinearity [40, 43, 44] and kin selection [45, 46, 49] dramatically influence the direction and strength of selection. Thus, our result that an *aG* always outcompetes *AG* (Section 6C) might change if we consider the coupled effect of partial privatization and kin selection. It would be of interest to study QS evolution in our model incorporating nonlinearity and kin selection.

Our predictions provide an explanation to patterns found in experimental studies. For instance, in yeast, while 99% of the benefits produced by the hydrolysis of sucrose are equally shared among members of the population, the remaining 1% privatized is enough to favor cooperation [3]. This finding corroborates our general prediction that the relative cost to partially privatized benefits favor cooperation, but not the total amount of benefits privatized (Section 6A). Consequently, cooperators might increase their fitness relative to defectors by maintaining privatized benefits roughly unchanged while decreasing the costs of cooperation, as illustrated by the *E*. *coli* production of catalase KatG. KatG provides public and private benefits through its enzymatic detoxification of hydrogen peroxide. Under unstructured populations, evolved *E*. *coli* cooperators could increase their advantage over defective strains by losing some copies of katG genes while maintaining the same production rate of KatG. Alternatively, cooperators might also increase their fitness relative to defectors by maintaining costs roughly unchanged while increasing their privatized benefits, as illustrated by a recent experiment with the *P*. *aeruginosa* production of pyoverdine [7]. Under increasing stress by reactive oxygen species (ROS), the cooperative pyoverdine-producing strains increase their privatized benefits by reducing pyoverdine secretion [7]. The reducion of pyoverdine secretion protects cells from oxidative damage by chelating the released ferrous, which would otherwise catalyze intracellular reactive species (peroxide) into the highly toxic hydroxyl radical through the Fenton reaction [57, 58]. Conversely, in earlier experiments, where the privatized benefits of pyoverdine were non-existent or very limited (due to experimental designs controlling for ROS), cooperators were found to be outcompeted in unstructured populations [38, 59]. Despite pyoverdine being coregulated by QS-independent and -dependent mechanisms [29, 60–63], early research has focused on pairwise interactions between wild-type strains and strains only lacking the ability to produce pyoverdine [7, 38, 59].

For types of social interaction not yet tested in studies analyzing the role of partial privatization, the coregulation of fully public and fully private goods, pleiotropy, is a related biological topic that can provide insights. One well-studied case of pleiotropy is the QS-independent and -dependent coregulation of elastase (public good) and the Nuh cellular nucleosidase (private good) in *P*. *aeruginosa* [19, 33, 35]. Both elastase and Nuh provide cells the access to carbon and energy: elastase is secreted into the environment and catalysis casein which subproducts are available to nearby individuals; Nuh is kept intracellularly and catalysis adenosine. Experiments under unstructured populations show that in the competition between a wild-type and a LasR mutant, analogous to *AG* and *Ag*, cooperation is favored the higher the relevance of Nuh [19, 35]. In the competition between a wild-type and a LasI mutant, analogous to *AG* and *aG*, however, a LasI mutant always outcompeted a wild-type strain in unstructured populations [19]. This occurs because (1) both strains can produce both private and public benefits, (2) they have equal access to secreted molecules, and (3) only wild-type strains pay the signaling costs. Despite these results emulating our predictions, given the mathematical similarity of pleiotropy and partial privatization [64], our predictions also indicate caution in generalizing findings in pairwise interactions, as these might not reflect evolution when more than two strains are simultaneously interacting (Section 6B and 6E).

In accordance with recent empirical findings [8], we assumed an increasing graded production of goods in response to autoinducer frequency, a QS generalized reciprocity strategy [9]. One alternative to generalized reciprocity is a regulatory network with high autoinducer production and low response (coercive strategy). This coercive strategy emerged on an agent-based *in silico* model for QS strains competing against defective strains unable to produce both autoinducers and goods [65]. Like generalized reciprocity, coercive strategy favors cooperation more than expected with kin selection alone [9, 65]. Given the potential relevance of coercive strains, could the inclusion of a coercive strain alter our conclusions? In our study, coercive strains would be an intermediary phenotype between *AG* and *Ag* because: (1) like *AG*, a coercive strain could maintain an equivalently high autoinducer production, higher than *Ag*; (2) coercive produce fewer goods than *AG* but more goods than *Ag*. Because our study analyzed the evolution of unstructured populations microbial, where autoinducers cannot cause differential production of goods and goods are equally accessible, coercive strains might not be able to alter our major conclusions. As a coercive strain would have the same access to shared goods as any other strain, *aG* might always outcompete a coercive strain, as both have access to privatized benefits, but only a coercive strain would pay signaling costs. Nevertheless, under structured populations, the joint effect of partial privatization, kin discrimination, and kin selection might lead to coercive strains to generate non-obvious evolutionary dynamics.

## 4 Conclusion

Our analytical results show how partial privatization affects the evolution of quorum sensing (QS). We have shown that unless autoinducers are costless partial privatization cannot explain why selection favors QS in a large unstructured population. However, the inability of partial privatization to favor QS (when autoinducers are costly) does not imply that future studies should neglect partial privatization. That is because our results also revealed that the outcomes of selection when partial privatization is present or absent are not necessarily the same. On the one hand, if privatized benefits cannot offset the production costs, the outcome of selection is expected to be qualitatively similar. On the other hand, if privatized benefits offset production costs, selection with and without partial privatization are expected to be qualitatively different. Together, our study suggests that new studies are needed to evaluate the potential underrepresentation of partial privatization on QS evolution.

## 5 Model

### Model framework

We consider a large clonally reproducing haploid population, large enough that the probability of a loss of a rare gene by random fluctuations is negligible. The population has discrete non-overlapping generations. To single out the effect of partial privatization, we assume that autoinducers and mixed goods are homogenously distributed across the environment at all times. We also assume that social interactions occur in an unstructured population; that is, cells are homogeneously distributed and there is no migration.

Using the standard population genetic framework [16], we track the change in strains’ frequency through time

$$x_{ij,t+1}=x_{ij,t}w_{ij,t}/\bar{w},$$ (1)

where, *x*_*ij*,*t*_ is the frequency of strain *ij* at time *t*, *i* = {*A*, *a*} and *j* = {*G*, *g*}. *w*_*ij*_ is the fitness of strain *ij* at time *t*. $\bar{w}$ is the population mean fitness. This system of difference equations was analytically solved in Mathematica (version 13), and its code is in the S1 File. Below we describe each strains’ behavior and fitness.

### Strains and fitnesses

We consider two types of secreted molecules, autoinducers and mixed goods. Mixed goods can produce benefits by promoting growth and by reducing mortality. Here, we assumed that the mixed good promotes growth. We assume that there is no external source of autoinducers and mixed goods, i.e., these two molecules are only biologically produced.

In natural systems, the more autoinducers are present in the environment, the higher the secretion of autoinducers (positive feedback loop). We indirectly modeled this positive feedback loop by assuming that *AG* always secretes more autoinducer than *Ag*. We did this by assuming that both *AG* and *Ag* secrete a QS-independent fraction of autoinducers, *L*, but only *AG* produces the fraction of autoinducers regulated by QS-dependent regulation, (1−*L*). Thus, the frequency of autoinducers in the environment at time *t*, *A*_*t*_, is the sum of QS-independent, *L* (*x*_*AG*,*t*_ + *x*_*Ag*,*t*_), and QS-dependent, (1−*L*)*x*_*AG*,*t*_ production of autoinducer

$$A_{t}=L\left(x_{AG,t}+x_{Ag,t}\right)+(1-L)x_{AG,t}.$$ (2)

QS-independent and QS-dependent regulation affects the total amount of autoinducers, and goods produced. Let *qA*_*t*_ be the probability of gene activation given an autoinducer’s frequency. Let *q* be the constant rate at which an autoinducer and a good are produced given *A*_*t*_. That is, *q* represents the influence of QS regulation, the ability of kin discrimination. Based on empirical findings that some QS-regulated mixed goods are co-regulated by QS-independent mechanisms [33, 66], we consider the constant rate *q*_0_. Thus, the rate of a costly autoinducer’s and a good’s production is

$$P=q_{0}+qA_{t}.$$ (3)

Given that 0 ≤ *A*_*t*_ ≤ 1, we ensure that *P* varies between 0 and 1, by having 0 ≤ *q*_0_, *q* ≤ 1/2.*q*_0_ = 0 indicates the lack of QS-independent regulation. *q* = 0 indicates absence of QS regulation. The term *qA*_*t*_ is in accordance with two empirical findings. First, that the rate of production regulated by quorum sensing depends on the frequency of strains carrying A allele in the population ([9]). Second, that the populational rate of production is graded [8]. We allow *q*_0_ to be larger than *q* because research found that the regulatory genetic architecture can evolve relatively fast to become less reliant on QS [32].

We assume that the production of the autoinducer has a per capita cost *C*_*A*_, 0 ≤ *C*_*A*_ < 0.5. The production of the good has a per capita cost *C*_*G*_, 0 ≤ *C*_*G*_ < 0.5. The total cost paid by each strain *ij*, *c*_*ij*_, dependents on the strain’s ability to produce autoinducers and goods and on its ability to recognize autoinducers. Let *i* = {*A*, *a*} and *j* = {*G*, *g*}. Because *ag* neither produce autoinducers nor goods, *ag* has no costs, *c*_*ag*_ = 0. *Ag* only produces autoinducers constitutively and does not produce goods; hence this strain pays the cost *c*_*Ag*_ = *q*_0_*C*_*A*_. *aG* does not produce autoinducers and produces goods constitutively and proportionally to autoinducer frequency; hence this strain pays the cost *c*_*aG*_ = *PC*_*G*_. Lastly, *AG* produces autoinducers and goods both constitutively and proportionally to autoinducer frequency; hence this strain pays the cost *c*_*AG*_ = *P*(*C*_*G*_ + *C*_*A*_).

The frequency of goods in the environment is *PG*_*t*_.*G*_*t*_ is the frequency of allele *G* at time *t*, i.e., *G*_*t*_ = *x*_*AG*,*t*_ + *x*_*aG*,*t*_. The benefit generated by allele *G* is unity. Let *e* (0 ≤ *e* ≤ 1) be the fraction of the benefit that is of exclusive access to producers, i.e., the percentage of partially privatized benefit. *e* = 0 implies that the whole benefit is shared, i.e., fully public. *e* = 1 implies that the whole benefit is private. (1−*e*) is the fraction of benefit that is public. Thus, 1*e* and 1(1−*e*)*G* are the per capita private and the per capita public benefit, respectively. *Pe* and *P*(1−*e*)*G* are the total private and the total public benefits produced, respectively.

Let *b*_*AG*_ be the total benefit an *ij* strain has. Because we assume an unstructured population, all individuals have access to the public benefit. Because *ag* does not produce goods, its benefit is *b*_*ag*_ = *P*(1−*e*)*G*. Because *Ag* also does not produce goods, its benefit is *b*_*Ag*_ = *P*(1−*e*)*G*. Because *aG* produces goods, its benefit is *b*_*aG*_ = *P*[(1−*e*)*G* + *e*]. Lastly, because *aG* produces goods, its benefit is *b*_*AG*_ = *P*[(1−*e*)*G* + *e*]. We assume a baseline fitness of unity. Taking together, the fitnesses equations are

$$w_{ij}=1+b_{ij}-c_{ij}.$$ (4)

The list of parameters and variables is found in Table 2.

## 6 Analytical solutions

Below we present details about the analytical solutions for the interactions considered in this work. All analytical solutions were run in Mathematica (version 13), and its code is in the S1 File.

### (A) Pairwise interaction between *AG* and *ag* strains

Let *A*_*t*_ = *x*_*AG*,*t*_ be the autoinducer’s frequency at time *t*. Let *G*_*t*_ *= x*_*AG*,*t*_ be the maximum good’s frequency in the environment at time *t*. The relative fitness of *AG* (*w*_*AG*_ > *w*_*ag*_) is

$$\left(q_{0}+qx_{AG,t}\right)\left(e-C_{G}-C_{A}\right)>0.$$ (5)

Selection favors pure populations of *AG* (*x*_*AG*_ = 1) if *e* > *C*_*G*_ + *C*_*A*_, *q*_0_ ≠ 0 and *q* ≠ 0. Selection favors pure populations of *ag* (*x*_*ag*_ = 1) if *e* < *C*_*G*_ + *C*_*A*_ and *q*_0_ ≠ 0.

### (B) Pairwise interaction between AG and *Ag* strains

Let *A*_*t*_ = *L* + (1−*L*)*x*_*AG*,*t*_ be the autoinducer’s frequency at time *t*. Let *G*_*t*_ = *x*_*AG*,*t*_ be the maximum good’s frequency at time *t*. The relative fitness of *AG* (*w*_*AG*_ > *w*_*Ag*_) is

$$q_{0}\left(e-C_{G}\right)+qA_{t}(e-C_{G}-C_{A})>0.$$ (6)

Selection favors three possible outcomes. First, a pure population of *AG* (*x*_*AG*_ = 1) is stable if $e-C_{G}>C_{A}\frac{q}{q_{0}+q}$. Second, a pure population of *Ag* (*x*_*Ag*_ = 1) is stable if $e-C_{G}<C_{A}\frac{qL}{q_{0}+qL}$. Lastly, a mixed population of *AG* and *Ag* $\left(x_{AG}=\frac{\frac{q_{0}(C_{G}-e)}{q(C_{G}+C_{A}-e)}+L}{-1+L}\right)$ is stable if $C_{A}\frac{qL}{q_{0}+q_{L}}<e-C_{G}<C_{A}\frac{q}{q_{0}+q}$.

### (C) Pairwise interaction between *AG* and *aG* strains

Let *A*_*t*_ = *x*_*AG*,*t*_ be the autoinducer’s frequency at time *t*. Let *G*_*t*_ = *x*_*AG*,*t*_ + *x*_*aG*,*t*_ = 1 be the maximum good’s frequency at time *t*. The difference in fitness between *AG* and *aG* is

$$w_{AG}-w_{aG}=-(q_{0}+qx_{AG,t})C_{A}.$$ (7)

Thus, selection always favors pure populations of *aG* (*x*_*aG*_ = 1), unless the production of autoinducer is costless (*C*_*A*_ = 0), which implies that both strains are equally fit.

### (D) Pairwise interaction between functionally complementary strains (i.e., *Ag*, and *aG*)

Let *A*_*t*_ = *Lx*_*Ag*,*t*_ be the autoinducer’s frequency at time *t*. Let *G*_*t*_ = *x*_*aG*,*t*_ be the maximum good’s frequency at time *t*. The relative fitness of *Ag* (*w*_*Ag*_ > *w*_*aG*_) is

$$q_{0}(C_{G}-e-C_{A})+qL(C_{G}-e)x_{Ag}>0.$$ (8)

We found that if the per capita private benefit offsets the per capita good’s cost (*C*_*G*_ < *e*), then *aG* always outcompetes *Ag* (i.e., *w*_*aG*_ > *w*_*Ag*_ is always true) (Fig 5C and 5F). Otherwise (*C*_*G*_ < *e*), selection can generate one of three possible outcomes.

First, *Ag* always outcompetes *aG* (Fig 5B and 5E). This happens if *qL*(*C*_*G*_−*e*) ≥ 0 and *q*_0_ (*C*_*G*_−*e*−*C*_*A*_) ≥ 0. Meaning, if the autoinducer cost paid by *Ag* is not enough to cause the net deficit in producing goods (*C*_*G*_ > *e*) paid by *aG*.

Second, *aG* always outcompetes *Ag* (Fig 5H, solid line). This happens if *qL*(*C*_*G*_−*e*) ≥ 0, *q*_0_ (*C*_*G*_−e−C_A_) < 0 and *qL*(*C*_*G*_−*e*) < *q*_0_ (*C*_*G*_−*e*−*C*_*A*_). That is, if the difference between the per capita good’s cost is lower than the sum of the per capita privatized benefit and the per capita autoinducer’s cost and if the total of this metabolic balance caused by QS-independent regulation, *q*_0_ (*C*_*G*_−e−C_A_) < 0, is larger than the metabolic balance coming from the QS-regulation generating a per capita good’s cost and a per capita privatized benefit, *qL*(*C*_*G*_−*e*) < *q*_0_ (*C*_*G*_−*e*−*C*_*A*_).

Third, positive frequency-dependent selection favors the most common strain (Fig 5H, dotted line). This happens if *qL*(*C*_*G*_−*e*) ≥ 0, *q*_0_ (*C*_*G*_−e−C_A_) < 0 and *qL*(*C*_*G*_−*e*) > *q*_0_ (*C*_*G*_−*e*−C_A_). That is, if the metabolic balance of QS-independent regulation is smaller than the metabolic balance caused by QS regulation.

### (E) All strains simultaneously interacting

Let *A*_*t*_ = *L* (*x*_*AG*,*t*_ + *x*_*Ag*,*t*_) + (1−*L*)*x*_*AG*,*t*_ and *G*_*t*_ = *x*_*AG*,*t*_ + *x*_*aG*,*t*_ be the autoinducer’s and good’s frequency in the environment at time *t*.

From the competition among all strains, if autoinducers are costless, *w*_*AG*_ = *w*_*aG*_ and *w*_*Ag*_ = *w*_*ag*_. Moreover, selection equally favors *AG* and *aG* if *e* > *C*_*G*_. Otherwise (*e* < *C*_*G*_), selection equally favors *Ag* and *ag*. Nevertheless, if autoinducers are costly, *w*_*aG*_ ≥ *w*_*AG*_ and *w*_*ag*_ ≥ *w*_*Ag*_. Moreover, selection will either drive evolution towards pure populations of *aG* or *ag*. Selection favors pure populations of *aG* (*x*_*aG*_ = 1) if *e* > *C*_*G*_. Otherwise (*e* < *C*_*G*_), selection favors pure populations of *ag* (*x*_*ag*_ = 1).

## Supporting information

S1 FigThe partially privatized benefit favors QS through pure populations of *AG* or through mixed populations of *AG* and *Ag*.Selection either favors (i) pure populations of *AG* (blue area); (ii) pure populations of *Ag* (brown area); (iii) coexistence of *AG* and *Ag*. Selection favors the coexistence if the rare strain outcompetes the common one (i.e., negative frequency-dependent selection). Negative frequency-dependent selection emerges from the co-regulation of QS (*q*) and QS-independent (*q*_*0*_) mechanisms, the existence of privatization (*e*) and having privatized benefits offsetting the minimum autoinducer’s cost, $C_{A}\frac{qL}{q_{0}+qL}$, but not the maximum autoinducer’s cost, $C_{A}\frac{q}{q_{0}+q}$. The x-axis is the per capita autoinducer’s cost, *C*_*A*_. The y-axis is the difference between the per capita partially privatized benefit and the per capita good’s cost, *e*−*C*_*G*_. Each subgraph captures the effect of regulatory architecture, via QS-dependent and QS-independent mechanisms. *q* = 0 implies absence of QS regulation. *q*_0_ = 0 implies absence of QS-independent regulation. Parameters: *C*_*G*_ = 0.3, *L* = 0.1. Initial frequency of each strain was draw from a uniform distribution. The simulation was stopped after 10000 steps.(PDF)

S1 File (ZIP)
