# Supplementary figures and images for: Black Queen Hypothesis, partial privatization, and quorum sensing evolution
Source: PLoS One. 2022 Nov 30;17(11):e0278449. doi: 10.1371/journal.pone.0278449 (PMC9710793; doi:10.1371/journal.pone.0278449)

% Privatized – Good's cost,  $e - C_G$

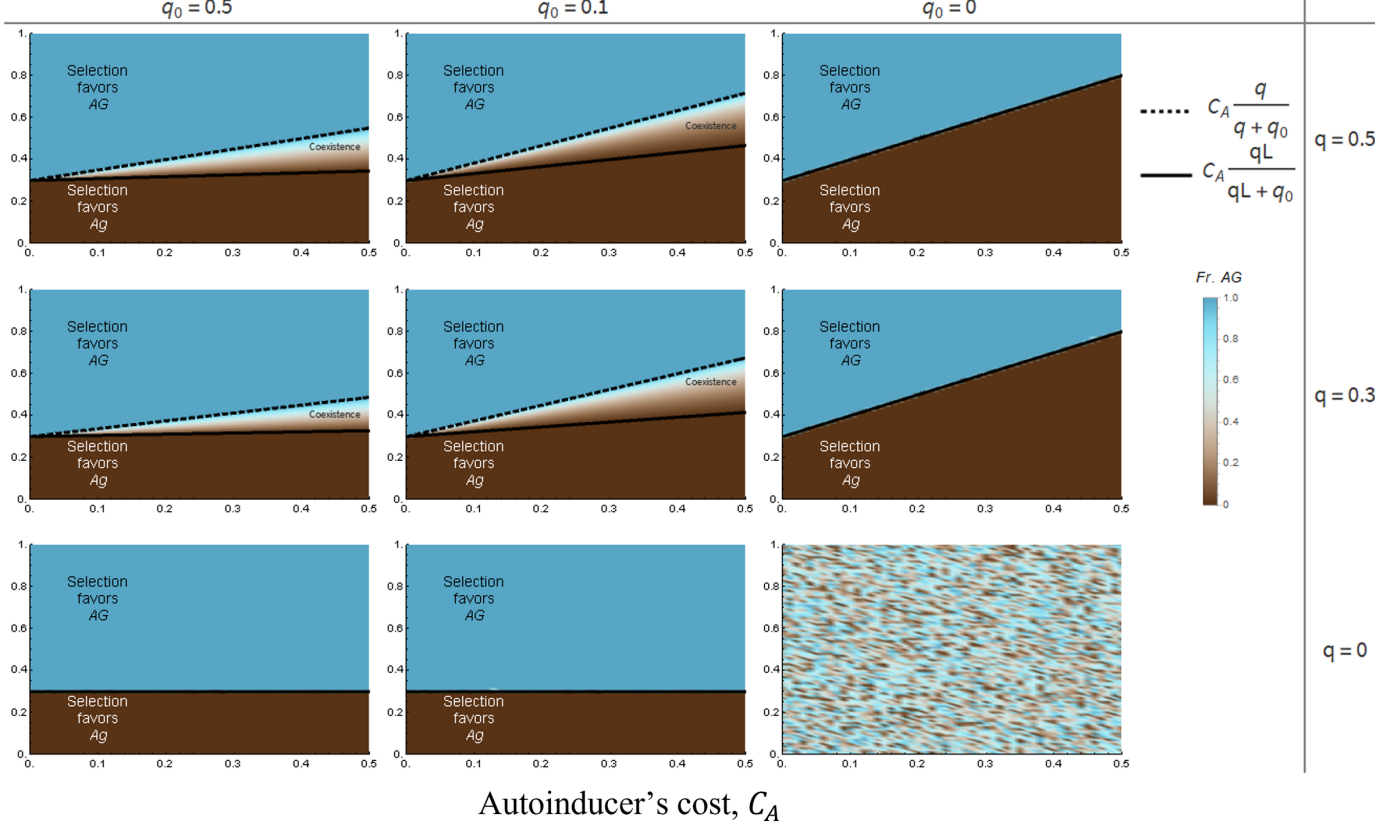

Supplement: S1 Fig — Selection either favors (i) pure populations of AG (blue area); (ii) pure populations of Ag (brown area); (iii) coexistence of AG and Ag. Selection favors the coexistence if the rare strain outcompetes the common one (i.e., negative frequency-dependent selection). Negative frequency-dependent selection emerges from the co-regulation of QS (q) and QS-independent (q0) mechanisms, the existence of privatization (e) and having privatized benefits offsetting the minimum autoinducer’s cost, CAqLq0+qL, but not the maximum autoinducer’s cost, CAqq0+q. The x-axis is the per capita autoinducer’s cost, CA. The y-axis is the difference between the per capita partially privatized benefit and the per capita good’s cost, e−CG. Each subgraph captures the effect of regulatory architecture, via QS-dependent and QS-independent mechanisms. q = 0 implies absence of QS regulation. q0 = 0 implies absence of QS-independent regulation. Parameters: CG = 0.3, L = 0.1. Initial frequency of each strain was draw from a uniform distribution. The simulation was stopped after 10000 steps. (PDF) [file pone.0278449.s001.pdf]
