# Supplementary material for: Black Queen Hypothesis, partial privatization, and quorum sensing evolution
Source: PLoS One. 2022 Nov 30;17(11):e0278449. doi: 10.1371/journal.pone.0278449 (PMC9710793; doi:10.1371/journal.pone.0278449)
Supplement: S1 File — (ZIP) [file pone.0278449.s002.zip › Code.pdf]

In[ ]:=
```
Remove["Global`*"];
Quit[]
```

Analytical solution for section 6 A - {*AG*, *ag*}

In[ ]:=
```
Remove["Global`*"];
Quit[]
```

In[ ]:=
```
At = L * (xAG[t] + xAg[t]) + (1 - L) * xAG[t] /. xAg[t] → 0 // Simplify
Gt = (xAG[t] + xaG[t]) /. xaG[t] → 0
P = q0 + q * At
```

Out[ ]= xAG[t]

Out[ ]= xAG[t]

Out[ ]= q0 + q xAG[t]

In[ ]:=
```
wAG = 1   + P * (e + (1 - e) * Gt)   - P (cG + cA)
wag = 1   + P * ((1 - e) * Gt)

 (*AG vs ag*)wAG - wag // FullSimplify
Manipulate[ Plot[% /. xAG[t] -> xAG, {xAG, 0, 1}, PlotRange → {{0, 1}, {-1.11, 1.11}} ],
  {q0, 0, 1/2}, {q, 0, 1/2}, {e, 0, 1}, {cG, 0, 1/2}, {cA, 0, 1/2}]
```

Out[ ]= 1 - (cA + cG) (q0 + q xAG[t]) + (e + (1 - e) xAG[t]) (q0 + q xAG[t])

Out[ ]= 1 + (1 - e) xAG[t] (q0 + q xAG[t])

Out[ ]= - (cA + cG - e) (q0 + q xAG[t])

Out[ ]=
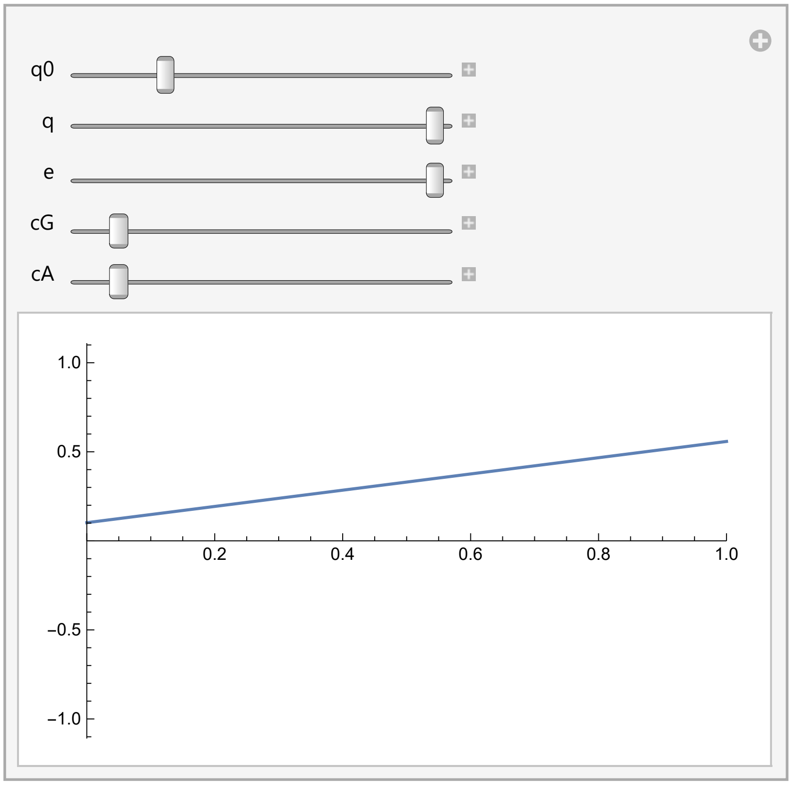

```
(*Mean Fitness*)
W =  wAG * xAG[t] + wag * xag[t] /. xag[t] → (1 - xAG[t]) // FullSimplify
W // Expand
Manipulate[ Plot[% /. xAG[t] -> xAG, {xAG, 0, 1}, PlotRange → {{0, 1}, {0, 2.5}} ],
 {q0, 0, 1/2}, {q, 0, 1/2}, (*{e,0,1},*){cG, 0, 1/2}, {cA, 0, 1/2}]
```

Out[ ]= $1 - (-1 + cA + cG)\, xAG[t]\, (q0 + q\, xAG[t])$

Out[ ]= $1 + q0\, xAG[t] - cA\, q0\, xAG[t] - cG\, q0\, xAG[t] + q\, xAG[t]^2 - cA\, q\, xAG[t]^2 - cG\, q\, xAG[t]^2$

Out[ ]=

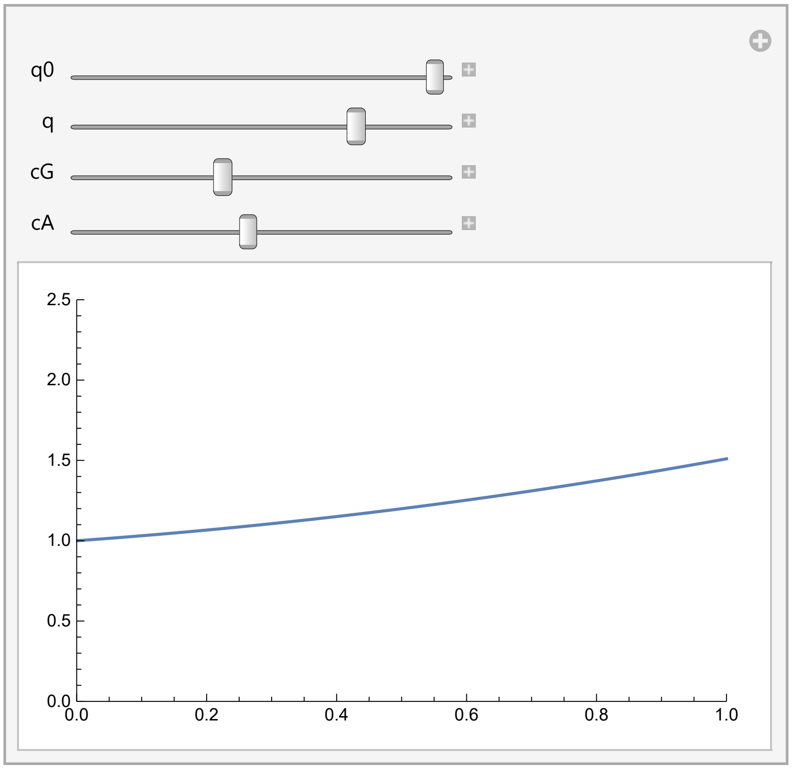

In[ ]:=
```
(*Plotting mean fitness (W) and wAG*)
W /. xAG[t] -> xAG
 wAG /. xAG[t] -> xAG
Manipulate[ Plot[{%% /. xAG[t] -> xAG, % /. xAG[t] -> xAG},
   {xAG, 0, 1}, PlotRange → {{0, 1}, {0, 2.5}} ],
 {q0, 0, 1/2}, {q, 0, 1/2}, {e, 0, 1}, {cG, 0, 1/2}, {cA, 0, 1/2}]
(*
legend:
   W→blue
   wAG→ yellow
*)
```

Out[ ]= $1 - (-1 + cA + cG)\, xAG\, (q0 + q\, xAG)$

Out[ ]= $1 - (cA + cG)\, (q0 + q\, xAG) + (e + (1 - e)\, xAG)\, (q0 + q\, xAG)$

Out[ ]=
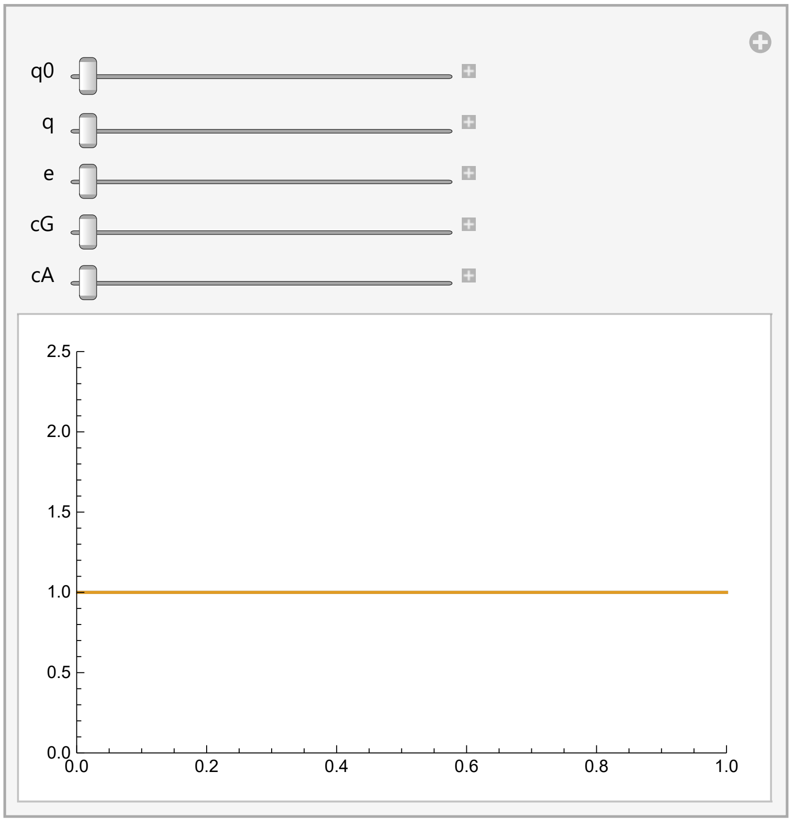

Recurrence - System of $\{x_{AG}, x_{ag}\}$

In[ ]:=
```
xag[t] = (1 - xAG[t]);

xAG[t + 1] = (xAG[t] * wAG / W) // FullSimplify
```

Out[ ]=
$$\frac{xAG[t]\ (1 - (cA + cG)\ (q0 + q\ xAG[t]) + (e + xAG[t] - e\ xAG[t])\ (q0 + q\ xAG[t]))}{1 - (-1 + cA + cG)\ xAG[t]\ (q0 + q\ xAG[t])}$$

Finding equilibrium

In[ ]:=
```
equil = Solve[{xAG[t + 1] - xAG[t] == 0}, {xAG[t]}];
equilibrium = equil // FullSimplify
equilibrium // MatrixForm
```

Out[ ]=
$$\left\{\{xAG[t] \to 0\}, \{xAG[t] \to 1\}, \left\{xAG[t] \to -\frac{q0}{q}\right\}\right\}$$

Out[ ]//MatrixForm=
$$\begin{pmatrix} xAG[t] \to 0 \\ xAG[t] \to 1 \\ xAG[t] \to -\frac{q0}{q} \end{pmatrix}$$

Condition for existence

In[ ]:=
```
xAG1 = xAG[t] /. equilibrium[[1]]
xAG2 = xAG[t] /. equilibrium[[2]]
xAG3 = xAG[t] /. equilibrium[[3]]
```

Out[ ]= 0

Out[ ]= 1

Out[ ]= $-\frac{q0}{q}$

In[∘]:=
```
Reduce[{ 0 ≤ xAG3 ≤ 1,
  0 <= e ≤ 1,
  0 <= L ≤ 1,
  0 <= cG < 1/2,
  0 <= cA < 1/2,
  0 ≤ q ≤ 1/2,
  0 ≤ q0 ≤ 1/2}, {q}]
```

Out[∘]= $0 \le e \le 1 \,\&\&\, 0 \le L \le 1 \,\&\&\, 0 \le cG < \frac{1}{2} \,\&\&\, 0 \le cA < \frac{1}{2} \,\&\&\, q0 == 0 \,\&\&\, 0 < q \le \frac{1}{2}$

Stability

Step 1) -Setting Jacobian matrix

In[∘]:=
```
Jac = D[xAG[t + 1], {xAG[t]}];
J = Jac // FullSimplify
J // MatrixForm;
```

Out[∘]= $\left(1-(cA+cG-e)\,q0+xAG[t]\left(-2\,(cA+cG-e)\,q-2\,(-1+e)\,q0+xAG[t]\left((2+cA+cG-3\,e)\,q+(-1+cA+cG)(-1+e)\,q0^2+(-1+cA+cG)(-1+e)\,q\,xAG[t]\,(2\,q0+q\,xAG[t])\right)\right)\right)\Big/\left(-1+(-1+cA+cG)\,xAG[t]\,(q0+q\,xAG[t])\right)^2$

1 JACOBIAN at {xAG[t]→0}

In[∘]:=
```
equilibrium[[1]]
J1 = J /. equilibrium[[1]] // FullSimplify
```

Out[∘]= {xAG[t] → 0}

Out[∘]= $1-(cA+cG-e)\,q0$

In[∘]:=
```
Reduce[{ -1 < J1 < 1,
   0 <= e ≤ 1,
   (*0<= L≤ 1,*)
   0 <= cG < 1/2,
   0 <= cA < 1/2,
   (*0≤ q≤ 1/2,*)
   0 ≤ q0 ≤ 1/2}, {e}] // FullSimplify
```

Out[∘]= $0 < q0 \le \frac{1}{2} \,\&\&\, 2\,cA < 1 \,\&\&\, e \ge 0 \,\&\&\, ((cG == 0 \,\&\&\, cA > 0 \,\&\&\, e < cA) \,||\, (cG > 0 \,\&\&\, 2\,cG < 1 \,\&\&\, e < cA + cG \,\&\&\, cA \ge 0))$

In[ ]:=
```
Reduce[{ -1 < J1 < 1,
   0 <= e ≤ 1,
   (*0<= L≤ 1,*)
   0 <= cG < 1/2,
   0 <= cA < 1/2,
   (*0≤ q≤ 1/2,*)
   0 ≤ q0 ≤ 1/2}, {cG}] // FullSimplify
```

Out[ ]= $0 < q0 \leq \frac{1}{2}$ && 2 cG < 1 && ((2 e < 1 && e ≥ 0 && ((cA == e && cG > 0) || (cA + cG > e && cA < e && cA ≥ 0) || (2 cA < 1 && e < cA && cG ≥ 0))) || ($\frac{1}{2}$ + cA > e && cA + cG > e && 2 cA < 1 && 2 e ≥ 1))

In[ ]:=
```
Reduce[{ -1 < J1 < 1,
   0 <= e ≤ 1,
   (*0<= L≤ 1,*)
   0 <= cG < 1/2,
   0 <= cA < 1/2,
   (*0≤ q≤ 1/2,*)
   0 ≤ q0 ≤ 1/2}, {cA}] // FullSimplify
```

Out[ ]= $0 < q0 \leq \frac{1}{2}$ && 2 cA < 1 && ((2 e < 1 && e ≥ 0 && ((cG == e && cA > 0) || (cA + cG > e && cG < e && cG ≥ 0) || (2 cG < 1 && e < cG && cA ≥ 0))) || ($\frac{1}{2}$ + cG > e && cA + cG > e && 2 cG < 1 && 2 e ≥ 1))

In[ ]:=
```
Reduce[{ -1 < J1 < 1,
   0 <= e ≤ 1,
   (*0<= L≤ 1,*)
   0 <= cG < 1/2,
   0 <= cA < 1/2,
   (*0≤ q≤ 1/2,*)
   0 ≤ q0 ≤ 1/2}, {q0}] // FullSimplify
```

Out[ ]= $0 < q0 \leq \frac{1}{2}$ && 2 cA < 1 && ((2 e < 1 && e ≥ 0 && ((cG == e && cA > 0) || (cA + cG > e && cG < e && cG ≥ 0) || (2 cG < 1 && e < cG && cA ≥ 0))) || ($\frac{1}{2}$ + cG > e && cA + cG > e && 2 cG < 1 && 2 e ≥ 1))

2 JACOBIAN at {xAG[t]→1}

In[ ]:=
```
equilibrium[[2]]
J2 = J /. equilibrium[[2]] // FullSimplify
```

Out[ ]= {xAG[t] → 1}

Out[ ]= $\frac{-1+(-1+e)\,q+(-1+e)\,q0}{-1+(-1+cA+cG)\,q+(-1+cA+cG)\,q0}$

In[ ]:=
```
Reduce[{ -1 < J2 < 1,
   0 <= e ≤ 1,
   (*0<= L≤ 1,*)
   0 <= cG < 1/2,
   0 <= cA < 1/2,
   0 ≤ q ≤ 1/2,
   0 ≤ q0 ≤ 1/2}, {e}] // FullSimplify
```

Out[ ]= e ≤ 1 && 2 q ≤ 1 && ((q > 0 && q0 == 0) || (q ≥ 0 && 2 q0 ≤ 1 && q0 > 0)) &&
((cG == 0 && ((cA == 0 && e > 0) || (2 cA < 1 && cA > 0 && cA < e))) ||
(cG > 0 && cA ≥ 0 && 2 cA < 1 && 2 cG < 1 && cA + cG < e))

In[ ]:=
```
Reduce[{ -1 < J2 < 1,
   0 <= e ≤ 1,
   (*0<= L≤ 1,*)
   0 <= cG < 1/2,
   0 <= cA < 1/2,
   0 ≤ q ≤ 1/2,
   0 ≤ q0 ≤ 1/2}, {q0}] // FullSimplify
```

Out[ ]= e ≤ 1 && 2 q0 ≤ 1 && ((q == 0 && q0 > 0) || (2 q ≤ 1 && q > 0 && q0 ≥ 0)) &&
((cG == 0 && ((cA == 0 && e > 0) || (2 cA < 1 && cA > 0 && cA < e))) ||
(cG > 0 && cA ≥ 0 && 2 cA < 1 && 2 cG < 1 && cA + cG < e))

In[ ]:=
```
Reduce[{ -1 < J2 < 1,
   0 <= e ≤ 1,
   (*0<= L≤ 1,*)
   0 <= cG < 1/2,
   0 <= cA < 1/2,
   0 ≤ q ≤ 1/2,
   0 ≤ q0 ≤ 1/2}, {q}] // FullSimplify
```

Out[ ]= e ≤ 1 && 2 q ≤ 1 && ((q > 0 && q0 == 0) || (q ≥ 0 && 2 q0 ≤ 1 && q0 > 0)) &&
((cG == 0 && ((cA == 0 && e > 0) || (2 cA < 1 && cA > 0 && cA < e))) ||
(cG > 0 && cA ≥ 0 && 2 cA < 1 && 2 cG < 1 && cA + cG < e))

In[∘]:=
```
Reduce[{ -1 < J2 < 1,
   0 <= e ≤ 1,
   (*0<= L≤ 1,*)
   0 <= cG < 1/2,
   0 <= cA < 1/2,
   0 ≤ q ≤ 1/2,
   0 ≤ q0 ≤ 1/2}, {cA}] // FullSimplify
```

Out[∘]=

$$\left(q0 == 0 \,\&\&\, \left(\left(0 < q < \frac{1}{2} \,\&\&\, \left(\left(cG == 0 \,\&\&\, cA \geq 0 \,\&\&\, \left(\left(e > 0 \,\&\&\, cA < e \,\&\&\, 2\,e \leq 1\right) \,||\, \left(2\,e > 1 \,\&\&\, 2\,cA < 1 \,\&\&\, e \leq 1\right)\right)\right) \,||\, \left(0 < cG < \frac{1}{2} \,\&\&\, \left(\left(cA \geq 0 \,\&\&\, \frac{1}{2} + cG > e \,\&\&\, cA + cG < e\right) \,||\, \left(\frac{1}{2} + cG \leq e \leq 1 \,\&\&\, 0 \leq cA < \frac{1}{2}\right)\right)\right)\right)\right) \,||\, \left(2\,q == 1 \,\&\&\, 0 \leq cG < \frac{1}{2} \,\&\&\, \left(\left(cA \geq 0 \,\&\&\, \frac{1}{2} + cG > e \,\&\&\, cA + cG < e\right) \,||\, \left(\frac{1}{2} + cG \leq e \leq 1 \,\&\&\, 0 \leq cA < \frac{1}{2}\right)\right)\right)\right)\right) \,||\, \left(0 < q0 \leq \frac{1}{2} \,\&\&\, 0 \leq q \leq \frac{1}{2} \,\&\&\, 0 \leq cG < \frac{1}{2} \,\&\&\, \left(\left(cA \geq 0 \,\&\&\, \frac{1}{2} + cG > e \,\&\&\, cA + cG < e\right) \,||\, \left(\frac{1}{2} + cG \leq e \leq 1 \,\&\&\, 0 \leq cA < \frac{1}{2}\right)\right)\right)$$

In[∘]:=
```
Reduce[{ -1 < J2 < 1,
   0 <= e ≤ 1,
   (*0<= L≤ 1,*)
   0 <= cG < 1/2,
   0 <= cA < 1/2,
   0 ≤ q ≤ 1/2,
   0 ≤ q0 ≤ 1/2}, {cG}] // FullSimplify
```

Out[∘]=

$$\left(q0 == 0 \,\&\&\, \left(\left(0 < q < \frac{1}{2} \,\&\&\, \left(\left(cA == 0 \,\&\&\, cG \geq 0 \,\&\&\, \left(\left(e > 0 \,\&\&\, cG < e \,\&\&\, 2\,e \leq 1\right) \,||\, \left(2\,e > 1 \,\&\&\, 2\,cG < 1 \,\&\&\, e \leq 1\right)\right)\right) \,||\, \left(0 < cA < \frac{1}{2} \,\&\&\, \left(\left(cG \geq 0 \,\&\&\, \frac{1}{2} + cA > e \,\&\&\, cA + cG < e\right) \,||\, \left(\frac{1}{2} + cA \leq e \leq 1 \,\&\&\, 0 \leq cG < \frac{1}{2}\right)\right)\right)\right)\right) \,||\, \left(2\,q == 1 \,\&\&\, 0 \leq cA < \frac{1}{2} \,\&\&\, \left(\left(cG \geq 0 \,\&\&\, \frac{1}{2} + cA > e \,\&\&\, cA + cG < e\right) \,||\, \left(\frac{1}{2} + cA \leq e \leq 1 \,\&\&\, 0 \leq cG < \frac{1}{2}\right)\right)\right)\right)\right) \,||\, \left(0 < q0 \leq \frac{1}{2} \,\&\&\, 0 \leq q \leq \frac{1}{2} \,\&\&\, 0 \leq cA < \frac{1}{2} \,\&\&\, \left(\left(cG \geq 0 \,\&\&\, \frac{1}{2} + cA > e \,\&\&\, cA + cG < e\right) \,||\, \left(\frac{1}{2} + cA \leq e \leq 1 \,\&\&\, 0 \leq cG < \frac{1}{2}\right)\right)\right)$$

3 JACOBIAN at {xAG[t]→ $-\frac{q0}{q}$}

In[ ]:=
```
equilibrium[[3]]
xAG3 = xAG[t] /. equilibrium[[3, 1]]
J3 = J /. equilibrium[[3]] // FullSimplify
```

Out[ ]= $\left\{xAG[t] \to -\frac{q0}{q}\right\}$

Out[ ]= $-\frac{q0}{q}$

Out[ ]= $1 + \frac{(cA + cG - e)\, q0\, (q + q0)}{q}$

In[ ]:=
```
Reduce[{ -1 < J3 < 1,
    0 ≤ xAG3 ≤ 1,
    0 <= e ≤ 1,
    (*0<= L≤ 1,*)
    0 <= cG < 1/2,
    0 <= cA < 1/2,
    0 ≤ q ≤ 1/2,
    0 <= q0 ≤ 1/2}, {e}] // FullSimplify
```

Out[ ]= False

Analytical solution for section 6 B - {*AG*, *Ag*}

In[ ]:=
```
Remove["Global`*"];
Quit[]
```

In[ ]:=
```
At = L * (xAG[t] + xAg[t]) + (1 - L) * xAG[t]  /. xAg[t] → (1 - xAG[t]) // FullSimplify
Gt = (xAG[t] + xaG[t]) /. xaG[t] → 0
P  = q0 + q * At // FullSimplify
```

Out[ ]= L + xAG[t] - L xAG[t]

Out[ ]= xAG[t]

Out[ ]= q0 + q (L + xAG[t] - L xAG[t])

```
wAG = 1   + P * (e + (1 - e) * Gt)   - P (cG + cA)
wAg = 1   + P * ((1 - e) * Gt)       - q0 * cA

wAG - wAg // FullSimplify

 (*graph of WAG - wAg*)
Manipulate[ Plot[% /. xAG[t] -> xAG, {xAG, 0, 1}, PlotRange → {{0, 1}, {-0.31, 0.31}} ],
  {q0, 0, 1/2}, {q, 0, 1/2}, {e, 0, 1}, {cG, 0, 1/2}, {cA, 0, 1/2}, {L , 0, 1}]
```

Out[∘]= 1 - (cA + cG) (q0 + q (L + xAG[t] - L xAG[t])) + (e + (1 - e) xAG[t]) (q0 + q (L + xAG[t] - L xAG[t]))

Out[∘]= 1 - cA q0 + (1 - e) xAG[t] (q0 + q (L + xAG[t] - L xAG[t]))

Out[∘]= - cA L q - (cG - e) (L q + q0) + (cA + cG - e) (-1 + L) q xAG[t]

Out[∘]=
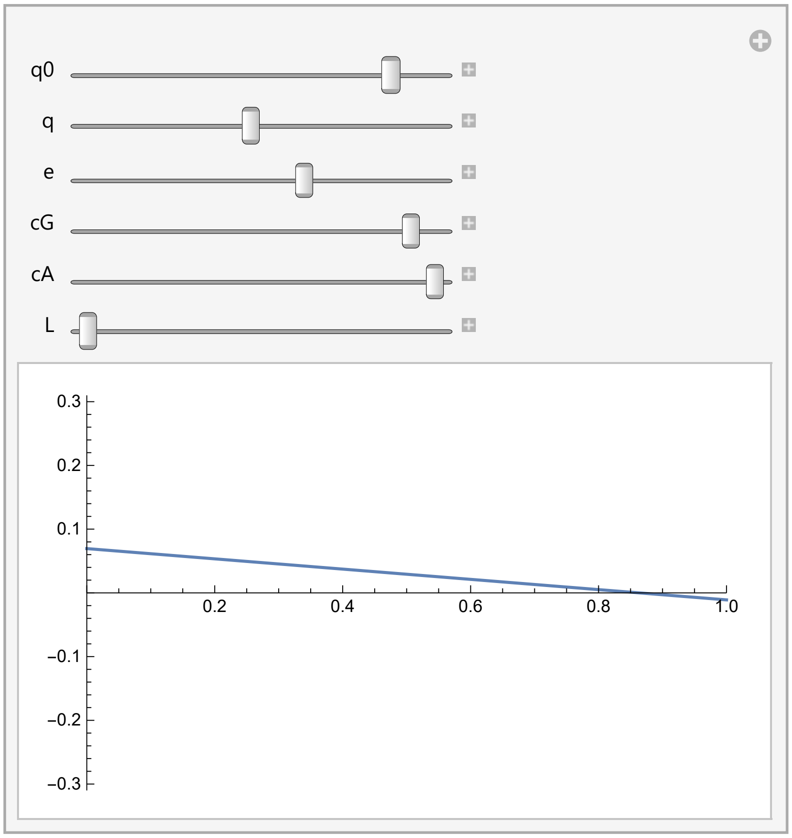

In[∘]:=
```
Diff = wAG - wAg //FullSimplify
Collect[Diff,{q}, Simplify]
```

Out[∘]= - cA L q - (cG - e) (L q + q0) + (cA + cG - e) (-1 + L) q xAG[t]

Out[∘]= (- cG + e) q0 + (cA + cG - e) q (- L + (-1 + L) xAG[t])

In[ ]:=
```
(*Mean Fitness*)
W =  wAG * xAG[t] + wAg * xAg[t] /. xAg[t] → (1 - xAG[t]) // FullSimplify
W // Expand
Manipulate[ Plot[% /. xAG[t] -> xAG, {xAG, 0, 1}, PlotRange → {{0, 1}, {0, 2.5}} ],
 {q0, 0, 1/2}, {q, 0, 1/2}, (*{e,0,1},*){cG, 0, 1/2}, {cA, 0, 1/2}, {L, 0, 1}]
```

Out[ ]= 1 - cA q0 + xAG[t] (-(-1 + cA + cG) L q + q0 - cG q0 + (-1 + cA + cG) (-1 + L) q xAG[t])

Out[ ]= 1 - cA q0 + L q xAG[t] - cA L q xAG[t] - cG L q xAG[t] + q0 xAG[t] - cG q0 xAG[t] + q xAG[t]^2 - cA q xAG[t]^2 - cG q xAG[t]^2 - L q xAG[t]^2 + cA L q xAG[t]^2 + cG L q xAG[t]^2

Out[ ]=
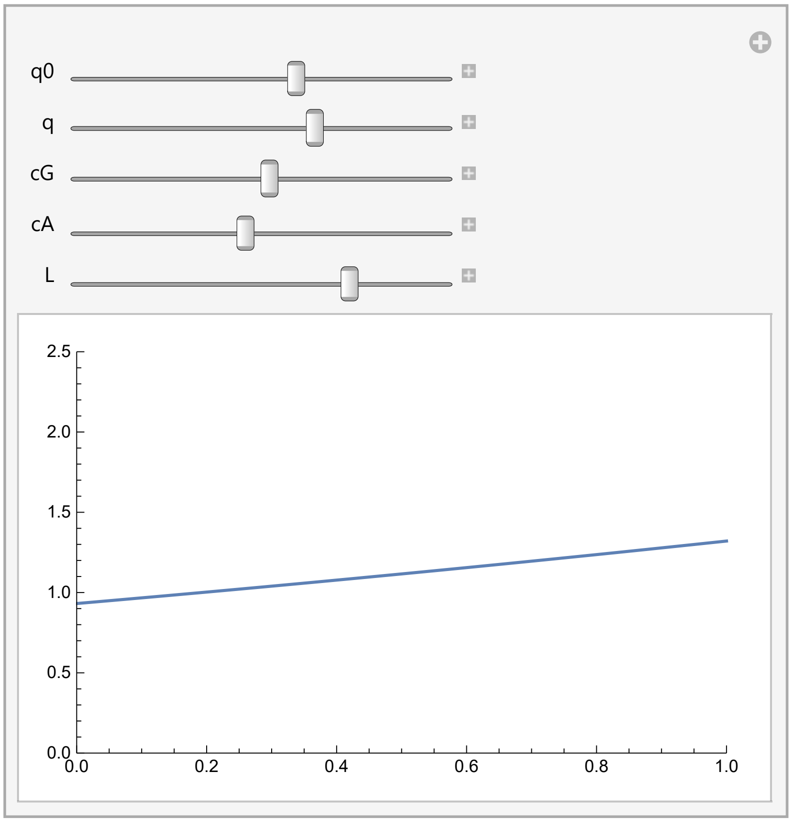

In[ ]:=
```
(*Plotting mean fitness (W) and wAG*)
W /. xAG[t] -> xAG
 wAG /. xAG[t] -> xAG
 wAg /. xAG[t] -> xAG
Manipulate[ Plot[{%%% /. xAG[t] -> xAG, %% /. xAG[t] -> xAG, % /. xAG[t] -> xAG},
   {xAG, 0, 1}, PlotRange → {{0, 1}, {0.5, 1.5}} ], {q0, 0, 1/2},
  {q, 0, 1/2}, {e, 0, 1}, {cG, 0, 1/2}, {cA, 0, 1/2}, {L, 0, 1}]
(*
legend:
   W→blue
   wAG→ yellow
    wAg→ green
*)
```

Out[ ]= $1 - cA\, q0 + xAG\left(-\left(-1 + cA + cG\right) L\, q + q0 - cG\, q0 + \left(-1 + cA + cG\right)\left(-1 + L\right) q\, xAG\right)$

Out[ ]= $1 - \left(cA + cG\right)\left(q0 + q\left(L + xAG - L\, xAG\right)\right) + \left(e + \left(1 - e\right) xAG\right)\left(q0 + q\left(L + xAG - L\, xAG\right)\right)$

Out[ ]= $1 - cA\, q0 + \left(1 - e\right) xAG\left(q0 + q\left(L + xAG - L\, xAG\right)\right)$

Out[ ]=
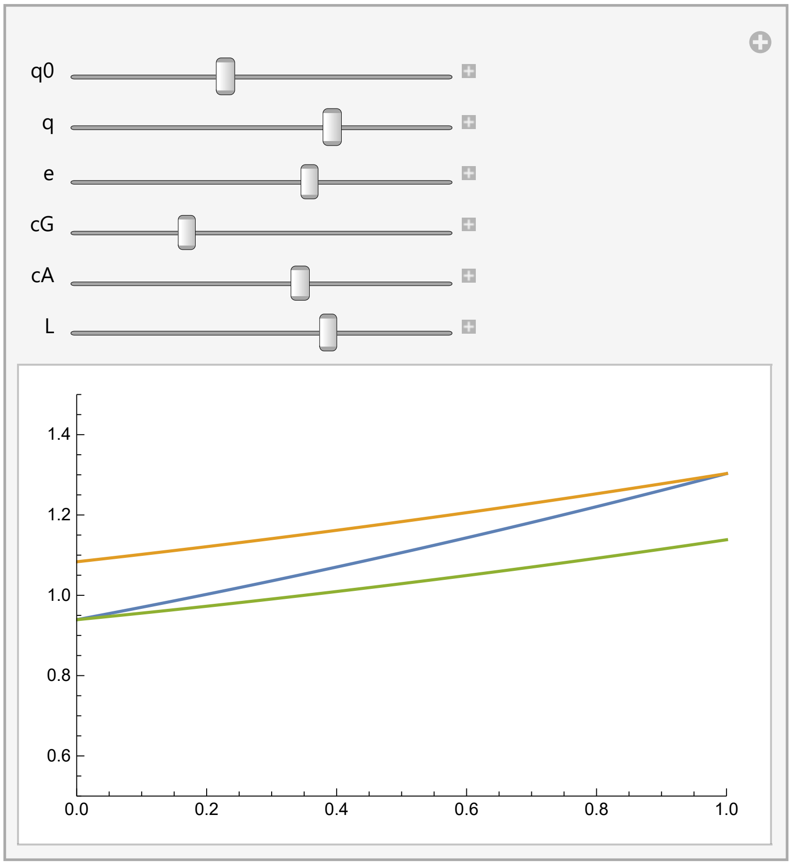

Recurrence - System of $\{x_{AG}, x_{Ag}\}$

In[∘]:=
```
xAg[t] = (1 - xAG[t]);

xAG[t + 1] = (xAG[t] * wAG / W) // FullSimplify
```

Out[∘]= (xAG[t] (1 - (cA + cG) (q0 + q (L + xAG[t] - L xAG[t])) + (e + xAG[t] - e xAG[t]) (q0 + q (L + xAG[t] - L xAG[t])))) / (1 - cA q0 + xAG[t] (- (-1 + cA + cG) L q + q0 - cG q0 + (-1 + cA + cG) (-1 + L) q xAG[t]))

Finding equilibrium

In[∘]:=
```
equil = Solve[{xAG[t + 1] - xAG[t] == 0}, {xAG[t]}];
equilibrium = equil // FullSimplify
equilibrium // MatrixForm
```

Out[∘]= $\left\{\{xAG[t] \to 0\}, \{xAG[t] \to 1\}, \left\{xAG[t] \to \frac{cA\,L\,q + (cG - e)(L\,q + q0)}{(cA + cG - e)(-1 + L)\,q}\right\}\right\}$

Out[∘]//MatrixForm=

$$\begin{pmatrix} xAG[t] \to 0 \\ xAG[t] \to 1 \\ xAG[t] \to \frac{cA\,L\,q + (cG - e)(L\,q + q0)}{(cA + cG - e)(-1 + L)\,q} \end{pmatrix}$$

Condition for existence

In[∘]:=
```
xAG1 = xAG[t] /. equilibrium[[1]]
xAG2 = xAG[t] /. equilibrium[[2]]
xAG3 = xAG[t] /. equilibrium[[3]] // FullSimplify
```

Out[∘]= 0

Out[∘]= 1

Out[∘]= $\frac{cA\,L\,q + (cG - e)(L\,q + q0)}{(cA + cG - e)(-1 + L)\,q}$

In[ ]:=
```
Reduce[{ 0 < xAG3 < 1,
   0 <= e ≤ 1,
   0 <= L ≤ 1,
   0 <= cG < 1/2,
   0 <= cA < 1/2,
   0 ≤ q ≤ 1/2,
   0 ≤ q0 ≤ 1/2}, {e}] // FullSimplify
```

Out[ ]=
$$0 < cA < \frac{1}{2} \&\& 0 < q \leq \frac{1}{2} \&\& 0 < q0 \leq \frac{1}{2} \&\& 0 \leq L < 1 \&\& \left(\left(cG == 0 \&\& \frac{cA\, L\, q}{L\, q + q0} < e < \frac{cA\, q}{q + q0}\right) \,||\, \left(0 < cG < \frac{1}{2} \&\& cG + \frac{cA\, L\, q}{L\, q + q0} < e < cG + \frac{cA\, q}{q + q0}\right)\right)$$

In[ ]:=
$$\text{FindInstance}\left[0<q\leq\frac{1}{2}\&\&0<q0\leq\frac{1}{2}\&\&0<cA<\frac{1}{2}\&\&0\leq L<1\&\&\left(0<cG<\frac{1}{2}\&\&cG+\frac{q\ cA\ L}{q0+a\ L}<e<cG+\frac{q\ cA}{q+q0}\right),\{e,q,q0,L,\ cA,cG\},R\right.$$

Out[ ]=
$$\left\{\left\{e\to\frac{69}{328},\ q\to\frac{1}{2},\ q0\to\frac{12}{203},\ L\to 0,\ cA\to\frac{10}{29},\ cG\to\frac{33}{203}\right\},\left\{e\to\frac{176}{401},\ q\to\frac{275}{603},\ q0\to\frac{9}{29},\ L\to 0,\ cA\to\frac{86}{203},\ cG\to\frac{7}{29}\right\},\left\{e\to\frac{709}{1367},\ q\to\frac{1}{2},\ q0\to\frac{1}{2},\ L\to 0,\ cA\to\frac{10}{203},\ cG\to\frac{101}{203}\right\}\right\}$$

In[ ]:=
$$\text{FindInstance}\left[0<q\leq\frac{1}{2}\&\&0<q0\leq\frac{1}{2}\&\&0<cA<\frac{1}{2}\&\&0\leq L<1\&\&\left(cG==0\&\&\frac{q\ cA\ L}{q0+q\ L}<e<\frac{q\ cA}{q+q0}\right),\{e,q,q0,L,\ cA,cG\},Reals,3\right]$$

Out[ ]=
$$\left\{\left\{e\to\frac{83}{1153},\ q\to\frac{113}{603},\ q0\to\frac{1}{2},\ L\to\frac{1}{34},\ cA\to\frac{68}{203},\ cG\to 0\right\},\left\{e\to\frac{33}{328},\ q\to\frac{1}{2},\ q0\to\frac{12}{203},\ L\to 0,\ cA\to\frac{10}{29},\ cG\to 0\right\},\left\{e\to\frac{72}{6589},\ q\to\frac{113}{603},\ q0\to\frac{32}{203},\ L\to\frac{1}{3},\ cA\to\frac{6}{203},\ cG\to 0\right\}\right\}$$

In[ ]:=
```
Reduce[{ 0 < xAG3 < 1,
   0 <= e ≤ 1,
   0 <= L ≤ 1,
   0 <= cG < 1/2,
   0 <= cA < 1/2,
   0 ≤ q ≤ 1/2,
   0 ≤ q0 ≤ 1/2}, {q0}] // FullSimplify
```

In[ ]:=
```
Reduce[{ 0 < xAG3 < 1,
   0 <= e ≤ 1,
   0 <= L ≤ 1,
   0 <= cG < 1/2,
   0 <= cA < 1/2,
   0 ≤ q ≤ 1/2,
   0 ≤ q0 ≤ 1/2}, {cA}] // FullSimplify
```

In[ ]:=
```
Reduce[{ 0 < xAG3 < 1,
   0 <= e ≤ 1,
   0 <= L ≤ 1,
   0 <= cG < 1/2,
   0 <= cA < 1/2,
   0 ≤ q ≤ 1/2,
   0 ≤ q0 ≤ 1/2}, {cG}] // FullSimplify
```

In[ ]:=
```
Reduce[{ 0 < xAG3 < 1,
   0 <= e ≤ 1,
   0 <= L ≤ 1,
   0 <= cG < 1/2,
   0 <= cA < 1/2,
   0 ≤ q ≤ 1/2,
   0 ≤ q0 ≤ 1/2}, {q}] // FullSimplify
```

In[ ]:=
```
Reduce[{ 0 < xAG3 < 1,
   0 <= e ≤ 1,
   0 <= L ≤ 1,
   0 <= cG < 1/2,
   0 <= cA < 1/2,
   0 ≤ q ≤ 1/2,
   0 ≤ q0 ≤ 1/2}, {L}] // FullSimplify
```

Stability

Step 1) -Setting Jacobian matrix

In[∘]:=
```
Jac = D[xAG[t + 1], {xAG[t]}];
J = Jac // FullSimplify
J // MatrixForm;
```

Out[∘]=
$$\Big((-1 + cA\,q0)\,(-1 + (cA + cG - e)\,(L\,q + q0)) + xAG[t]\,\big(-2\,(-1 + cA\,q0)\,((e + cA\,(-1 + L) + cG\,(-1 + L) + L - 2\,e\,L)\,q + q0 - e\,q0) + xAG[t]\,\big(-(2 + cA + cG - 3\,e)\,(-1 + L)\,q + (-1 + cA + cG)\,(-1 + e)\,L^2\,q^2 + (-cA\,(3 + cA + cG - 4\,e) + (cA^2 + cA\,(2 + cG - 3\,e) + 2\,(-1 + cG)\,(-1 + e))\,L)\,q\,q0 + (-1 + cG)\,(-1 + e)\,q0^2 + (-1 + e)\,(-1 + L)\,q\,xAG[t]\,(-2\,(-1 + cA + cG)\,L\,q - 2\,(-1 + cG)\,q0 + (-1 + cA + cG)\,(-1 + L)\,q\,xAG[t])\big)\big)\Big) \Big/ \big(1 - cA\,q0 + xAG[t]\,(-(-1 + cA + cG)\,L\,q + q0 - cG\,q0 + (-1 + cA + cG)\,(-1 + L)\,q\,xAG[t])\big)^2$$

1 JACOBIAN at {xAG[t]→0}

In[∘]:=
```
equilibrium[[1]]
J1 = J /. equilibrium[[1]] // FullSimplify
```

Out[∘]= {xAG[t] → 0}

Out[∘]=
$$\frac{-1 + (cA + cG - e)\,(L\,q + q0)}{-1 + cA\,q0}$$

In[∘]:=
```
Reduce[{ -1 < J1 < 1,
   0 <= e ≤ 1,
   0 <= L ≤ 1,
   0 <= cG < 1/2,
   0 <= cA < 1/2,
   0 ≤ q ≤ 1/2,
   0 ≤ q0 ≤ 1/2}, {e}] // FullSimplify
```

Out[∘]=
$$\Big(cA == 0\ \&\&\ 2\,cG < 1\ \&\&\ 0 \le e < cG\ \&\&\ L \le 1\ \&\&\ 2\,q \le 1\ \&\&\ ((q0 == 0\ \&\&\ L > 0\ \&\&\ q > 0)\ ||\ (q0 > 0\ \&\&\ L \ge 0\ \&\&\ q \ge 0\ \&\&\ 2\,q0 \le 1))\Big)\ ||\ \Big(0 < cA < \frac{1}{2}\ \&\&\ L \le 1\ \&\&\ e \ge 0\ \&\&\ 2\,q \le 1\ \&\&\ \Big(\Big(L > 0\ \&\&\ q > 0\ \&\&\ \Big(\Big(cG == 0\ \&\&\ q0 \ge 0\ \&\&\ e < \frac{cA\,L\,q}{L\,q + q0}\ \&\&\ 2\,q0 \le 1\Big)\ ||\ (q0 == 0\ \&\&\ cG > 0\ \&\&\ cA + cG > e\ \&\&\ 2\,cG < 1)\Big)\Big)\ ||\ (cG > 0\ \&\&\ q0 > 0\ \&\&\ (cA + cG)\,L\,q + cG\,q0 > e\,(L\,q + q0)\ \&\&\ L \ge 0\ \&\&\ q \ge 0\ \&\&\ 2\,cG < 1\ \&\&\ 2\,q0 \le 1)\Big)\Big)$$

In[∘]:=
```
Reduce[{ -1 < J1 < 1,
   0 <= e ≤ 1,
   0 <= L ≤ 1,
   0 <= cG < 1/2,
   0 <= cA < 1/2,
   0 ≤ q ≤ 1/2,
   0 ≤ q0 ≤ 1/2}, {q}] // FullSimplify
```

In[ ]:=
```
Reduce[{ -1 < J1 < 1,
   0 <= e ≤ 1,
   (0 <= L ≤ 1),
   0 <= cG < 1/2,
   0 <= cA < 1/2,
   (0 ≤ q ≤ 1/2),
   0 ≤ q0 ≤ 1/2}, {L}] // FullSimplify
```

In[ ]:=
```
Reduce[{ -1 < J1 < 1,
   0 <= e ≤ 1,
   (0 <= L ≤ 1),
   0 <= cG < 1/2,
   0 <= cA < 1/2,
   (0 ≤ q ≤ 1/2),
   0 ≤ q0 ≤ 1/2}, {cG}] // FullSimplify
```

In[ ]:=
```
Reduce[{ -1 < J1 < 1,
   0 <= e ≤ 1,
   0 <= L ≤ 1,
   0 <= cG < 1/2,
   0 <= cA < 1/2,
   0 ≤ q ≤ 1/2,
   0 ≤ q0 ≤ 1/2}, {cA}] // FullSimplify
```

In[ ]:=
```
Reduce[{ -1 < J1 < 1,
   0 <= e ≤ 1,
   0 <= L ≤ 1,
   0 <= cG < 1/2,
   0 <= cA < 1/2,
   0 ≤ q ≤ 1/2,
   0 ≤ q0 ≤ 1/2}, {q0}] // FullSimplify
```

2 JACOBIAN at {xAG[t]→1}

In[ ]:=
```
equilibrium[[2]]
J2 = J /. equilibrium[[2]] // FullSimplify
```

Out[ ]= {xAG[t] → 1}

Out[ ]= $\frac{-1 + (-1 + e)\, q + (-1 + cA + e)\, q0}{-1 + (-1 + cA + cG)\, q + (-1 + cA + cG)\, q0}$

In[ ]:=
```
Reduce[{ -1 < J2 < 1,
   0 <= e ≤ 1,
   (*0<= L≤ 1,*)
   0 <= cG < 1/2,
   0 <= cA < 1/2,
   0 ≤ q ≤ 1/2,
   0 ≤ q0 ≤ 1/2}, {e}] // FullSimplify
```

Out[ ]=
```
2 cG < 1 && cG ≥ 0 && e ≤ 1 && 2 q0 ≤ 1 &&
 ((cG < e && ((cA == 0 && q > 0 && q0 ≥ 0 && 2 q ≤ 1) || (q == 0 && q0 > 0 && 2 cA < 1 && cA ≥ 0))) ||
  (cA > 0 && q > 0 && 2 cA < 1 && cG + cA q/(q + q0) < e && q0 ≥ 0 && 2 q ≤ 1))
```

In[ ]:=
```
Reduce[{ -1 < J2 < 1,
   0 <= e ≤ 1,
   (*0<= L≤ 1,*)
   0 <= cG < 1/2,
   0 <= cA < 1/2,
   0 ≤ q ≤ 1/2,
   0 ≤ q0 ≤ 1/2}, {cG}] // FullSimplify
```

Out[ ]=
```
2 cA < 1 && cA ≥ 0 && 2 q0 ≤ 1 &&
 ((q == 0 && q0 > 0 && cG ≥ 0 && ((e > 0 && cG < e && 2 e < 1) || (2 e ≥ 1 && 2 cG < 1 && e ≤ 1))) ||
   (q > 0 && cG ≥ 0 && q0 ≥ 0 && 2 q ≤ 1 && ((2 cG < 1 && e ≤ 1 && q + 2 cA q + q0 ≤ 2 e (q + q0)) ||
       (2 e (q + q0) < q + 2 cA q + q0 && cA q + cG (q + q0) < e (q + q0)))))
```

In[ ]:=
```
Reduce[{ -1 < J2 < 1,
   0 <= e ≤ 1,
   (*0<= L≤ 1,*)
   0 <= cG < 1/2,
   0 <= cA < 1/2,
   0 ≤ q ≤ 1/2,
   0 ≤ q0 ≤ 1/2}, {cA}] // FullSimplify
```

In[ ]:=
```
Reduce[{ -1 < J2 < 1,
   0 <= e ≤ 1,
   (*0<= L≤ 1,*)
   0 <= cG < 1/2,
   0 <= cA < 1/2,
   0 ≤ q ≤ 1/2,
   0 ≤ q0 ≤ 1/2}, {q}] // FullSimplify
```

In[ ]:=
```
Reduce[{ -1 < J2 < 1,
   0 <= e ≤ 1,
   (*0<= L≤ 1,*)
   0 <= cG < 1/2,
   0 <= cA < 1/2,
   0 ≤ q ≤ 1/2,
   0 ≤ q0 ≤ 1/2}, {q0}] // FullSimplify
```

3 JACOBIAN at {xAG[t] → $\frac{\frac{q0\,(cG-e)}{aq\,(cG+cA-e)}+L}{-1+L}$}

In[ ]:=
```
(*assuming QS-independent and -dependent regulation*)
xAG3 = xAG[t] /. equilibrium[[3, 1]]
J3 = J /. equilibrium[[3]] // FullSimplify

(*assuming only QS-dependent regulation*)
xAG3q0 = xAG[t] /. equilibrium[[3, 1]] /. q0 → 0
J3q0 = J /. equilibrium[[3]] /. q0 → 0 // FullSimplify

(*assuming only QS-independent regulation is
  not posible because the equilibrium point ceases to exist*)
xAG3q = xAG[t] /. equilibrium[[3, 1]] /. q → 0
J3q = J /. equilibrium[[3]] /. q → 0 // FullSimplify
```

Out[ ]= $$\frac{cA\,L\,q+(cG-e)\,(L\,q+q0)}{(cA+cG-e)\,(-1+L)\,q}$$

Out[ ]= $$\Big((cA+cG-e)^2\,q\,\big(1+L\,(-1+(cA+cG-e)\,q)\big)+(cA+cG-e)\,\big(cA^2\,(-1+L)+cA\,(-1+2\,cG-e)\,L+(cG-e)^2\,(1+L)\big)\,q\,q0+\big(cA\,(-1+cG)+(cG-e)^2\big)\,(cG-e)\,q0^2\Big)\Big/\Big(-(cA+cG-e)^2\,(-1+L)\,q+cA\,(cA+cG-e)\,\big(-cA-cG+e+(-1+cA+cG)\,L\big)\,q\,q0+cA\,(cG-e)\,(-1+e)\,q0^2\Big)$$

Out[ ]= $$\frac{cA\,L\,q+(cG-e)\,L\,q}{(cA+cG-e)\,(-1+L)\,q}$$

Out[ ]= $$1-\frac{(cA+cG-e)\,L\,q}{-1+L}$$

Power: Infinite expression $\frac{1}{0}$ encountered.

Out[ ]= ComplexInfinity

Power: Infinite expression $\frac{1}{0}$ encountered.

Power: Infinite expression $\frac{1}{0}$ encountered.

Power: Infinite expression $\frac{1}{0}$ encountered.

General: Further output of Power::infy will be suppressed during this calculation.

Infinity: Indeterminate expression 0 ComplexInfinity encountered.

Out[ ]= Indeterminate

```
(*Under only QS-independent and -dependent regulation I aborted within 1h15m*)
Reduce[{ -1 < J3 < 1
        , 0 < xAG3 < 1
        , 0 <= e ≤ 1
        , 0 <= L < 1
   (* the equilibrium point ceases to exist if L = 1, so I changed L ≤ 1 to L < 1*)
        , 0 <= cG < 1/2
        , 0 <= cA < 1/2
        , 0 < q ≤ 1/2
   (* the equilibrium point ceases to exist if q = 0, so I changed 0 ≤ q to 0 < q*)
        , 0 < q0 ≤ 1/2 (* we already know it is not stable if q0 = 0,
   so I changed 0 <= q0 to 0 < q0*)
        }, {e}] // FullSimplify

(* aborted with 1h15min running *)
```

Out[ ]= $Aborted

In[ ]:=
```
(*Under only QS-dependent regulation, the equilibrium point is not stable*)
Reduce[{ -1 < J3q0 < 1
        , 0 < xAG3q0 < 1
        , 0 <= e ≤ 1
        , 0 <= L ≤ 1
        , 0 <= cG < 1/2
        , 0 <= cA < 1/2
        , 0 ≤ q ≤ 1/2
      (*, 0<= q0≤ 1/2*)}, {e}] // FullSimplify
```

Out[ ]= False

```
(*assuming variable QS-dependent regulation and fixed QS-independent q0→1/2*)
xAG3q0WITH05 = xAG[t] /. equilibrium[[3, 1]] /. q0 → 1/2
J3q0WITH05 = J /. equilibrium[[3]] /. q0 → 1/2 // FullSimplify


Reduce[{ -1 < xAG3q0WITH05 < 1
        , 0 < J3q0WITH05 < 1
        , 0 <= e ≤ 1
        , 0 <= L < 1
    (* the equilibrium point ceases to exist if L = 1, so I changed L ≤ 1 to L < 1*)
        , 0 <= cG < 1/2
        , 0 <= cA < 1/2
        , 0 < q ≤ 1/2
    (* the equilibrium point ceases to exist if q = 0, so I changed 0 ≤ q to 0 < q*)
        (*,0<q0≤ 1/2*) (* we already assumed q0 = 1/2*)
        }, {e}] // FullSimplify

(* aborted with 40m running *)
```

Out[]= $$\frac{\text{cA L q} + (\text{cG} - \text{e}) \left(\frac{1}{2} + \text{L q}\right)}{(\text{cA} + \text{cG} - \text{e}) (-1 + \text{L}) \text{q}}$$

Out[]= $$\Big(\big(\text{cA}(-1+\text{cG}) + (\text{cG}-\text{e})^2\big)(\text{cG}-\text{e}) + 2(\text{cA}+\text{cG}-\text{e})\big(\text{cA}^2(-1+\text{L}) + (\text{cG}-\text{e})(2+\text{cG}-\text{e}+(-2+\text{cG}-\text{e})\text{L}) + \text{cA}(2+(-3+2\,\text{cG}-\text{e})\text{L})\big)\text{q} + 4(\text{cA}+\text{cG}-\text{e})^3\text{L}\,\text{q}^2\Big) \Big/ \Big(\text{cA}(\text{cG}-\text{e})(-1+\text{e}) + 2(\text{cA}+\text{cG}-\text{e})\big(-(-2+\text{cA})(\text{cA}+\text{cG}-\text{e}) + (-2\,\text{cG}+\text{cA}(-3+\text{cA}+\text{cG})+2\,\text{e})\text{L}\big)\text{q}\Big)$$

Out[]= $Aborted

```
(*assuming variable QS-dependent regulation and fixed QS-independent q→1/2*)
xAG3qWITH05 = xAG[t] /. equilibrium[[3, 1]] /. q → 1/2
J3qWITH05 = J /. equilibrium[[3]] /. q → 1/2 // FullSimplify


Reduce[{ -1 < xAG3qWITH05 < 1
        , 0 < J3qWITH05 < 1
        , 0 <= e ≤ 1
        , 0 < L < 1
    (* the equilibrium point ceases to exist if L = 1, so I changed L ≤ 1 to L < 1*)
        , 0 <= cG < 1/2
        , 0 <= cA < 1/2
        (*,0<q≤ 1/2*)  (* we already assumed q = 1/2*)
        , 0 < q0 ≤ 1/2
    (* we already know it is not stable if q0 = 0, so I changed 0 <= q0 to 0 < q0*)
        }, {e}] // FullSimplify

(* aborted with 30m running *)
```

Out[ ]= $\dfrac{2\left(\frac{cA\,L}{2}+(cG-e)\left(\frac{L}{2}+q0\right)\right)}{(cA+cG-e)(-1+L)}$

Out[ ]= $\left((cA+cG-e)^2\,(2+(-2+cA+cG-e)\,L)+2\,(cA+cG-e)\,\left(cA^2\,(-1+L)+cA\,(-1+2\,cG-e)\,L+(cG-e)^2\,(1+L)\right)\,q0+4\,\left(cA\,(-1+cG)+(cG-e)^2\right)\,(cG-e)\,q0^2\right)\Big/\left(2\,\left(-(cA+cG-e)^2\,(-1+L)+cA\,(cA+cG-e)\,(-cA-cG+e+(-1+cA+cG)\,L)\,q0+2\,cA\,(cG-e)\,(-1+e)\,q0^2\right)\right)$

Out[ ]= $Aborted

```
(*assuming variable QS-independent and -dependent fixed, q0 →1/2, q→1/2*)
xAG3q0qWITH05 = xAG[t] /. equilibrium[[3, 1]] /. {q0 → 1/2, q → 1/2}
J3q0qWITH05 = J /. equilibrium[[3]] /. {q0 → 1/2, q → 1/2} // FullSimplify


Reduce[{ -1 < xAG3q0qWITH05 < 1
        , 0 < J3q0qWITH05 < 1
        , 0 <= e ≤ 1
        , 0 < L < 1
    (* the equilibrium point ceases to exist if L = 1, so I changed L ≤ 1 to L < 1*)
        , 0 <= cG < 1/2
        , 0 <= cA < 1/2
        (*,0<q≤ 1/2*)  (* we already assumed q = 1/2*)
        (*,0<q0≤ 1/2*)  (* we already assumed q = 1/2*)
    (* we already know it is not stable if q0 = 0, so I changed 0 <= q0 to 0 < q0*)
        }, {e}] // FullSimplify

(* Result within 15m running *)
```

Out[ ]= $\frac{2\left((cG-e)\left(\frac{1}{2}+\frac{L}{2}\right)+\frac{cA\,L}{2}\right)}{(cA+cG-e)(-1+L)}$

Out[ ]= $\Big(cA^3(-1+2L)+cA^2\left(2-cG+e+(-3+6\,cG-5e)L\right)+2(cG-e)^2\left(1+cG-e+(-1+cG-e)L\right)+cA(cG-e)\left(3-e-5(1+e)L+cG(2+6L)\right)\Big)\Big/\Big(cA(cG-e)\left(3+2e+cG(-1+L)-5L\right)+cA^3(-1+L)-2(cG-e)^2(-1+L)+cA^2\left(2(1+e)+2\,cG(-1+L)-(3+e)L\right)\Big)$

Out[ ]= cG ≥ 0 && L > 0 && 2 cA < 1 && 2 cG < 1 && L < 1 && cG + (cA + cG - e) L < e && 2 e < cA + 2 cG

```
(*assuming variable cA = 1/10*)
xAG3cAWITH01 = xAG[t] /. equilibrium[[3, 1]] /. {cA → 1/10}
J3cAWITH01 = J /. equilibrium[[3]] /. {cA → 1/10} // FullSimplify


Reduce[{ -1 < xAG3cAWITH01 < 1
       , 0 < J3cAWITH01 < 1
       , 0 <= e ≤ 1
       , 0 < L < 1
   (* the equilibrium point ceases to exist if L = 1, so I changed L ≤ 1 to L < 1*)
       , 0 <= cG < 1/2
       (*,0<=cA< 1/2 *) (* we already assumed cA = 1/10*)
      , 0 < q ≤ 1/2
   (* the equilibrium point ceases to exist if q = 0, so I changed 0 ≤ q to 0 < q*)
       , 0 < q0 ≤ 1/2 (* we already know it is not stable if q0 = 0,
   so I changed 0 <= q0 to 0 < q0*)
      }, {e}] // FullSimplify

(* aborted within 1h15m running *)
```

Out[∘]= $$\frac{\frac{L\,q}{10} + (cG - e)\,(L\,q + q0)}{\left(\frac{1}{10} + cG - e\right)\,(-1 + L)\,q}$$

Out[∘]= $$\Big((1 + 10\,cG - 10\,e)^2\,q\,(10 + L\,(-10 + q + 10\,cG\,q - 10\,e\,q)) + (1 + 10\,cG - 10\,e)\,(-1 - 9\,L - 10\,e\,L + 100\,cG^2\,(1 + L) + 100\,e^2\,(1 + L) + 20\,cG\,(L - 10\,e\,(1 + L)))\,q\,q0 + 100\,(cG - e)\,(-1 + cG + 10\,cG^2 - 20\,cG\,e + 10\,e^2)\,q0^2\Big) \Big/ \Big(100\,(cG - e)\,(-1 + e)\,q0^2 + (1 + 10\,cG - 10\,e)\,q\,(10\,(-1 + 10\,e)\,(-1 + L) + 10\,cG\,(-1 + L)\,(-10 + q0) + (-1 + 10\,e - 9\,L)\,q0)\Big)$$

Out[∘]= $Aborted

Analytical solution for section 6 C - {*AG*, *aG*}

In[∘]:=
```
Remove["Global`*"];
Quit[]
```

In[ ]:=
```
At = L * (xAG[t] + xAg[t]) + (1 - L) * xAG[t] /. xAg[t] → 0 // FullSimplify
Gt = (xAG[t] + xaG[t]) /. xaG[t] → (1 - xAG[t])
P = q0 + q * At
```

Out[ ]= xAG[t]

Out[ ]= 1

Out[ ]= q0 + q xAG[t]

```
wAG = 1 + P * (e + (1 - e) * Gt) - P (cG + cA)
waG = 1 + P * (e + (1 - e) * Gt) - P * (cG)

 wAG - waG // FullSimplify
```

Out[ ]= 1 + q0 + q xAG[t] - (cA + cG) (q0 + q xAG[t])

Out[ ]= 1 + q0 + q xAG[t] - cG (q0 + q xAG[t])

Out[ ]= - cA (q0 + q xAG[t])

In[ ]:=
```
(*Mean Fitness*)
W = wAG * xAG[t] + waG * xaG[t] /. xaG[t] → (1 - xAG[t]) // FullSimplify
W // Expand
```

Out[ ]= 1 + q0 - cG q0 - xAG[t] ((-1 + cG) q + cA q0 + cA q xAG[t])

Out[ ]= $1 + q0 - cG\, q0 + q\, xAG[t] - cG\, q\, xAG[t] - cA\, q0\, xAG[t] - cA\, q\, xAG[t]^2$

Recurrence - System of $\{x_{AG}, x_{aG}\}$

In[ ]:=
```
xaG[t] = (1 - xAG[t]);

xAG[t + 1] = (xAG[t] * wAG / W) // FullSimplify
```

Out[ ]=
$$\frac{xAG[t]\,(-1 + (-1 + cA + cG)\, q0 + (-1 + cA + cG)\, q\, xAG[t])}{-1 + (-1 + cG)\, q0 + xAG[t]\,((-1 + cG)\, q + cA\, q0 + cA\, q\, xAG[t])}$$

In[ ]:=
```
equil = Solve[{xAG[t + 1] - xAG[t] == 0}, {xAG[t]}];
equilibrium = equil // FullSimplify
equilibrium // MatrixForm
```

Out[ ]= $\{\{xAG[t] \to 0\}, \{xAG[t] \to 1\}, \{xAG[t] \to -\frac{q0}{q}\}\}$

Out[ ]//MatrixForm=
$$\begin{pmatrix} xAG[t] \to 0 \\ xAG[t] \to 1 \\ xAG[t] \to -\frac{q0}{q} \end{pmatrix}$$

Stability

Step 1) -Setting Jacobian matrix

In[ ]:=
```
Jac = D[xAG[t + 1], {{xAG[t]}}];
J = Jac // FullSimplify
J // MatrixForm;
```

Out[ ]= $\{((-1 + (-1 + cG)\, q0)\,(-1 + (-1 + cA + cG)\, q0) + q\, xAG[t]\,(2\,(-1 + cA + cG)\,(-1 + (-1 + cG)\, q0) + (cA + (-1 + cG)\,(-1 + cA + cG)\, q)\, xAG[t])) \,/\, (-1 + (-1 + cG)\, q0 + xAG[t]\,((-1 + cG)\, q + cA\, q0 + cA\, q\, xAG[t]))^2\}$

1 JACOBIAN at {xAG[t]→0}

In[ ]:=
```
equilibrium[[1]]
J1 = J /. equilibrium[[1]] // FullSimplify
```

Out[ ]= $\{xAG[t] \to 0\}$

Out[ ]= $\left\{\frac{-1 + (-1 + cA + cG)\, q0}{-1 + (-1 + cG)\, q0}\right\}$

In[ ]:=
```
Reduce[{ -1 < J1 < 1,
  (*0<= e≤ 1,
  0<= L≤ 1,*)
  0 <= cG < 1/2,
  0 <= cA < 1/2,
  (*0≤ q≤ 1/2,*)
  0 ≤ q0 ≤ 1/2}, {q0}]
```

Out[ ]= $0 \le cG < \frac{1}{2}\ \&\&\ 0 < cA < \frac{1}{2}\ \&\&\ 0 < q0 \le \frac{1}{2}$

2 JACOBIAN at {xAG[t]→1}

In[ ]:=
```
equilibrium[[2]]
J2 = J /. equilibrium[[2]] // FullSimplify
```

Out[ ]= {xAG[t] → 1}

Out[ ]= $\left\{\frac{-1+(-1+cG)\,q+(-1+cG)\,q0}{-1+(-1+cA+cG)\,q+(-1+cA+cG)\,q0}\right\}$

In[ ]:=
```
Reduce[{ -1 < J2 < 1,
   0 <= e ≤ 1,
   0 <= L ≤ 1,
   0 <= cG < 1/2,
   0 <= cA < 1/2,
   0 ≤ q ≤ 1/2,
   0 ≤ q0 ≤ 1/2}, {q}]
```

Out[ ]= False

3 JACOBIAN at {xAG[t]→ $-\frac{q0}{q}$ }

In[ ]:=
```
equilibrium[[3]]
J3 = J /. equilibrium[[3]] // FullSimplify
```

Out[ ]= $\left\{xAG[t] \to -\frac{q0}{q}\right\}$

Out[ ]= $\left\{1+\frac{cA\,q0\,(q+q0)}{q}\right\}$

In[ ]:=
```
Reduce[{ -1 < J3 < 1,
   (*0<= e≤ 1,
   0<= L≤ 1,
   0<= cG< 1/2,*)
   0 <= cA < 1/2,
   0 ≤ q ≤ 1/2,
   0 ≤ q0 ≤ 1/2}, {q}]
```

Out[ ]= False

Analytical solution for section 6 D -

{aG, Ag}

In[ ]:=
```
Remove["Global`*"];
Quit[]
```

In[ ]:=
```
At = L * (xAG[t] + xAg[t]) + (1 - L) * xAG[t] /. xAG[t] → 0 // FullSimplify
P = q0 + q * At
```

Out[ ]= L xAg[t]

Out[ ]= q0 + L q xAg[t]

```
  (*      wAG=1    +P(e -cG -cA) *)
     waG = 1  + P (e - cG)
     wAg = 1  - q0 * cA
 (*wag=1*)
```

Out[ ]= 1 + (-cG + e) (q0 + L q xAg[t])

Out[ ]= 1 - cA q0

In[ ]:=
```
 (*Mean Fitness*)
W =  waG * xaG[t] +  wAg * xAg[t]  // FullSimplify
```

Out[ ]= xaG[t] + (1 - cA q0) xAg[t] + (-cG + e) xaG[t] (q0 + L q xAg[t])

Recurrence - System of $\{x_{aG}, x_{Ag}\}$

In[ ]:=
```
xaG[t + 1] = (xaG[t] * waG / W) // FullSimplify
xAg[t + 1] = (xAg[t] * wAg / W) // FullSimplify
```

Out[ ]= $\dfrac{\text{xaG[t] (1 + (-cG + e) (q0 + L q xAg[t]))}}{\text{xaG[t] + (1 - cA q0) xAg[t] + (-cG + e) xaG[t] (q0 + L q xAg[t])}}$

Out[ ]= $\dfrac{\text{(1 - cA q0) xAg[t]}}{\text{xaG[t] + (1 - cA q0) xAg[t] + (-cG + e) xaG[t] (q0 + L q xAg[t])}}$

Finding equilibrium

In[∘]:=
```
equil = Solve[{
      xaG[t + 1] - xaG[t] == 0
    , xAg[t + 1] - xAg[t] == 0
      }, {xaG[t], xAg[t]}];
equilibrium = equil // FullSimplify;
equilibrium // MatrixForm
```

Out[∘]//MatrixForm=

$$\begin{pmatrix} xaG[t] \to 0 & xAg[t] \to 1 \\ xaG[t] \to 1 & xAg[t] \to 0 \\ xaG[t] \to 1 - \frac{(cA - cG + e)\, q0}{(cG - e)\, L\, q} & xAg[t] \to \frac{(cA - cG + e)\, q0}{(cG - e)\, L\, q} \end{pmatrix}$$

In[∘]:=
```
Dimensions[equilibrium]
Length[equilibrium]
```

Out[∘]= {3, 2}

Out[∘]= 3

In[∘]:=
```
equilibrium[[3]]
equilibrium[[3,2]]
xAg3=xAg[t]/.equilibrium[[3,2]]
```

Out[∘]= $\left\{xaG[t] \to 1 - \frac{(cA - cG + e)\, q0}{(cG - e)\, L\, q},\ xAg[t] \to \frac{(cA - cG + e)\, q0}{(cG - e)\, L\, q}\right\}$

Out[∘]= $xAg[t] \to \frac{(cA - cG + e)\, q0}{(cG - e)\, L\, q}$

Out[∘]= $\frac{(cA - cG + e)\, q0}{(cG - e)\, L\, q}$

In[ ]:=
```
Reduce[{ 0 < xAg3 < 1,
   0 <= e ≤ 1,
   0 <= L ≤ 1,
   0 <= cG < 1/2,
   0 <= cA < 1/2,
   0 ≤ q ≤ 1/2,
   0 ≤ q0 ≤ 1/2}, {e}] // FullSimplify
```

Out[ ]=
$$0 < cA < \frac{1}{2} \,\&\&\, L \le 1 \,\&\& \Bigg(\Bigg(2\,q \le 1 \,\&\& \Bigg(\Bigg(2\,q0 \le 1 \,\&\&\, q0 > 0 \,\&\& \Bigg(\Big(cA == cG \,\&\&\, e > 0 \,\&\&\, L > 0 \,\&\&\, q > 0 \,\&\&\, cA\,q0 < (cG - e)(L\,q + q0)\Big) \;||\; \Big(cA > cG \,\&\&\, cG\,q\,\big(-cA\,q0 + cG\,(L\,q + q0)\big) > 0 \,\&\&\, e \ge 0 \,\&\&\, cA < 2\,cG \,\&\&\, \frac{cA\,q0}{cG} < q + q0 \,\&\&\, e + \frac{cA\,q0}{L\,q + q0} < cG\Big)\Bigg)\Bigg) \;||\; \Bigg(cG\,q\,\big(-cA\,q0 + cG\,(L\,q + q0)\big) > 0 \,\&\&\, q0 > 0 \,\&\&\, \frac{cA\,q0}{cG} < q + q0 \,\&\&\, e + \frac{cA\,q0}{L\,q + q0} < cG \,\&\&\, e \ge 0 \,\&\& \Big(\big(cA == 2\,cG \,\&\&\, 2\,q0 < 1\big) \;||\; \big(cG > 0 \,\&\&\, 2\,cG < cA \,\&\&\, 2\,cA\,q0 < cG + 2\,cG\,q0\big)\Big)\Bigg)\Bigg)\Bigg) \;||\; \Bigg(L > 0 \,\&\&\, q > 0 \,\&\&\, q0 > 0 \,\&\&\, cA < cG \,\&\&\, 2\,cG < 1 \,\&\&\, cG < cA + e \,\&\&\, e + \frac{cA\,q0}{L\,q + q0} < cG \,\&\&\, 2\,q \le 1 \,\&\&\, 2\,q0 \le 1\Bigg)\Bigg)$$

In[ ]:=
```
FindInstance[0<q≤1/2&&0<q0≤1/2&&0<cA<1/2&&0<cG<1/2&&0≤L<1&&0≤e<1&&(0<xAg3<1),{e,q,q0,L, cA,cG},Reals,3
```

Out[ ]=
$$\Big\{\Big\{e \to \frac{50}{241},\ q \to \frac{256}{695},\ q0 \to \frac{1}{2},\ L \to \frac{2707}{2709},\ cA \to \frac{154}{487},\ cG \to \frac{437}{915}\Big\},$$
$$\Big\{e \to \frac{154}{1205},\ q \to \frac{2}{19},\ q0 \to \frac{1}{2},\ L \to \frac{16}{51},\ cA \to \frac{5}{17},\ cG \to \frac{2266}{5543}\Big\},$$
$$\Big\{e \to \frac{549}{1205},\ q \to \frac{33}{203},\ q0 \to \frac{11}{6376},\ L \to \frac{35}{114},\ cA \to \frac{15}{37},\ cG \to \frac{783}{1637}\Big\}\Big\}$$

Stability

Step 1) -Setting Jacobian matrix

In[∘]:=
```
Jac = D[{xaG[t + 1]
       , xAg[t + 1]
      }, {{xaG[t], xAg[t]}}];
J = Jac // FullSimplify;
J // MatrixForm
```

Out[∘]//MatrixForm=

$$\begin{pmatrix} \frac{(-1+cA\,q0)\,xAg[t]\,(-1+cG\,q0-e\,q0+(cG-e)\,L\,q\,xAg[t])}{((-1+cA\,q0)\,xAg[t]+xaG[t]\,(-1+cG\,q0-e\,q0+(cG-e)\,L\,q\,xAg[t]))^2} & -\frac{(-1+cA\,q0)\,(-1+cG\,q0-e\,q0)\,xaG[t]}{((-1+cA\,q0)\,xAg[t]+xaG[t]\,(-1+cG\,q0-e\,q0+(cG-e)\,L\,q\,xAg[t]))^2} \\ -\frac{(1-cA\,q0)\,xAg[t]\,(1+(-cG+e)\,(q0+L\,q\,xAg[t]))}{(xaG[t]+(1-cA\,q0)\,xAg[t]+(-cG+e)\,xaG[t]\,(q0+L\,q\,xAg[t]))^2} & \frac{(-1+cA\,q0)\,(-1+cG\,q0-e\,q0)\,xaG[t]}{((-1+cA\,q0)\,xAg[t]+xaG[t]\,(-1+cG\,q0-e\,q0+(cG-e)\,L\,q\,xAg[t]))^2} \end{pmatrix}$$

Jacobian at pure population of Ag. Stable if q≤1&&((q0==0 & cG>e & L>0 & q>0) || (q0>0 & L≥0 & q≥0 & cA q0<(cG-e) (L q+q0) & 2 q0≤1))

In[∘]:=
```
equilibrium[[1]]
J1 = J /. equilibrium[[1]] // FullSimplify;
J1 // MatrixForm

Eigenvalues[J1] // FullSimplify
Simplify[Det[J1]] // FullSimplify;
Simplify[Tr[J1]] // FullSimplify;
```

Out[∘]= {xaG[t] → 0, xAg[t] → 1}

Out[∘]//MatrixForm=

$$\begin{pmatrix} \frac{-1+(cG-e)\,(L\,q+q0)}{-1+cA\,q0} & 0 \\ \frac{1-(cG-e)\,(L\,q+q0)}{-1+cA\,q0} & 0 \end{pmatrix}$$

Out[∘]=

$$\left\{\frac{-1+(cG-e)\,(L\,q+q0)}{-1+cA\,q0},\,0\right\}$$

In[∘]:=
```
Eigenvalues[J1][[1]]
Reduce[{-1 < Eigenvalues[J1][[1]] < 1,
   0 <= e ≤ 1,
   0 <= L ≤ 1,
   0 <= cG < 1/2,
   0 <= cA < 1/2,
   0 ≤ q ≤ 1/2,
   0 ≤ q0 ≤ 1/2}, {e}] // FullSimplify
```

Out[∘]=

$$\frac{-1+cG\,L\,q-e\,L\,q+cG\,q0-e\,q0}{-1+cA\,q0}$$

Out[∘]= L ≤ 1 && cA ≥ 0 && e ≥ 0 && 2 cA < 1 && 2 cG < 1 &&
2 q ≤ 1 && ((q0 == 0 && cG > 0 && cG > e && L > 0 && q > 0) ||
(q0 > 0 && L ≥ 0 && q ≥ 0 && cA q0 < (cG - e) (L q + q0) && 2 q0 ≤ 1))

In[∘]:=
```
Eigenvalues[J1][[2]]
Reduce[{ -1 < Eigenvalues[J1][[2]] < 1,
   0 <= e ≤ 1,
   0 <= L ≤ 1,
   0 <= cG < 1/2,
   0 <= cA < 1/2,
   0 ≤ q ≤ 1/2,
   0 ≤ q0 ≤ 1/2}, {e}] // FullSimplify
```

Out[∘]= 0

Out[∘]= $0 \le L \le 1 \,\&\&\, 0 \le cG < \frac{1}{2} \,\&\&\, 0 \le cA < \frac{1}{2} \,\&\&\, 0 \le q \le \frac{1}{2} \,\&\&\, 0 \le q0 \le \frac{1}{2} \,\&\&\, 0 \le e \le 1$

In[∘]:=
```
Reduce[{ -1 < Eigenvalues[J1][[1]] < 1,
    -1 < Eigenvalues[J1][[2]] < 1,
   0 <= e ≤ 1,
   0 <= L ≤ 1,
   0 <= cG < 1/2,
   0 <= cA < 1/2,
   0 ≤ q ≤ 1/2,
   0 ≤ q0 ≤ 1/2}, {e}] // FullSimplify
```

Out[∘]= $L \le 1 \,\&\&\, cA \ge 0 \,\&\&\, e \ge 0 \,\&\&\, 2\,cA < 1 \,\&\&\, 2\,cG < 1 \,\&\&\, 2\,q \le 1 \,\&\&\, \big((q0 == 0 \,\&\&\, cG > 0 \,\&\&\, cG > e \,\&\&\, L > 0 \,\&\&\, q > 0) \,||\, (q0 > 0 \,\&\&\, L \ge 0 \,\&\&\, q \ge 0 \,\&\&\, cA\, q0 < (cG - e)\,(L\, q + q0) \,\&\&\, 2\, q0 \le 1)\big)$

Jacobian at pure population of aG. Stable if

q0 ≠ 0 ((cG == cA && e ≠ 0) || (cG < cA + e && cA < cG) || (cG < cA && cG ≠ 0 && e ≥ 0))

In[∘]:=
```
equilibrium[[2]]
J2 = J /. equilibrium[[2]] // FullSimplify;
J2 // MatrixForm

Eigenvalues[J2] // FullSimplify
Simplify[Det[J2]] // FullSimplify;
Simplify[Tr[J2]] // FullSimplify;
```

Out[∘]= {xaG[t] → 1, xAg[t] → 0}

Out[∘]//MatrixForm=
$$\begin{pmatrix} 0 & \frac{1-(-1+cA+e)\,q0}{-1+(-1+cG)\,q0} \\ 0 & \frac{-1+(-1+cA+e)\,q0}{-1+(-1+cG)\,q0} \end{pmatrix}$$

Out[∘]= $\left\{\frac{-1+(-1+cA+e)\,q0}{-1+(-1+cG)\,q0},\, 0\right\}$

In[∘]:=
```
Eigenvalues[J2][[1]]
Reduce[{ -1 < Eigenvalues[J2][[1]] < 1,
    0 <= e ≤ 1,
    0 <= L ≤ 1,
    0 <= cG < 1/2,
    0 <= cA < 1/2,
    0 ≤ q ≤ 1/2,
    0 ≤ q0 ≤ 1/2}, {e}] // FullSimplify
```

Out[∘]= $\frac{-1 - q0 + cA\, q0 + e\, q0}{-1 - q0 + cG\, q0}$

Out[∘]= $0 < q0 \le \frac{1}{2}$ && $0 \le cA < \frac{1}{2}$ && $0 \le L \le 1$ && $0 \le q \le \frac{1}{2}$ && $e \le 1$ && $((cA == cG$ && $e > 0)$ || $(cA + e > cG$ && $cA < cG$ && $2\, cG < 1)$ || $(cG < cA$ && $cG \ge 0$ && $e \ge 0))$

In[∘]:=
```
Eigenvalues[J2][[2]]
Reduce[{ -1 < Eigenvalues[J2][[2]] < 1,
    0 <= e ≤ 1,
    0 <= L ≤ 1,
    0 <= cG < 1/2,
    0 <= cA < 1/2,
    0 ≤ q ≤ 1/2,
    0 ≤ q0 ≤ 1/2}, {e}] // FullSimplify
```

Out[∘]= 0

Out[∘]= $0 \le L \le 1$ && $0 \le cG < \frac{1}{2}$ && $0 \le cA < \frac{1}{2}$ && $0 \le q \le \frac{1}{2}$ && $0 \le q0 \le \frac{1}{2}$ && $0 \le e \le 1$

Jacobian at eq. equilibrium[[3]] (coexistence between Ag and aG). Always unstable

In[∘]:=
```
equilibrium[[3]]
J3 = J /. equilibrium[[3]] // FullSimplify;
J3 // MatrixForm
```

Out[∘]= $\left\{xaG[t] \to 1 - \frac{(cA - cG + e)\, q0}{(cG - e)\, L\, q},\ xAg[t] \to \frac{(cA - cG + e)\, q0}{(cG - e)\, L\, q}\right\}$

Out[∘]//MatrixForm=

$$\begin{pmatrix} \frac{(cA-cG+e)\, q0}{(cG-e)\, L\, q} & \frac{(-1+cG\, q0-e\, q0)\,(cA\, q0-(cG-e)\,(L\, q+q0))}{(cG-e)\, L\, q\,(-1+cA\, q0)} \\ -\frac{(cA-cG+e)\, q0}{(cG-e)\, L\, q} & \frac{(-1+cG\, q0-e\, q0)\,(-cA\, q0+(cG-e)\,(L\, q+q0))}{(cG-e)\, L\, q\,(-1+cA\, q0)} \end{pmatrix}$$

In[∘]:=
```
Eigenvalues[J3] // FullSimplify
Simplify[Det[J3]] // FullSimplify;
Simplify[Tr[J3]] // FullSimplify;
```

Out[∘]= $\left\{0, \frac{(-cG+e)\,L\,q + (cG-e)^2\,L\,q\,q0 + (cA-cG+e)^2\,q0^2}{(cG-e)\,L\,q\,(-1+cA\,q0)}\right\}$

In[∘]:=
```
Eigenvalues[J3][[1]]
Reduce[{ -1 < Eigenvalues[J3][[1]] < 1,
   0 <= e ≤ 1,
   0 <= L ≤ 1,
   0 <= cG < 1/2,
   0 <= cA < 1/2,
   0 ≤ q ≤ 1/2,
   0 ≤ q0 ≤ 1/2}, {e}] // FullSimplify
```

Out[∘]= 0

Out[∘]= $0 \le L \le 1\ \&\&\ 0 \le cG < \frac{1}{2}\ \&\&\ 0 \le cA < \frac{1}{2}\ \&\&\ 0 \le q \le \frac{1}{2}\ \&\&\ 0 \le q0 \le \frac{1}{2}\ \&\&\ 0 \le e \le 1$

```
equilibrium[[3]]
equilibrium[[3,2]]
xAg3=xAg[t]/.equilibrium[[3,2]]
```

Out[∘]= $\left\{xaG[t] \to 1 - \frac{(cA-cG+e)\,q0}{(cG-e)\,L\,q},\ xAg[t] \to \frac{(cA-cG+e)\,q0}{(cG-e)\,L\,q}\right\}$

Out[∘]= $xAg[t] \to \frac{(cA-cG+e)\,q0}{(cG-e)\,L\,q}$

Out[∘]= $\frac{(cA-cG+e)\,q0}{(cG-e)\,L\,q}$

```
Reduce[{ -1 < Eigenvalues[J3][[1]] < 1,
   -1 < Eigenvalues[J3][[2]] < 1,
   0 < xAg3 < 1,
   0 <= e ≤ 1,
   0 < L ≤ 1, (*L cannot be zero*)
   0 <= cG < 1/2,
   0 <= cA < 1/2,
   0 < q ≤ 1/2, (*q cannot be zero*)
   0 ≤ q0 ≤ 1/2 (*q0 cannot be zero*)
  }, {e}] // FullSimplify
(*less than 1h*)
```

Out[∘]= False

```
Reduce[{ -1 < Eigenvalues[J3][[1]] < 1,
   -1 < Eigenvalues[J3][[2]] < 1,
   0 < xAg3 < 1,
   0 <= e ≤ 1,
   L == 1,
   0 <= cG < 1/2,
   0 <= cA < 1/2,
   0 ≤ q ≤ 1/2,
   0 ≤ q0 ≤ 1/2}, {e}] // FullSimplify
(*took 20min*)
```

Out[•]= False

Analytical solution for section 6 E - {*AG*, *aG*, *Ag*, *ag*}

In[•]:=
```
Remove["Global`*"];
Quit[]
```

In[•]:=
```
At = L * (xAG[t] + xAg[t]) + (1 - L) * xAG[t] /. xAG[t] → 0 // FullSimplify
Gt = (xAG[t] + xaG[t]) /. xAG[t] → 0
P = q0 + q * At
```

Out[•]= L xAg[t]

Out[•]= xaG[t]

Out[•]= q0 + L q xAg[t]

In[•]:=
```
wAG = 1  + P * (e + (1 - e) * Gt)   - P (cG + cA)
waG = 1  + P * (e + (1 - e) * Gt)   - P * (cG)
wAg = 1  + P * ((1 - e) * Gt)       - q0 * cA
wag = 1  + P * ((1 - e) * Gt)
```

Out[•]= 1 - (cA + cG) (q0 + L q xAg[t]) + (e + (1 - e) xaG[t]) (q0 + L q xAg[t])

Out[•]= 1 - cG (q0 + L q xAg[t]) + (e + (1 - e) xaG[t]) (q0 + L q xAg[t])

Out[•]= 1 - cA q0 + (1 - e) xaG[t] (q0 + L q xAg[t])

Out[•]= 1 + (1 - e) xaG[t] (q0 + L q xAg[t])

In[∘]:=
```
(*Mean Fitness*)
W =   wAG * xAG[t] +  waG * xaG[t] +  wAg * xAg[t] + wag * xag[t]  // FullSimplify
```

Out[∘]=
```
xag[t] (1 - (-1 + e) xaG[t] (q0 + L q xAg[t])) +
 xAg[t] (1 - cA q0 - (-1 + e) xaG[t] (q0 + L q xAg[t])) +
 xaG[t] (1 - cG (q0 + L q xAg[t]) + (e + xaG[t] - e xaG[t]) (q0 + L q xAg[t])) +
 (1 - (cA + cG) (q0 + L q xAg[t]) + (e + xaG[t] - e xaG[t]) (q0 + L q xAg[t])) xAG[t]
```

Recurrence - System of $\{x_{AG}, x_{aG}, x_{Ag}, x_{ag}\}$

Finding equilibrium

Stability

Analytical solution for section SI B - $\{aG, ag\}$

In[∘]:=
```
Remove["Global`*"];
Quit[]
```

In[∘]:=
```
At = L * (xAG[t] + xAg[t]) + (1 - L) * xAG[t]  /. {xAG[t] → 0, xAg[t] → 0} // FullSimplify
Gt = (xAG[t] + xaG[t]) /. {xAG[t] → 0, xAg[t] → 0}
P  = q0 + q * At
```

Out[∘]= 0

Out[∘]= xaG[t]

Out[∘]= q0

In[∘]:=
```
(*wAG=1    + P*(e  +(1-e)*Gt)     -P(cG  +cA)*)
waG = 1   + P * (e + (1 - e) * Gt)   - P * (cG)
(*wAg=1    + P*((1-e)*Gt)             -q0*cA   *)
wag = 1   + P * ((1 - e) * Gt)
```

Out[∘]= 1 - cG q0 + q0 (e + (1 - e) xaG[t])

Out[∘]= 1 + (1 - e) q0 xaG[t]

In[]:=
```
(*Mean Fitness*)
W = waG * xaG[t] + wag * xag[t] // FullSimplify
```

Out[]= xag[t] - (-1 + e) q0 xag[t] * xaG[t] + xaG[t] (1 - cG q0 + e q0 + (q0 - e q0) xaG[t])

Recurrence - System of $\{x_{aG}, x_{ag}\}$

In[]:=
```
xaG[t + 1] = (xaG[t] * waG / W) // FullSimplify
xag[t + 1] = (xag[t] * wag / W) // FullSimplify
```

Out[]= $$\frac{xaG[t]\ (-1 + cG\ q0 - e\ q0 + (-1 + e)\ q0\ xaG[t])}{xag[t]\ (-1 + (-1 + e)\ q0\ xaG[t]) + xaG[t]\ (-1 + cG\ q0 - e\ q0 + (-1 + e)\ q0\ xaG[t])}$$

Out[]= $$\frac{xag[t]\ (-1 + (-1 + e)\ q0\ xaG[t])}{xag[t]\ (-1 + (-1 + e)\ q0\ xaG[t]) + xaG[t]\ (-1 + cG\ q0 - e\ q0 + (-1 + e)\ q0\ xaG[t])}$$

Finding equilibrium

In[]:=
```
equil = Solve[{
      xaG[t + 1] - xaG[t] == 0
     , xag[t + 1] - xag[t] == 0
    }, {xaG[t], xag[t]}];
equilibrium = equil // FullSimplify;
equilibrium // MatrixForm
```

Out[]//MatrixForm=
$$\begin{pmatrix} xaG[t] \to 0 & xag[t] \to 1 \\ xaG[t] \to 1 & xag[t] \to 0 \end{pmatrix}$$

In[]:=
```
Dimensions[equilibrium]
Length[equilibrium]
```

Out[]= {2, 2}

Out[]= 2

Stability

Step 1) -Setting Jacobian matrix

In[•]:=
```
Jac = D[{xaG[t + 1]
       , xag[t + 1]
      }, {{xaG[t], xag[t]}}];
J = Jac // FullSimplify;
J // MatrixForm
```

Out[•]//MatrixForm=
```
( xag[t] (1-cG q0+e q0+(-1+e) q0 xaG[t] (-2+(-1+e) q0 xaG[t]))                -  xaG[t] (-1+(-1+e) q0 xaG[t]) (-1+cG q0-e q0+(-1+
  ---------------------------------------------------------------------------     --------------------------------------------------
  (xag[t] (-1+(-1+e) q0 xaG[t])+xaG[t] (-1+cG q0-e q0+(-1+e) q0 xaG[t]))^2         (xag[t] (-1+(-1+e) q0 xaG[t])+xaG[t] (-1+cG q0-e q0+(
  xag[t] (-1+cG q0-e q0+(-1+e) q0 xaG[t] (2+(q0-e q0) xaG[t]))                    xaG[t] (-1+(-1+e) q0 xaG[t]) (-1+cG q0-e q0+(-1+e
  ---------------------------------------------------------------------------     --------------------------------------------------
  (xag[t] (-1+(-1+e) q0 xaG[t])+xaG[t] (-1+cG q0-e q0+(-1+e) q0 xaG[t]))^2        (xag[t] (-1+(-1+e) q0 xaG[t])+xaG[t] (-1+cG q0-e q0+(-
```

Jacobian at eq. equilibrium[[1]], (Only ag)

In[•]:=
```
equilibrium[[1]]
J1 = J /. equilibrium[[1]] // FullSimplify;
J1 // MatrixForm

Eigenvalues[J1] // FullSimplify
Simplify[Det[J1]] // FullSimplify;
Simplify[Tr[J1]] // FullSimplify;
```

Out[•]= {xaG[t] → 0, xag[t] → 1}

Out[•]//MatrixForm=

$$\begin{pmatrix} 1 - cG\,q0 + e\,q0 & 0 \\ -1 + cG\,q0 - e\,q0 & 0 \end{pmatrix}$$

Out[•]= {1 - cG q0 + e q0, 0}

In[•]:=
```
Reduce[{ -1 < Eigenvalues[J1][[1]] < 1,
   -1 < Eigenvalues[J1][[2]] < 1,
   0 <= e ≤ 1,
   0 <= L ≤ 1,
   0 <= cG < 1/2,
   0 <= cA < 1/2,
   0 ≤ q ≤ 1/2,
   0 ≤ q0 ≤ 1/2}, {e}] // FullSimplify
```

Out[•]= $0 \leq L \leq 1 \,\&\&\, 0 \leq cA < \frac{1}{2} \,\&\&\, 0 \leq q \leq \frac{1}{2} \,\&\&\, 0 < q0 \leq \frac{1}{2} \,\&\&\, 2\,cG < 1 \,\&\&\, 0 \leq e < cG$

Jacobian at eq. equilibrium[[2]], (Only aG)

In[ ]:=
```
equilibrium[[2]]
J2 = J /. equilibrium[[2]] // FullSimplify;
J2 // MatrixForm
```

Out[ ]= {xaG[t] → 1, xag[t] → 0}

Out[ ]//MatrixForm=

$$\begin{pmatrix} 0 & \frac{1+q0-e\,q0}{-1+(-1+cG)\,q0} \\ 0 & \frac{1+q0-e\,q0}{1+q0-cG\,q0} \end{pmatrix}$$

In[ ]:=
```
Eigenvalues[J2] // FullSimplify
Simplify[Det[J2]] // FullSimplify;
Simplify[Tr[J2]] // FullSimplify;
```

Out[ ]= $\left\{\frac{1+q0-e\,q0}{1+q0-cG\,q0}, 0\right\}$

In[ ]:=
```
Reduce[{-1 < Eigenvalues[J2][[1]] < 1,
   -1 < Eigenvalues[J2][[2]] < 1,
   0 <= e ≤ 1,
   0 <= L ≤ 1,
   0 <= cG < 1/2,
   0 <= cA < 1/2,
   0 ≤ q ≤ 1/2,
   0 ≤ q0 ≤ 1/2}, {e}] // FullSimplify
```

Out[ ]= $0 \le L \le 1 \,\&\&\, 0 \le cA < \frac{1}{2} \,\&\&\, 0 \le q \le \frac{1}{2} \,\&\&\, 0 < q0 \le \frac{1}{2} \,\&\&\, 0 \le cG < \frac{1}{2} \,\&\&\, cG < e \le 1$

Analytical solution for section SI C - {*aG*, *Ag*, *ag*}

In[ ]:=
```
Remove["Global`*"];
Quit[]
```

In[∘]:=
```
At = L * (xAG[t] + xAg[t]) + (1 - L) * xAG[t] /. xAG[t] → 0 // FullSimplify
Gt = (xAG[t] + xaG[t]) /. xAG[t] → 0
P = q0 + q * At
```

Out[∘]= L xAg[t]

Out[∘]= xaG[t]

Out[∘]= q0 + L q xAg[t]

```
(*Because it is a well-mixed condition,
all strains have same access to Gt. Therefore,
(1-e)Gt can be taken out from the fitness equations*)
waG = 1  + P * (e)  - P * (cG)
wAg = 1  - q0 * cA
wag = 1
```

Out[∘]= 1 - cG (q0 + L q xAg[t]) + e (q0 + L q xAg[t])

Out[∘]= 1 - cA q0

Out[∘]= 1

In[∘]:=
```
(*Mean Fitness*)
W =  (*wAG*xAG[t]+*) waG * xaG[t] +  wAg * xAg[t] + wag * xag[t] // FullSimplify
```

Out[∘]= xag[t] + xAg[t] - cA q0 xAg[t] + xaG[t] (1 - cG q0 + e q0 + (-cG + e) L q xAg[t])

Recurrence - System of $\{x_{aG}, x_{Ag}, x_{ag}\}$

In[∘]:=
```
xaG[t + 1] = (xaG[t] * waG / W) // FullSimplify
xAg[t + 1] = (xAg[t] * wAg / W) // FullSimplify
xag[t + 1] = (xag[t] * wag / W) // FullSimplify
```

Out[∘]= $\frac{\text{xaG[t] (1 - cG q0 + e q0 + (-cG + e) L q xAg[t])}}{\text{xag[t] + xAg[t] - cA q0 xAg[t] + xaG[t] (1 - cG q0 + e q0 + (-cG + e) L q xAg[t])}}$

Out[∘]= $\frac{\text{(1 - cA q0) xAg[t]}}{\text{xag[t] + xAg[t] - cA q0 xAg[t] + xaG[t] (1 - cG q0 + e q0 + (-cG + e) L q xAg[t])}}$

Out[∘]= $\frac{\text{xag[t]}}{\text{xag[t] + xAg[t] - cA q0 xAg[t] + xaG[t] (1 - cG q0 + e q0 + (-cG + e) L q xAg[t])}}$

Finding equilibrium

In[∘]:=
```
equil = Solve[{
    xaG[t + 1] - xaG[t] == 0
    , xAg[t + 1] - xAg[t] == 0
    , xag[t + 1] - xag[t] == 0}, {xaG[t], xAg[t], xag[t]}];
equilibrium = equil // FullSimplify;
equilibrium // MatrixForm
```

Out[∘]//MatrixForm=

$$\begin{pmatrix} xaG[t] \to 0 & xAg[t] \to 0 & xag[t] \to 1 \\ xaG[t] \to 0 & xAg[t] \to 1 & xag[t] \to 0 \\ xaG[t] \to 1 & xAg[t] \to 0 & xag[t] \to 0 \\ xaG[t] \to 1 - \frac{(cA - cG + e)\, q0}{(cG - e)\, L\, q} & xAg[t] \to \frac{(cA - cG + e)\, q0}{(cG - e)\, L\, q} & xag[t] \to 0 \end{pmatrix}$$

In[∘]:=
```
Dimensions[equilibrium]
Length[equilibrium]
```

Out[∘]= {4, 3}

Out[∘]= 4

```
wAg - waG // FullSimplify
 (*graph of WAg - waG*)
Manipulate[ Plot[% /. xAg[t] -> xAg, {xAg, 0, 1}, PlotRange → {{0, 1}, {-0.31, 0.31}} ],
 {q0, 0, 1/2}, {q, 0, 1/2}, {e, 0, 1}, {cG, 0, 1/2}, {cA, 0, 1/2}, {L , 0, 1}]
```

Out[]= $-(cA - cG + e)\ q0 + (cG - e)\ L\ q\ xAg[t]$

Out[]=

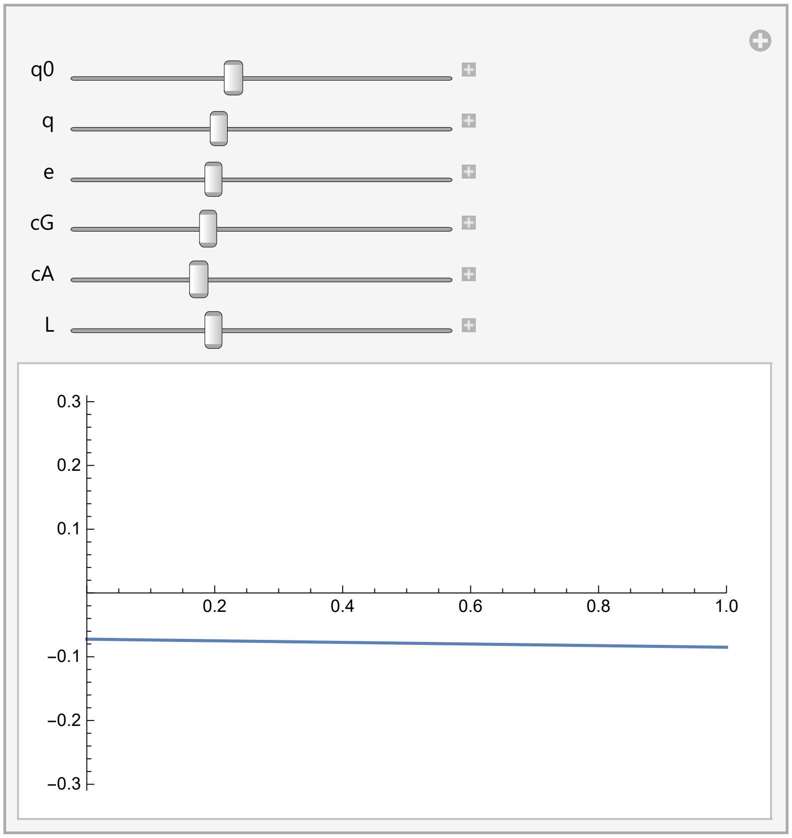

In[]:=

```
equilibrium[[4]]
equilibrium[[4,2]]
xAg4=xAg[t]/.equilibrium[[4,2]]
```

Out[]= $\left\{xaG[t] \to 1 - \frac{(cA - cG + e)\ q0}{(cG - e)\ L\ q},\ xAg[t] \to \frac{(cA - cG + e)\ q0}{(cG - e)\ L\ q},\ xag[t] \to 0\right\}$

Out[]= $xAg[t] \to \frac{(cA - cG + e)\ q0}{(cG - e)\ L\ q}$

Out[]= $\frac{(cA - cG + e)\ q0}{(cG - e)\ L\ q}$

In[ ]:=
```
Reduce[{ 0 < xAg4 < 1,
   0 <= e ≤ 1,
   0 <= L ≤ 1,
   0 <= cG < 1/2,
   0 <= cA < 1/2,
   0 ≤ q ≤ 1/2,
   0 ≤ q0 ≤ 1/2}, {e}] // FullSimplify
```

Out[ ]=
$$0 < cA < \frac{1}{2} \;\&\&\; L \le 1 \;\&\&\; \Bigg(\Bigg(2\,q \le 1 \;\&\&\; \Bigg(\Bigg(2\,q0 \le 1 \;\&\&\; q0 > 0 \;\&\&\; \Bigg(\Big(cA == cG \;\&\&\; e > 0 \;\&\&\; L > 0 \;\&\&\; q > 0 \;\&\&\; cA\,q0 < (cG - e)\,(L\,q + q0)\Big) \;||\; \Big(cA > cG \;\&\&\; cG\,q\,\big(-cA\,q0 + cG\,(L\,q + q0)\big) > 0 \;\&\&\; e \ge 0 \;\&\&\; cA < 2\,cG \;\&\&\; \frac{cA\,q0}{cG} < q + q0 \;\&\&\; e + \frac{cA\,q0}{L\,q + q0} < cG\Big)\Bigg)\Bigg) \;||\; \Bigg(cG\,q\,\big(-cA\,q0 + cG\,(L\,q + q0)\big) > 0 \;\&\&\; q0 > 0 \;\&\&\; \frac{cA\,q0}{cG} < q + q0 \;\&\&\; e + \frac{cA\,q0}{L\,q + q0} < cG \;\&\&\; e \ge 0 \;\&\&\; \Big(\big(cA == 2\,cG \;\&\&\; 2\,q0 < 1\big) \;||\; \big(cG > 0 \;\&\&\; 2\,cG < cA \;\&\&\; 2\,cA\,q0 < cG + 2\,cG\,q0\big)\Big)\Bigg)\Bigg)\Bigg) \;||\; \Bigg(L > 0 \;\&\&\; q > 0 \;\&\&\; q0 > 0 \;\&\&\; cA < cG \;\&\&\; 2\,cG < 1 \;\&\&\; cG < cA + e \;\&\&\; e + \frac{cA\,q0}{L\,q + q0} < cG \;\&\&\; 2\,q \le 1 \;\&\&\; 2\,q0 \le 1\Bigg)\Bigg)$$

In[ ]:=
$$\text{FindInstance}\Big[0<q\le\frac{1}{2}\&\&0<q0\le\frac{1}{2}\&\&0<cA<\frac{1}{2}\&\&0<cG<\frac{1}{2}\&\&0\le L<1\&\&0\le e<1\&\&(0<xAg4<1),\{e\ ,q,q0\ ,L\ ,cA,cG\},\text{Reals}$$

Out[ ]=
$$\Big\{\Big\{e \to \frac{50}{241},\ q \to \frac{256}{695},\ q0 \to \frac{1}{2},\ L \to \frac{2707}{2709},\ cA \to \frac{154}{487},\ cG \to \frac{437}{915}\Big\},$$
$$\Big\{e \to \frac{154}{1205},\ q \to \frac{2}{19},\ q0 \to \frac{1}{2},\ L \to \frac{16}{51},\ cA \to \frac{5}{17},\ cG \to \frac{2266}{5543}\Big\},$$
$$\Big\{e \to \frac{549}{1205},\ q \to \frac{33}{203},\ q0 \to \frac{11}{6376},\ L \to \frac{35}{114},\ cA \to \frac{15}{37},\ cG \to \frac{783}{1637}\Big\}\Big\}$$

Stability

Step 1) -Setting Jacobian matrix

In[•]:=
```
Jac = D[{xaG[t + 1]
    , xAg[t + 1]
    , xag[t + 1]}, {{xaG[t], xAg[t], xag[t]}}];
J = Jac // FullSimplify;
J // MatrixForm
```

Out[•]//MatrixForm=
```
( -  (-1+cG q0-e q0+(cG-e) L q xAg[t]) (xag[t]+xAg[t]-cA q0 xAg[t])                        -  ((-1+cA q0) (-1+cG q0-e q0)+(cG-e) L q xag[t]
     ----------------------------------------------------------------------                    ---------------------------------------------------
     (xag[t]+xAg[t]-cA q0 xAg[t]+xaG[t] (1-cG q0+e q0+(-cG+e) L q xAg[t]))^2                    (xag[t]+xAg[t]-cA q0 xAg[t]+xaG[t] (1-cG q0+e q0+(-c
  -  (1-cA q0) xAg[t] (1-cG q0+e q0+(-cG+e) L q xAg[t])                                      -  (-1+cA q0) (xag[t]+(1-cG q0+e q0) xaG[t
     ----------------------------------------------------------------------                    ---------------------------------------------------
     (xag[t]+xAg[t]-cA q0 xAg[t]+xaG[t] (1-cG q0+e q0+(-cG+e) L q xAg[t]))^2                    (xag[t]+xAg[t]-cA q0 xAg[t]+xaG[t] (1-cG q0+e q0+(-c
     xag[t] (-1+cG q0-e q0+(cG-e) L q xAg[t])                                                   xag[t] (-1+cA q0+(cG-e) L q xaG[t])
     ----------------------------------------------------------------------                    ---------------------------------------------------
     (xag[t]+xAg[t]-cA q0 xAg[t]+xaG[t] (1-cG q0+e q0+(-cG+e) L q xAg[t]))^2                   (xag[t]+xAg[t]-cA q0 xAg[t]+xaG[t] (1-cG q0+e q0+(-cG
```

Jacobian at eq. equilibrium[[1]] (Only ag)

In[•]:=
```
equilibrium[[1]]
J1 = J /. equilibrium[[1]] // FullSimplify;
J1 // MatrixForm

Eigenvalues[J1] // FullSimplify
Simplify[Det[J1]] // FullSimplify;
Simplify[Tr[J1]] // FullSimplify;
```

Out[•]= {xaG[t] → 0, xAg[t] → 0, xag[t] → 1}

Out[•]//MatrixForm=

$$\begin{pmatrix} 1 - cG\,q0 + e\,q0 & 0 & 0 \\ 0 & 1 - cA\,q0 & 0 \\ -1 + cG\,q0 - e\,q0 & -1 + cA\,q0 & 0 \end{pmatrix}$$

Out[•]= {0, 1 - cA q0, 1 - cG q0 + e q0}

In[•]:=
```
Eigenvalues[J1][[2]]
Eigenvalues[J1][[3]]

Reduce[{ -1 < Eigenvalues[J1][[2]] < 1,
   -1 < Eigenvalues[J1][[3]] < 1,
   0 <= e ≤ 1,
   0 <= L ≤ 1,
   0 <= cG < 1/2,
   0 <= cA < 1/2,
   0 ≤ q ≤ 1/2,
   0 ≤ q0 ≤ 1/2}, {e}] // FullSimplify
```

Out[•]= 1 - cA q0

Out[•]= 1 - cG q0 + e q0

Out[•]= $0 \le L \le 1 \;\&\&\; 0 \le q \le \frac{1}{2} \;\&\&\; 0 < q0 \le \frac{1}{2} \;\&\&\; 2\,cG < 1 \;\&\&\; 0 < cA < \frac{1}{2} \;\&\&\; 0 \le e < cG$

Jacobian at eq. equilibrium[[2]] (Only Ag)

In[ ]:=
```
equilibrium[[2]]
J2 = J /. equilibrium[[2]] // FullSimplify;
J2 // MatrixForm
```

Out[ ]= {xaG[t] → 0, xAg[t] → 1, xag[t] → 0}

Out[ ]//MatrixForm=

$$\begin{pmatrix} \frac{-1+(cG-e)\ (L\,q+q0)}{-1+cA\,q0} & 0 & 0 \\ \frac{1-(cG-e)\ (L\,q+q0)}{-1+cA\,q0} & 0 & \frac{1}{-1+cA\,q0} \\ 0 & 0 & \frac{1}{1-cA\,q0} \end{pmatrix}$$

In[ ]:=
```
Eigenvalues[J2] // FullSimplify
Simplify[Det[J2]] // FullSimplify;
Simplify[Tr[J2]] // FullSimplify;
```

Out[ ]= $\left\{\frac{1}{1-cA\,q0}, 0, \frac{-1+(cG-e)\ (L\,q+q0)}{-1+cA\,q0}\right\}$

In[ ]:=
```
Eigenvalues[J2][[1]]
Reduce[{ -1 < Eigenvalues[J2][[1]] < 1,
   -1 < Eigenvalues[J2][[2]] < 1,
   -1 < Eigenvalues[J2][[3]] < 1,
   0 <= e ≤ 1,
   0 <= L ≤ 1,
   0 <= cG < 1/2,
   0 <= cA < 1/2,
   0 ≤ q ≤ 1/2,
   0 ≤ q0 ≤ 1/2}, {e}] // FullSimplify
```

Out[ ]= $-\frac{1}{-1+cA\,q0}$

Out[ ]= False

Jacobian at eq. equilibrium[[3]] (Only aG)

In[ ]:=
```
equilibrium[[3]]
J3 = J /. equilibrium[[3]] // FullSimplify;
J3 // MatrixForm
```

Out[ ]= {xaG[t] → 1, xAg[t] → 0, xag[t] → 0}

Out[ ]//MatrixForm=

$$\begin{pmatrix} 0 & \frac{1-cA\,q0}{-1+cG\,q0-e\,q0} & \frac{1}{-1+cG\,q0-e\,q0} \\ 0 & \frac{1-cA\,q0}{1-cG\,q0+e\,q0} & 0 \\ 0 & 0 & \frac{1}{1-cG\,q0+e\,q0} \end{pmatrix}$$

In[ ]:=
```
Eigenvalues[J3] // FullSimplify
Simplify[Det[J3]] // FullSimplify;
Simplify[Tr[J3]] // FullSimplify;
```

Out[ ]= $\left\{\frac{1}{1-cG\,q0+e\,q0}, 0, \frac{1-cA\,q0}{1-cG\,q0+e\,q0}\right\}$

In[ ]:=
```
Eigenvalues[J3][[1]]
Reduce[{ -1 < Eigenvalues[J3][[1]] < 1,
   -1 < Eigenvalues[J3][[2]] < 1,
   -1 < Eigenvalues[J3][[3]] < 1,
   0 <= e ≤ 1,
   0 <= L ≤ 1,
   0 <= cG < 1/2,
   0 <= cA < 1/2,
   0 ≤ q ≤ 1/2,
   0 ≤ q0 ≤ 1/2}, {e}] // FullSimplify
```

Out[ ]= $-\frac{1}{-1+cG\,q0-e\,q0}$

Out[ ]= $0 \le L \le 1 \,\&\&\, 0 \le q \le \frac{1}{2} \,\&\&\, 0 \le cG < \frac{1}{2} \,\&\&\, 0 \le cA < \frac{1}{2} \,\&\&\, 0 < q0 \le \frac{1}{2} \,\&\&\, cG < e \le 1$

In[∘]:=
```
Eigenvalues[J3][[1]]
Reduce[{-1 < Eigenvalues[J3][[1]] < 1,
   -1 < Eigenvalues[J3][[2]] < 1,
   -1 < Eigenvalues[J3][[3]] < 1,
   0 <= e ≤ 1,
   0 <= L ≤ 1,
   0 <= cG < 1/2,
   cA == 0,
   0 ≤ q ≤ 1/2,
   0 ≤ q0 ≤ 1/2}, {e}] // FullSimplify
```

Out[∘]= $-\frac{1}{-1 + cG\, q0 - e\, q0}$

Out[∘]= $cA == 0 \,\&\&\, 0 < q0 \le \frac{1}{2} \,\&\&\, 0 \le L \le 1 \,\&\&\, 0 \le q \le \frac{1}{2} \,\&\&\, e \le 1 \,\&\&\, \left((cG == 0 \,\&\&\, e > 0) \,||\, (cG > 0 \,\&\&\, 2\, cG < 1 \,\&\&\, cG < e)\right)$

Jacobian at eq. equilibrium[[4]] (Coexistence between aG and Ag)

In[∘]:=
```
equilibrium[[4]] // FullSimplify
J4 = J /. equilibrium[[4]] // FullSimplify;
J4 // MatrixForm
```

Out[∘]= $\left\{xaG[t] \to 1 - \frac{(cA - cG + e)\, q0}{(cG - e)\, L\, q},\ xAg[t] \to \frac{(cA - cG + e)\, q0}{(cG - e)\, L\, q},\ xag[t] \to 0\right\}$

Out[∘]//MatrixForm=

$$\begin{pmatrix} \frac{(cA-cG+e)\, q0}{(cG-e)\, L\, q} & \frac{(-1+cG\, q0-e\, q0)\, (cA\, q0-(cG-e)\, (L\, q+q0))}{(cG-e)\, L\, q\, (-1+cA\, q0)} & \frac{-cA\, q0+(cG-e)\, (L\, q+q0)}{(cG-e)\, L\, q\, (-1+cA\, q0)} \\ -\frac{(cA-cG+e)\, q0}{(cG-e)\, L\, q} & \frac{(-1+cG\, q0-e\, q0)\, (-cA\, q0+(cG-e)\, (L\, q+q0))}{(cG-e)\, L\, q\, (-1+cA\, q0)} & \frac{(cA-cG+e)\, q0}{(cG-e)\, L\, q\, (-1+cA\, q0)} \\ 0 & 0 & \frac{1}{1-cA\, q0} \end{pmatrix}$$

In[∘]:=
```
xAg4 = xAg[t] /. equilibrium[[4, 2]]
Eigenvalues[J4] // FullSimplify
Simplify[Det[J4]] // FullSimplify;
Simplify[Tr[J4]] // FullSimplify;
```

Out[∘]= $\frac{(cA - cG + e)\, q0}{(cG - e)\, L\, q}$

Out[∘]= $\left\{0,\ \frac{1}{1 - cA\, q0},\ \frac{(-cG + e)\, L\, q + (cG - e)^2\, L\, q\, q0 + (cA - cG + e)^2\, q0^2}{(cG - e)\, L\, q\, (-1 + cA\, q0)}\right\}$

In[ ]:=
```
Reduce[{ -1 < Eigenvalues[J4][[2]] < 1,
   0 < xAg < 1,
   0 <= e ≤ 1,
   0 < L ≤ 1, (*cannot be zero, otherwise eq point cannot exist*)
   0 <= cG < 1/2,
   0 <= cA < 1/2,
   0 < q ≤ 1/2, (*cannot be zero, otherwise eq point cannot exist*)
   0 < q0 ≤ 1/2 (*cannot be zero, otherwise eq point cannot exist*)
  }, {e}] // FullSimplify
```

Out[ ]= False

Figure SI - Numerical simulations for the pairwise interaction between *AG* and *Ag*

```
imagemSize = 350;
scale=0.28;

xmin=0;
xmax=0.5;
ymin=0;
ymax=1;
plotrange={{xmin,xmax},{ymin,ymax}};



difText= 0.08;

wt1 = Style["Selection",14];
wt2 = Style["favors",14];
wt3 = Style["AG",14,Italic];
wtlocation = .82;
wtlocation1 = {0.1  ,wtlocation};
wtlocation2 = {0.1  ,wtlocation -difText};
wtlocation3 = {0.1  ,wtlocation -difText*2};


coexistienceLegend = Style["Coexistence",10,"DisplayFormula"];


Ag1 = Style["Selection",14, White];
Ag2 = Style["favors",14, White];
Ag3 = Style["Ag",14,Italic, White];
AgLocation = .25;
AgLocation1= {0.1 ,AgLocation};
AgLocation2= {0.1 ,AgLocation -difText};
AgLocation3= {0.1 ,AgLocation -difText*2};
```

```
At = L * (xAG[t] + xAg[t]) + (1 - L) * xAG[t]  /. xAg[t] → (1 - xAG[t]) // FullSimplify
Gt = (xAG[t] + xaG[t]) /. xaG[t] → 0
P  = q0 + q * At
```

```
Out[•]= L + xAG[t] - L xAG[t]

Out[•]= xAG[t]

Out[•]= q0 + a (L + xAG[t] - L xAG[t])
```

```
(*Fitness*)
wAG = 1  + P * (e + (1 - e) * Gt)   - P * (cG + cA)
wAg = 1  + P * ((1 - e) * Gt)       - q0 * cA

xAg[t] = (1 - xAG[t]);

 (*Mean Fitness*)
W =  wAG * xAG[t] +  wAg * xAg[t]  /. xAg[t] → (1 - xAG[t]) // FullSimplify
```

```
Out[•]= 1 - (cA + cG) (q0 + a (L + xAG[t] - L xAG[t])) + (e + (1 - e) xAG[t]) (q0 + a (L + xAG[t] - L xAG[t]))

Out[•]= 1 - cA q0 + (1 - e) xAG[t] (q0 + a (L + xAG[t] - L xAG[t]))

Out[•]= 1 - cA q0 + xAG[t] (-a (-1 + cA + cG) L + q0 - cG q0 + a (-1 + cA + cG) (-1 + L) xAG[t])
```

```
In[•]:=
xAG[t+1]=(xAG[t]*wAG / W)/.{xAG[t]→x[t],xAg[t]→1- x[t]}//FullSimplify//InputForm
xAg[t+1]=(xAg[t]*wAg / W)/.{xAG[t]→x[t],xAg[t]→ 1- x[t]}//FullSimplify//InputForm;
```

```
Out[•]//InputForm=
 (x[t]*(1 - (cA + cG)*(q0 + a*(L + x[t] - L*x[t])) + (e + x[t] - e*x[t])*(q0 + a*(L + x[t
```

AG vs Ag, $q = 0.5$, $q_0 = 0.5$; graph a55

```
In[•]:=
(*Parameter values kept constant*)
q=0.5;  q0=0.5;
L=0.1;

cG=0.3;
```

```
SeedRandom[1234] (*setting the seed*)

(*Now calculating the time series *)
Tfinal=10000;
step=0.01;



f[L_,e_,q0_,q_,cA_,cG_,x_[t]]:= (x[t]*(1 - (cG + cA)*(q0 + q*(L + x[t] - L*x[t])) + (e + x[t]

dat=Table[{cA,e,RecurrenceTable[{x[t+1]==f[L,e,q0,q,cA,cG,x[t]],x[0]==RandomReal[]},x,{t,0,Tfinal
```

In[◦]:=
```
dat // MatrixForm; (*the way that the data come out from Table[{c,e,RecurrenceTable[{x[t+1]==f[c,


parameters = Take[dat, All, All, 2]; (*only getting parameter values used to run the model:=getti
parameters// MatrixForm;


d=Dimensions[parameters];
d[[2]]; (*colecting the number of columns*)

dat[[;; , ;; , 3, -1]] // MatrixForm; (*only getting values of x[tFinal]*)
xFinal=Flatten[dat[[;; , ;; , 3, -1]]]; (*putting x[tFinal] into a single list*)
xFinal2=Partition[xFinal,1];(*first step in x[tFinal] partitioning*)
xFinal3=Partition[xFinal2,d[[2]]]; (*last step in x[tFinal] partitioning*)

dataOrg=Join[parameters,xFinal3,3];(*combining the parameters and x[tFinal] lists by at each 3 lo
dataOrg//MatrixForm;

dataOrg2=Flatten[dataOrg,1]; (*colapting the data in one list*)
dataOrg2//MatrixForm;

(*Values x[Tfinal]*)
Length[dataOrg2[[;;,3]]] (*Total number of x[Tfinal] points*)
Total[dataOrg2[[;;,3]]](*Sum of x[Tfinal],(PS.:x[t] is btw zero and 1 for all t*)
ListPlot[dataOrg2[[All,3]],PlotRange→{-0.1,1.1},PlotStyle -> PointSize[0.01]] (*Plotting all x[T
InputForm@MinMax[dataOrg2[[All,3]]]
```

Out[◦]= 5151

Out[◦]= 3167.86

Out[◦]=
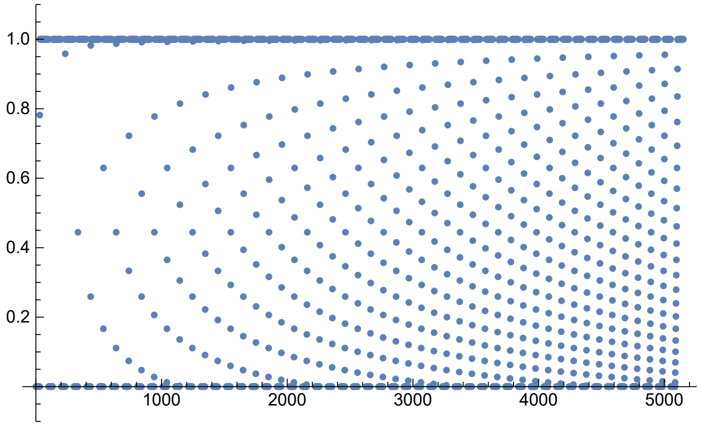

Out[◦]//InputForm=
{5.*^-324, 1.}

```
heatMap55= ListDensityPlot[dataOrg2
,FrameLabel→{Style["",15,"DisplayFormula"],Style["",15,"DisplayFormula"]}
,ColorFunction→"BrownCyanTones"
,PlotRange→{0,1}
,ColorFunctionScaling -> False
,ImageSize→imagemSize
]
```

Out[ ]=

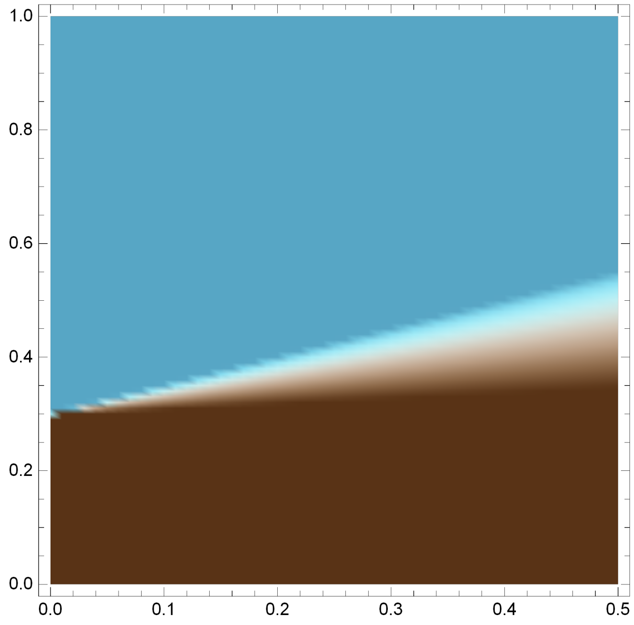

```
plotingLines=Plot[  {cG + x*(q*L/(q*L+q0)), cG + x*(q/(q+q0))},{x,0,1}  ,PlotRange→plotrange  ,
combinedPlots =Show[plotingLines, heatMap55 ,plotingLines];


a55=Graphics[
        {First[combinedPlots]
                ,Inset[wt1      , wtlocation1    ,Automatic    ,Scaled[scale]]
                ,Inset[wt2      , wtlocation2    ,Automatic    ,Scaled[scale]]
                ,Inset[wt3      , wtlocation3    ,Automatic    ,Scaled[scale]]


                ,Inset[coexistienceLegend  ,{0.45,.46}    ,Automatic    ,Scaled[.17]]
                ,Inset[coexistienceLegend  ,{0.45,.46}    ,Automatic    ,Scaled[.17]]
                ,Inset[coexistienceLegend  ,{0.45,.46}    ,Automatic    ,Scaled[.17]]

                ,Inset[Ag1    ,AgLocation1    ,Automatic    ,Scaled[scale]]
                ,Inset[Ag2    ,AgLocation2    ,Automatic    ,Scaled[scale]]
                ,Inset[Ag3    ,AgLocation3    ,Automatic    ,Scaled[scale]]
                }
        ,PlotRange→plotrange
        ,AbsoluteOptions[combinedPlots]]
```

Out[ ]=

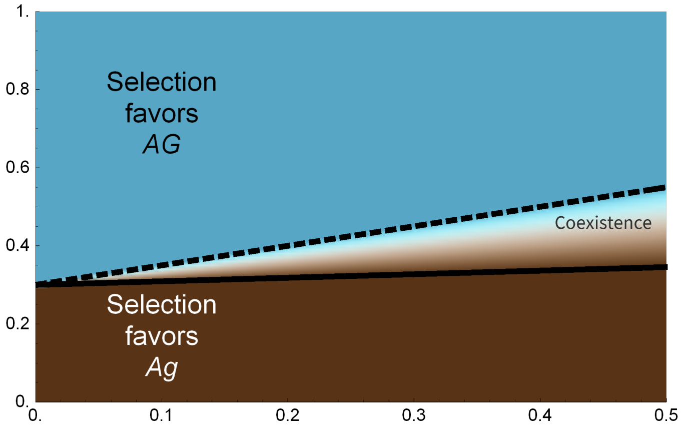

AG vs Ag, $q = 0.5$, $q_0 = 0.1$; graph a51

In[ ]:=

```
(*Parameter values not changed*)
q=0.5; q0=0.1;
L=0.1;

cG=0.3;
```

```
SeedRandom[1234] (*setting the seed*)

(*Now calculating a time series *)
Tfinal=10000;
step=0.01;




f[L_,e_,q0_,q_,cA_,cG_,x_[t]]:= (x[t]*(1 - (cG + cA)*(q0 + q*(L + x[t] - L*x[t])) + (e + x[t]

dat=Table[{cA,e,RecurrenceTable[{x[t+1]==f[L,e,q0,q,cA,cG,x[t]],x[0]==RandomReal[]},x,{t,0,Tfinal
```

In[ ]:=

```
dat // MatrixForm; (*the way that the data come out from Table[{c,e,RecurrenceTable[{x[t+1]==f[c,


parameters = Take[dat, All, All, 2]; (*only getting parameter values used to run the model:=getti
parameters// MatrixForm;


d=Dimensions[parameters];
d[[2]]; (*colecting the number of columns*)

dat[[;; , ;; , 3, -1]] // MatrixForm; (*only getting values of x[tFinal]*)
xFinal=Flatten[dat[[;; , ;; , 3, -1]]]; (*putting x[tFinal] into a single list*)
xFinal2=Partition[xFinal,1];(*first step in x[tFinal] partitioning*)
xFinal3=Partition[xFinal2,d[[2]]]; (*last step in x[tFinal] partitioning*)

dataOrg=Join[parameters,xFinal3,3];(*combining the parameters and x[tFinal] lists by at each 3 lo
dataOrg//MatrixForm;

dataOrg2=Flatten[dataOrg,1]; (*colapting the data in one list*)
dataOrg2//MatrixForm;

(*Values x[Tfinal]*)
Length[dataOrg2[[;;,3]]] (*Total number of x[Tfinal] points*)
Total[dataOrg2[[;;,3]]](*Sum of x[Tfinal],(PS.:x[t] is btw zero and 1 for all t*)
ListPlot[dataOrg2[[All,3]],PlotRange→{-0.1,1.1},PlotStyle -> PointSize[0.01]] (*Plotting all x[T
InputForm@MinMax[dataOrg2[[All,3]]]
```

Out[ ]= 5151

Out[ ]= 2713.65

Out[ ]=

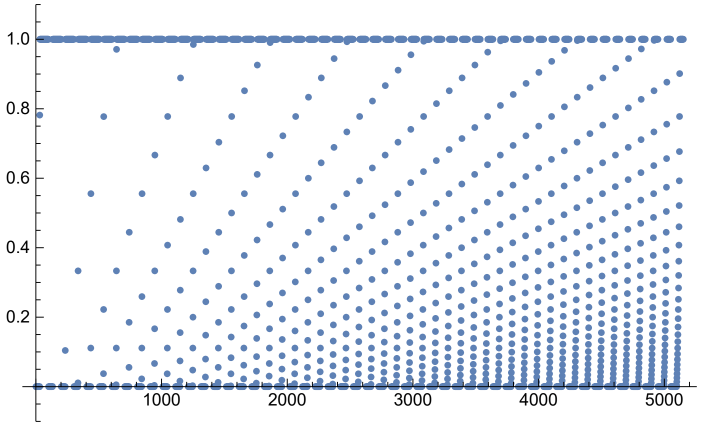

Out[ ]//InputForm=

```
{6.*^-323, 1.}
```

```
heatMap51= ListDensityPlot[dataOrg2
,FrameLabel→{Style["",15,"DisplayFormula"],Style["",15,"DisplayFormula"]}
,ColorFunction→"BrownCyanTones"
,PlotRange→{0,1}
,ColorFunctionScaling -> False
,ImageSize→imagemSize
]
```

Out[ ]=

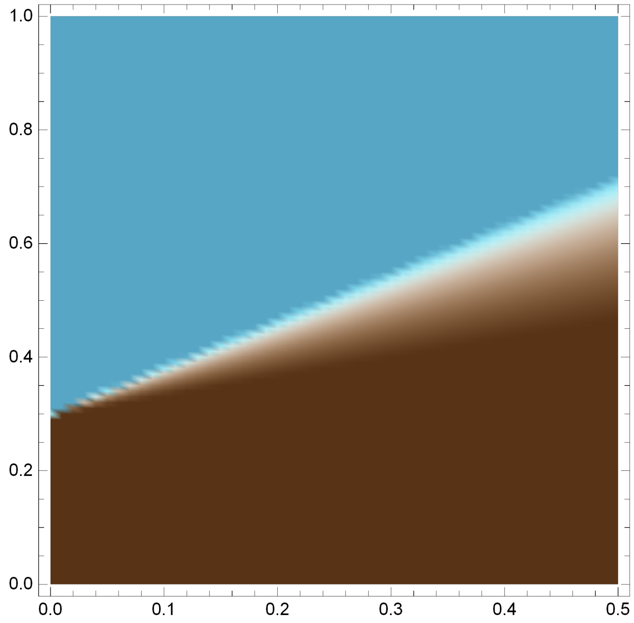

```
plotingLines=Plot[  {cG + x*(q*L/(q*L+q0)), cG + x*(q/(q+q0))},{x,0,1}  ,PlotRange→plotrange  ,
combinedPlots =Show[plotingLines, heatMap51 ,plotingLines];


a51=Graphics[
        {First[combinedPlots]
                    ,Inset[wt1      , wtlocation1    ,Automatic    ,Scaled[scale]]
                    ,Inset[wt2      , wtlocation2    ,Automatic    ,Scaled[scale]]
                    ,Inset[wt3      , wtlocation3    ,Automatic    ,Scaled[scale]]


                    ,Inset[coexistienceLegend  ,{0.45,.56}    ,Automatic    ,Scaled[.17]]
                    ,Inset[coexistienceLegend  ,{0.45,.56}    ,Automatic    ,Scaled[.17]]
                    ,Inset[coexistienceLegend  ,{0.45,.56}    ,Automatic    ,Scaled[.17]]

                    ,Inset[Ag1    ,AgLocation1    ,Automatic    ,Scaled[scale]]
                    ,Inset[Ag2    ,AgLocation2    ,Automatic    ,Scaled[scale]]
                    ,Inset[Ag3    ,AgLocation3    ,Automatic    ,Scaled[scale]]
                    }
        ,PlotRange→plotrange
        ,AbsoluteOptions[combinedPlots]]
```

Out[ ]=

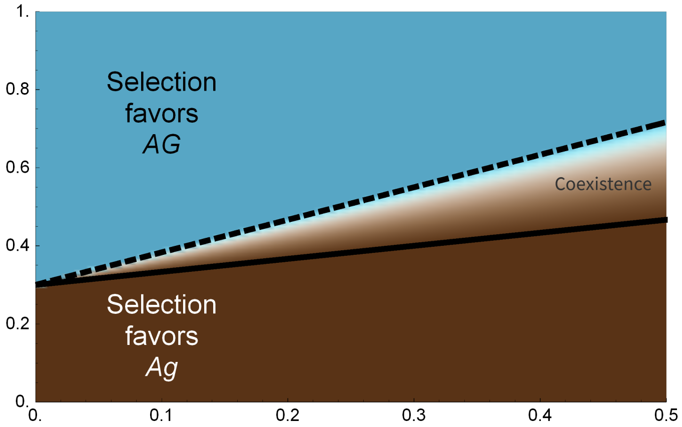

AG vs Ag, $q = 0.5$, $q_0 = 0.0$; graph a50

In[ ]:=

```
q=0.5;  q0=0.0;
L=0.1;

cG=0.3;
```

```
SeedRandom[1234] (*setting the seed*)

(*Now calculating the time series *)
Tfinal=10000;
step=0.01;




f[L_,e_,q0_,q_,cA_,cG_,x_[t]]:= (x[t]*(1 - (cG + cA)*(q0 + q*(L + x[t] - L*x[t])) + (e + x[t]

dat=Table[{cA,e,RecurrenceTable[{x[t+1]==f[L,e,q0,q,cA,cG,x[t]],x[0]==RandomReal[]},x,{t,0,Tfinal
```

In[∘]:=
```
dat // MatrixForm; (*the way that the data come out from Table[{c,e,RecurrenceTable[{x[t+1]==f[c,


parameters = Take[dat, All, All, 2]; (*only getting parameter values used to run the model:=getti
parameters// MatrixForm;


d=Dimensions[parameters];
d[[2]]; (*colecting the number of columns*)

dat[[;; , ;; , 3, -1]] // MatrixForm; (*only getting values of x[tFinal]*)
xFinal=Flatten[dat[[;; , ;; , 3, -1]]]; (*putting x[tFinal] into a single list*)
xFinal2=Partition[xFinal,1];(*first step in x[tFinal] partitioning*)
xFinal3=Partition[xFinal2,d[[2]]]; (*last step in x[tFinal] partitioning*)

dataOrg=Join[parameters,xFinal3,3];(*combining the parameters and x[tFinal] lists by at each 3 lo
dataOrg//MatrixForm;

dataOrg2=Flatten[dataOrg,1]; (*colapting the data in one list*)
dataOrg2//MatrixForm;

(*Values x[Tfinal]*)
Length[dataOrg2[[;;,3]]] (*Total number of x[Tfinal] points*)
Total[dataOrg2[[;;,3]]](*Sum of x[Tfinal],(PS.:x[t] is btw zero and 1 for all t*)
ListPlot[dataOrg2[[All,3]],PlotRange→{-0.1,1.1},PlotStyle -> PointSize[0.01]] (*Plotting all x[T
InputForm@MinMax[dataOrg2[[All,3]]]
```

Out[∘]= 5151

Out[∘]= 2320.81

Out[∘]=
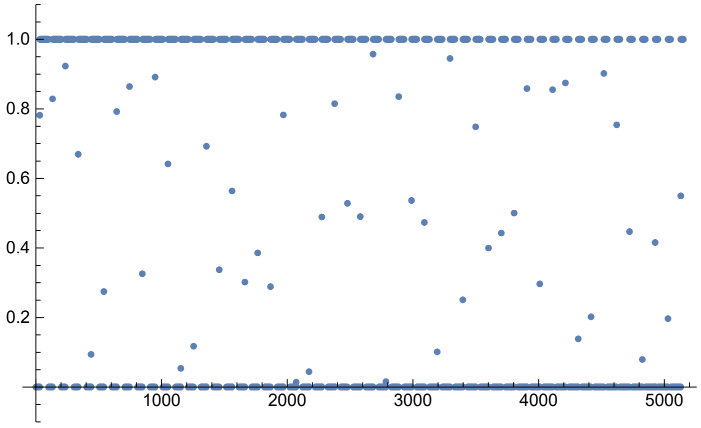

Out[∘]//InputForm=
{6.177664387018696*^-179, 1.}

```
heatMap50= ListDensityPlot[dataOrg2
,FrameLabel→{Style["",15,"DisplayFormula"],Style["",15,"DisplayFormula"]}
,ColorFunction→"BrownCyanTones"
,PlotRange→{0,1}
,ColorFunctionScaling -> False
,ImageSize→imagemSize
]
```

Out[ ]=

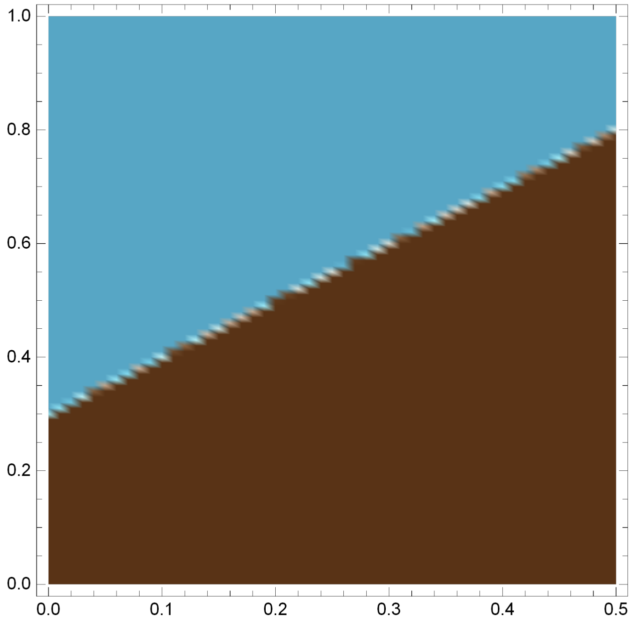

```
plotingLines=Plot[  {cG + x*(q*L/(q*L+q0)), cG + x*(q/(q+q0))},{x,0,1}  ,PlotRange→plotrange  ,
combinedPlots =Show[plotingLines, heatMap50 ,plotingLines];

a50=Graphics[
        {First[combinedPlots]
                    ,Inset[wt1      , wtlocation1    ,Automatic    ,Scaled[scale]]
                    ,Inset[wt2      , wtlocation2    ,Automatic    ,Scaled[scale]]
                    ,Inset[wt3      , wtlocation3    ,Automatic    ,Scaled[scale]]


                (*    ,Inset[coexistienceLegend  ,{0.45,.56}    ,Automatic    ,Scaled[.17]]
                    ,Inset[coexistienceLegend  ,{0.45,.56}    ,Automatic    ,Scaled[.17]]
                    ,Inset[coexistienceLegend  ,{0.45,.56}    ,Automatic    ,Scaled[.17]]*)

                    ,Inset[Ag1    ,AgLocation1    ,Automatic    ,Scaled[scale]]
                    ,Inset[Ag2    ,AgLocation2    ,Automatic    ,Scaled[scale]]
                    ,Inset[Ag3    ,AgLocation3    ,Automatic    ,Scaled[scale]]
                    }
        ,PlotRange→plotrange
        ,AbsoluteOptions[combinedPlots]]
```

Out[∘]=

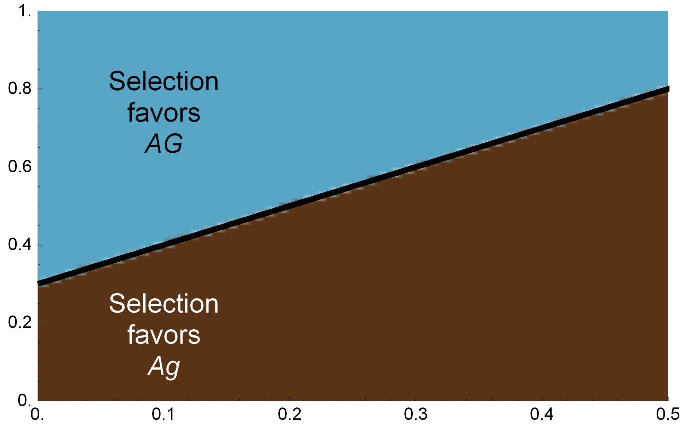

AG vs Ag, $q = 0.3$, $q_0 = 0.5$; graph a35

In[∘]:=

```
(*Parameter values not changed*)
q=0.3;  q0=0.5;
L=0.1;

cG=0.3;
```

```
SeedRandom[1234] (*setting the seed*)

(*Now calculating the time series *)
Tfinal=10000;
step=0.01;




f[L_,e_,q0_,q_,cA_,cG_,x_[t]]:= (x[t]*(1 - (cG + cA)*(q0 + q*(L + x[t] - L*x[t])) + (e + x[t]

dat=Table[{cA,e,RecurrenceTable[{x[t+1]==f[L,e,q0,q,cA,cG,x[t]],x[0]==RandomReal[]},x,{t,0,Tfinal
```

In[∘]:=

```
dat // MatrixForm; (*the way that the data come out from Table[{c,e,RecurrenceTable[{x[t+1]==f[c,


parameters = Take[dat, All, All, 2]; (*only getting parameter values used to run the model:=getti
parameters// MatrixForm;


d=Dimensions[parameters];
d[[2]]; (*colecting the number of columns*)

dat[[;; , ;; , 3, -1]] // MatrixForm; (*only getting values of x[tFinal]*)
xFinal=Flatten[dat[[;; , ;; , 3, -1]]]; (*putting x[tFinal] into a single list*)
xFinal2=Partition[xFinal,1];(*first step in x[tFinal] partitioning*)
xFinal3=Partition[xFinal2,d[[2]]]; (*last step in x[tFinal] partitioning*)

dataOrg=Join[parameters,xFinal3,3];(*combining the parameters and x[tFinal] lists by at each 3 lo
dataOrg//MatrixForm;

dataOrg2=Flatten[dataOrg,1]; (*colapting the data in one list*)
dataOrg2//MatrixForm;

(*Values x[Tfinal]*)
Length[dataOrg2[[;;,3]]] (*Total number of x[Tfinal] points*)
Total[dataOrg2[[;;,3]]](*Sum of x[Tfinal],(PS.:x[t] is btw zero and 1 for all t*)
ListPlot[dataOrg2[[All,3]],PlotRange→{-0.1,1.1},PlotStyle -> PointSize[0.01]] (*Plotting all x[T
InputForm@MinMax[dataOrg2[[All,3]]]
```

Out[∘]= 5151

Out[∘]= 3292.7

Out[∘]=

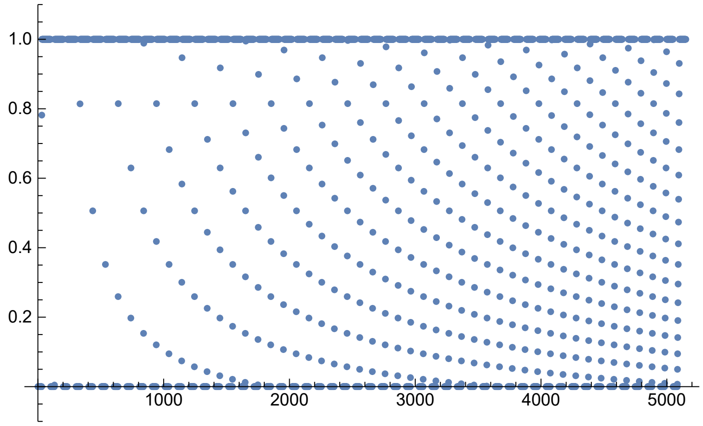

Out[∘]//InputForm=

{5.*^-324, 1.}

```
heatMap35 = ListDensityPlot[dataOrg2
,FrameLabel→{Style["",15,"DisplayFormula"],Style["",15,"DisplayFormula"]}
,ColorFunction→"BrownCyanTones"
,PlotRange→{0,1}
,ColorFunctionScaling -> False
,ImageSize→imagemSize
]
```

Out[ ]=

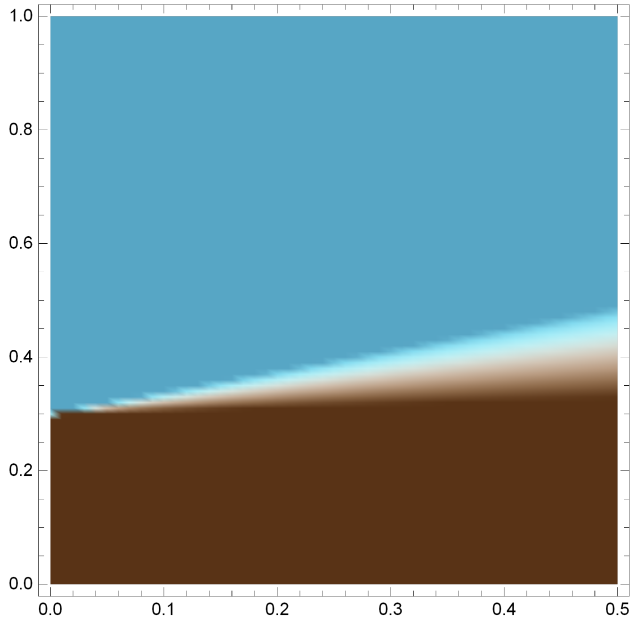

```
plotingLines=Plot[  {cG + x*(q*L/(q*L+q0)), cG + x*(q/(q+q0))},{x,0,1}  ,PlotRange→plotrange  ,
combinedPlots =Show[plotingLines, heatMap35 ,plotingLines];


a35=Graphics[
        {First[combinedPlots]
                    ,Inset[wt1      , wtlocation1    ,Automatic    ,Scaled[scale]]
                    ,Inset[wt2      , wtlocation2    ,Automatic    ,Scaled[scale]]
                    ,Inset[wt3      , wtlocation3    ,Automatic    ,Scaled[scale]]


                    ,Inset[coexistienceLegend  ,{0.45,.40}    ,Automatic    ,Scaled[.17]]
                    ,Inset[coexistienceLegend  ,{0.45,.40}    ,Automatic    ,Scaled[.17]]
                    ,Inset[coexistienceLegend  ,{0.45,.40}    ,Automatic    ,Scaled[.17]]

                    ,Inset[Ag1    ,AgLocation1    ,Automatic    ,Scaled[scale]]
                    ,Inset[Ag2    ,AgLocation2    ,Automatic    ,Scaled[scale]]
                    ,Inset[Ag3    ,AgLocation3    ,Automatic    ,Scaled[scale]]
                    }
        ,PlotRange→plotrange
        ,AbsoluteOptions[combinedPlots]]
```

Out[∘]=

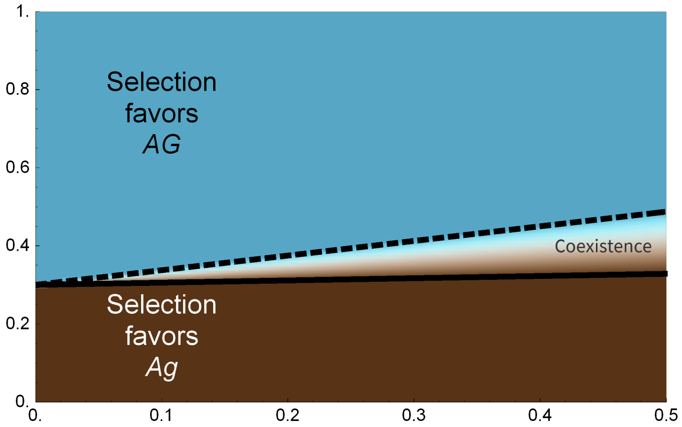

AG vs Ag, $q = 0.3$, $q_0 = 0.1$; graph a31

In[∘]:=

```
(*Parameter values not changed*)
q=0.3;  q0=0.1;
L=0.1;

cG=0.3;
```

```
SeedRandom[1234] (*setting the seed*)

(*Now calculating the time series*)
Tfinal=10000;
step=0.01;




f[L_,e_,q0_,q_,cA_,cG_,x_[t]]:= (x[t]*(1 - (cG + cA)*(q0 + q*(L + x[t] - L*x[t])) + (e + x[t]

dat=Table[{cA,e,RecurrenceTable[{x[t+1]==f[L,e,q0,q,cA,cG,x[t]],x[0]==RandomReal[]},x,{t,0,Tfinal
```

In[•]:=

```
dat // MatrixForm; (*the way that the data come out from Table[{c,e,RecurrenceTable[{x[t+1]==f[c,


parameters = Take[dat, All, All, 2]; (*only getting parameter values used to run the model:=getti
parameters// MatrixForm;


d=Dimensions[parameters];
d[[2]]; (*colecting the number of columns*)

dat[[;; , ;; , 3, -1]] // MatrixForm; (*only getting values of x[tFinal]*)
xFinal=Flatten[dat[[;; , ;; , 3, -1]]]; (*putting x[tFinal] into a single list*)
xFinal2=Partition[xFinal,1];(*first step in x[tFinal] partitioning*)
xFinal3=Partition[xFinal2,d[[2]]]; (*last step in x[tFinal] partitioning*)

dataOrg=Join[parameters,xFinal3,3];(*combining the parameters and x[tFinal] lists by at each 3 lo
dataOrg//MatrixForm;

dataOrg2=Flatten[dataOrg,1]; (*colapting the data in one list*)
dataOrg2//MatrixForm;

(*Values x[Tfinal]*)
Length[dataOrg2[[;;,3]]] (*Total number of x[Tfinal] points*)
Total[dataOrg2[[;;,3]]](*Sum of x[Tfinal],(PS.:x[t] is btw zero and 1 for all t*)
ListPlot[dataOrg2[[All,3]],PlotRange→{-0.1,1.1},PlotStyle -> PointSize[0.01]] (*Plotting all x[T
InputForm@MinMax[dataOrg2[[All,3]]]
```

Out[•]= 5151

Out[•]= 2851.54

Out[•]=

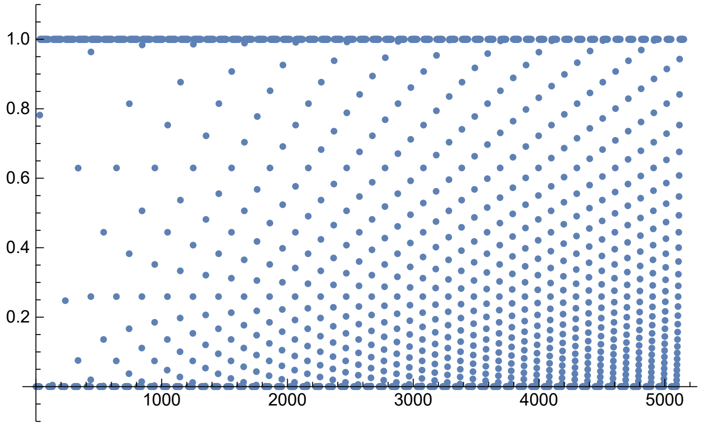

Out[•]//InputForm=

{1.9524149153615498*^-255, 1.}

```
heatMap31= ListDensityPlot[dataOrg2
,FrameLabel→{Style["",15,"DisplayFormula"],Style["",15,"DisplayFormula"]}
,ColorFunction→"BrownCyanTones"
,PlotRange→{0,1}
,ColorFunctionScaling -> False
,ImageSize→imagemSize
]
```

Out[ ]=

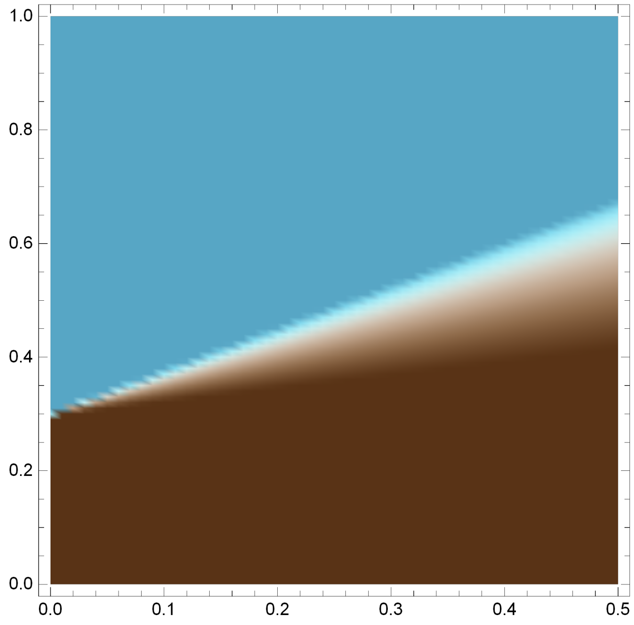

```
plotingLines=Plot[  {cG + x*(q*L/(q*L+q0)), cG + x*(q/(q+q0))},{x,0,1}  ,PlotRange→plotrange  ,
combinedPlots =Show[plotingLines, heatMap31 ,plotingLines];


a31=Graphics[
        {First[combinedPlots]
                    ,Inset[wt1      , wtlocation1    ,Automatic    ,Scaled[scale]]
                    ,Inset[wt2      , wtlocation2    ,Automatic    ,Scaled[scale]]
                    ,Inset[wt3      , wtlocation3    ,Automatic    ,Scaled[scale]]


                    ,Inset[coexistienceLegend  ,{0.45,.56}    ,Automatic    ,Scaled[.17]]
                    ,Inset[coexistienceLegend  ,{0.45,.56}    ,Automatic    ,Scaled[.17]]
                    ,Inset[coexistienceLegend  ,{0.45,.56}    ,Automatic    ,Scaled[.17]]

                    ,Inset[Ag1    ,AgLocation1    ,Automatic    ,Scaled[scale]]
                    ,Inset[Ag2    ,AgLocation2    ,Automatic    ,Scaled[scale]]
                    ,Inset[Ag3    ,AgLocation3    ,Automatic    ,Scaled[scale]]
                    }
        ,PlotRange→plotrange
        ,AbsoluteOptions[combinedPlots]]
```

Out[ ]=

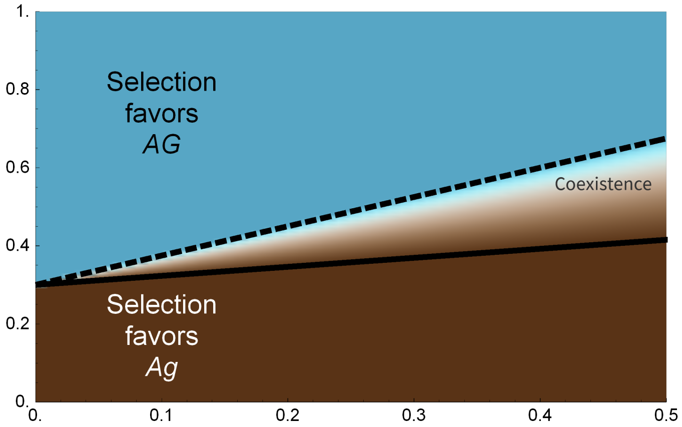

AG vs Ag, $q = 0.3$, $q_0 = 0.0$; graph a30

In[ ]:=

```
(*Parameter values not changed*)
q=0.3; q0=0.0;
L=0.1;

cG=0.3;
```

```
SeedRandom[1234] (*setting the seed*)

(*Now calculating the time series *)
Tfinal=10000;
step=0.01;




f[L_,e_,q0_,q_,cA_,cG_,x_[t]]:= (x[t]*(1 - (cG + cA)*(q0 + q*(L + x[t] - L*x[t])) + (e + x[t]

dat=Table[{cA,e,RecurrenceTable[{x[t+1]==f[L,e,q0,q,cA,cG,x[t]],x[0]==RandomReal[]},x,{t,0,Tfinal
```

In[ ]:=
```
dat // MatrixForm; (*the way that the data come out from Table[{c,e,RecurrenceTable[{x[t+1]==f[c,


parameters = Take[dat, All, All, 2]; (*only getting parameter values used to run the model:=getti
parameters// MatrixForm;


d=Dimensions[parameters];
d[[2]]; (*colecting the number of columns*)

dat[[;; , ;; , 3, -1]] // MatrixForm; (*only getting values of x[tFinal]*)
xFinal=Flatten[dat[[;; , ;; , 3, -1]]]; (*putting x[tFinal] into a single list*)
xFinal2=Partition[xFinal,1];(*first step in x[tFinal] partitioning*)
xFinal3=Partition[xFinal2,d[[2]]]; (*last step in x[tFinal] partitioning*)

dataOrg=Join[parameters,xFinal3,3];(*combining the parameters and x[tFinal] lists by at each 3 lo
dataOrg//MatrixForm;

dataOrg2=Flatten[dataOrg,1]; (*colapting the data in one list*)
dataOrg2//MatrixForm;

(*Values x[Tfinal]*)
Length[dataOrg2[[;;,3]]] (*Total number of x[Tfinal] points*)
Total[dataOrg2[[;;,3]]](*Sum of x[Tfinal],(PS.:x[t] is btw zero and 1 for all t*)
ListPlot[dataOrg2[[All,3]],PlotRange→{-0.1,1.1},PlotStyle -> PointSize[0.01]] (*Plotting all x[T
InputForm@MinMax[dataOrg2[[All,3]]]
```

Out[ ]= 5151

Out[ ]= 2320.83

Out[ ]=
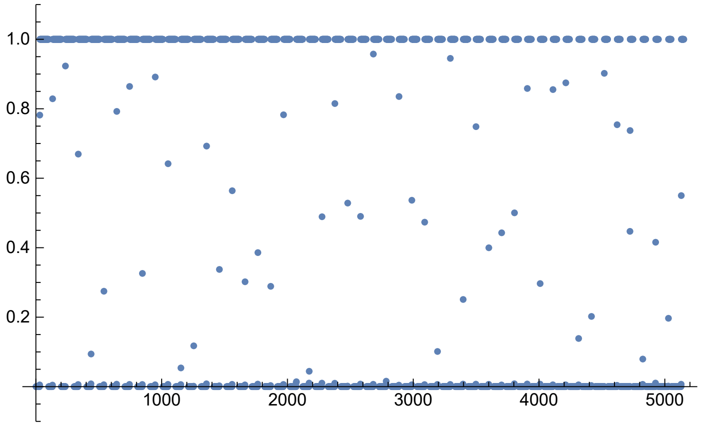

Out[ ]//InputForm=
{3.837737434060589*^-107, 1.}

```
heatMap30= ListDensityPlot[dataOrg2
,FrameLabel→{Style["",15,"DisplayFormula"],Style["",15,"DisplayFormula"]}
,ColorFunction→"BrownCyanTones"
,PlotRange→{0,1}
,ColorFunctionScaling -> False
,ImageSize→imagemSize
]
```

Out[]=

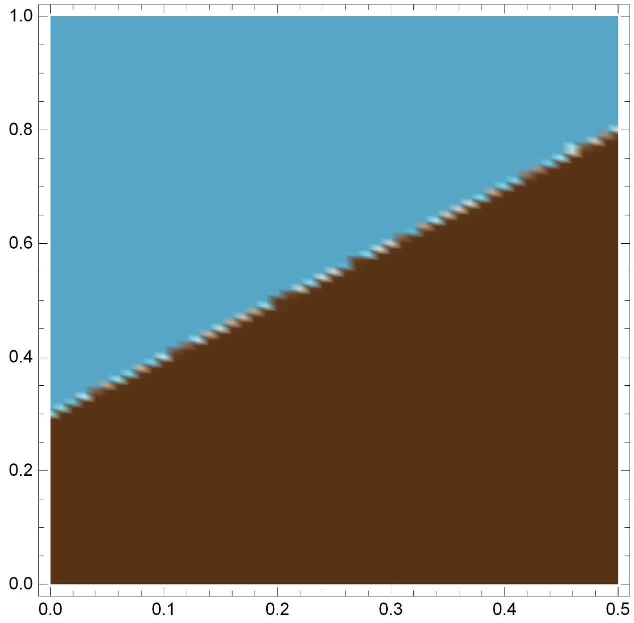

```
plotingLines=Plot[  {cG + x*(q*L/(q*L+q0)), cG + x*(q/(q+q0))},{x,0,1}  ,PlotRange→plotrange  ,
combinedPlots =Show[plotingLines, heatMap30 ,plotingLines];


a30=Graphics[
        {First[combinedPlots]
                    ,Inset[wt1      , wtlocation1    ,Automatic    ,Scaled[scale]]
                    ,Inset[wt2      , wtlocation2    ,Automatic    ,Scaled[scale]]
                    ,Inset[wt3      , wtlocation3    ,Automatic    ,Scaled[scale]]


                    (*,Inset[coexistienceLegend  ,{0.45,.56}    ,Automatic    ,Scaled[.17]]
                    ,Inset[coexistienceLegend  ,{0.45,.56}    ,Automatic    ,Scaled[.17]]
                    ,Inset[coexistienceLegend  ,{0.45,.56}    ,Automatic    ,Scaled[.17]]*)

                    ,Inset[Ag1    ,AgLocation1    ,Automatic    ,Scaled[scale]]
                    ,Inset[Ag2    ,AgLocation2    ,Automatic    ,Scaled[scale]]
                    ,Inset[Ag3    ,AgLocation3    ,Automatic    ,Scaled[scale]]
                    }
        ,PlotRange→plotrange
        ,AbsoluteOptions[combinedPlots]]
```

Out[ ]=

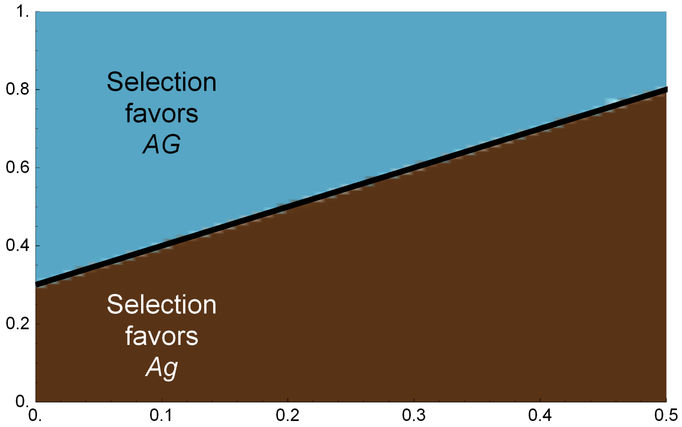

AG vs Ag, $q = 0.0$, $q_0 = 0.5$; graph a05

In[ ]:=

```
(*Parameter values not changed*)
q=0.0; q0=0.5;
L=0.1;

cG=0.3;
```

```
SeedRandom[1234] (*setting the seed*)

(*Now calculating the time series  *)
Tfinal=10000;
step=0.01;




f[L_,e_,q0_,q_,cA_,cG_,x_[t]]:= (x[t]*(1 - (cG + cA)*(q0 + q*(L + x[t] - L*x[t])) + (e + x[t]

dat=Table[{cA,e,RecurrenceTable[{x[t+1]==f[L,e,q0,q,cA,cG,x[t]],x[0]==RandomReal[]},x,{t,0,Tfinal
```

In[•]:=
```
dat // MatrixForm; (*the way that the data come out from Table[{c,e,RecurrenceTable[{x[t+1]==f[c,

parameters = Take[dat, All, All, 2]; (*only getting parameter values used to run the model:=getti
parameters// MatrixForm;


d=Dimensions[parameters];
d[[2]]; (*colecting the number of columns*)

dat[[;; , ;; , 3, -1]] // MatrixForm; (*only getting values of x[tFinal]*)
xFinal=Flatten[dat[[;; , ;; , 3, -1]]]; (*putting x[tFinal] into a single list*)
xFinal2=Partition[xFinal,1];(*first step in x[tFinal] partitioning*)
xFinal3=Partition[xFinal2,d[[2]]]; (*last step in x[tFinal] partitioning*)

dataOrg=Join[parameters,xFinal3,3];(*combining the parameters and x[tFinal] lists by at each 3 lo
dataOrg//MatrixForm;

dataOrg2=Flatten[dataOrg,1]; (*colapting the data in one list*)
dataOrg2//MatrixForm;

(*Values x[Tfinal]*)
Length[dataOrg2[[;;,3]]] (*Total number of x[Tfinal] points*)
Total[dataOrg2[[;;,3]]](*Sum of x[Tfinal],(PS.:x[t] is btw zero and 1 for all t*)
ListPlot[dataOrg2[[All,3]],PlotRange→{-0.1,1.1},PlotStyle -> PointSize[0.01]] (*Plotting all x[T
InputForm@MinMax[dataOrg2[[All,3]]]
```

Out[•]= 5151

Out[•]= 3597.15

Out[•]=

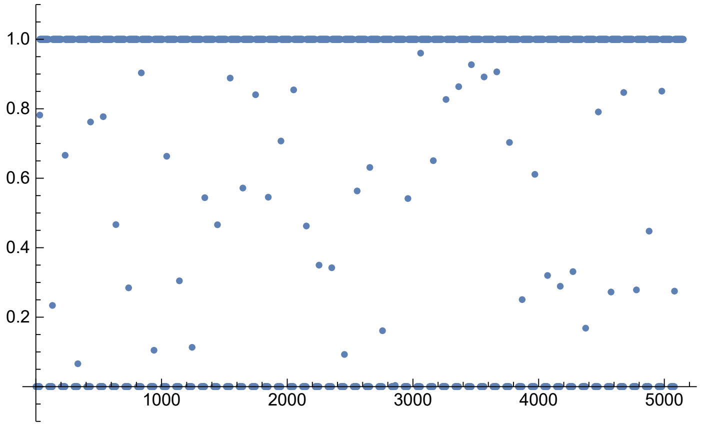

Out[•]//InputForm=
{5.*^-324, 1.}

```
heatMap05 = ListDensityPlot[dataOrg2
,FrameLabel→{Style["",15,"DisplayFormula"],Style["",15,"DisplayFormula"]}
,ColorFunction→"BrownCyanTones"
,PlotRange→{0,1}
,ColorFunctionScaling -> False
,ImageSize→imagemSize
]
```

Out[ ]=

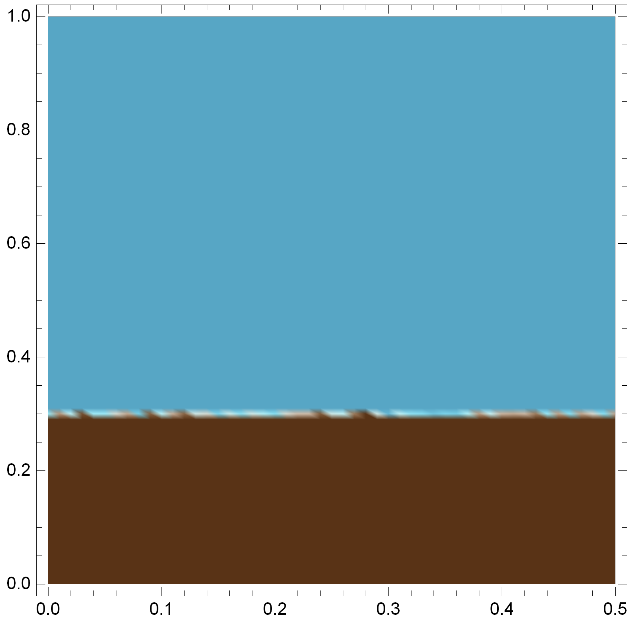

```
plotingLines=Plot[  {cG + x*(q*L/(q*L+q0)), cG + x*(q/(q+q0))},{x,0,1}  ,PlotRange→plotrange  ,
combinedPlots =Show[plotingLines, heatMap05 ,plotingLines];


a05=Graphics[
        {First[combinedPlots]
                    ,Inset[wt1     , wtlocation1    ,Automatic    ,Scaled[scale]]
                    ,Inset[wt2     , wtlocation2    ,Automatic    ,Scaled[scale]]
                    ,Inset[wt3     , wtlocation3    ,Automatic    ,Scaled[scale]]


                    (*,Inset[coexistienceLegend  ,{0.45,.56}    ,Automatic    ,Scaled[.17]]
                    ,Inset[coexistienceLegend  ,{0.45,.56}    ,Automatic    ,Scaled[.17]]
                    ,Inset[coexistienceLegend  ,{0.45,.56}    ,Automatic    ,Scaled[.17]]*)

                    ,Inset[Ag1    ,AgLocation1    ,Automatic    ,Scaled[scale]]
                    ,Inset[Ag2    ,AgLocation2    ,Automatic    ,Scaled[scale]]
                    ,Inset[Ag3    ,AgLocation3    ,Automatic    ,Scaled[scale]]
                    }
        ,PlotRange→plotrange
        ,AbsoluteOptions[combinedPlots]]
```

Out[ ]=

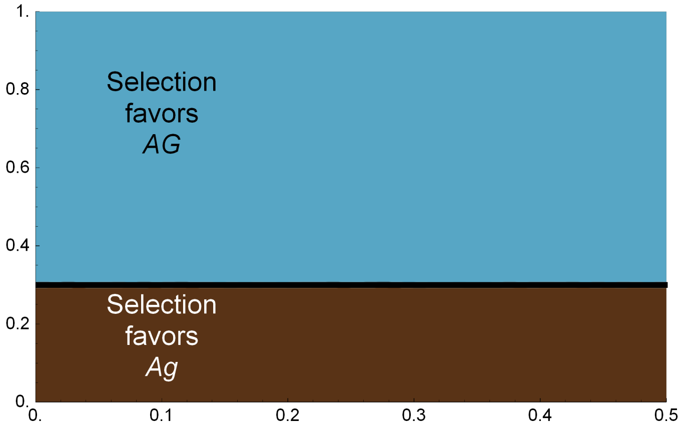

AG vs Ag, $q = 0.0$, $q_0 = 0.1$; graph a03

In[ ]:=

```
(*Parameter values not changed*)
q=0.0;  q0=0.3;
L=0.1;

cG=0.3;
```

```
SeedRandom[1234] (*setting the seed*)

(*Now calculating the time series *)
Tfinal=10000;
step=0.01;




f[L_,e_,q0_,q_,cA_,cG_,x_[t]]:= (x[t]*(1 - (cG + cA)*(q0 + q*(L + x[t] - L*x[t])) + (e + x[t]

dat=Table[{cA,e,RecurrenceTable[{x[t+1]==f[L,e,q0,q,cA,cG,x[t]],x[0]==RandomReal[]},x,{t,0,Tfinal
```

In[◦]:=
```
dat // MatrixForm; (*the way that the data come out from Table[{c,e,RecurrenceTable[{x[t+1]==f[c,


parameters = Take[dat, All, All, 2]; (*only getting parameter values used to run the model:=getti
parameters// MatrixForm;


d=Dimensions[parameters];
d[[2]]; (*colecting the number of columns*)

dat[[;; , ;; , 3, -1]] // MatrixForm; (*only getting values of x[tFinal]*)
xFinal=Flatten[dat[[;; , ;; , 3, -1]]]; (*putting x[tFinal] into a single list*)
xFinal2=Partition[xFinal,1];(*first step in x[tFinal] partitioning*)
xFinal3=Partition[xFinal2,d[[2]]]; (*last step in x[tFinal] partitioning*)

dataOrg=Join[parameters,xFinal3,3];(*combining the parameters and x[tFinal] lists by at each 3 lo
dataOrg//MatrixForm;

dataOrg2=Flatten[dataOrg,1]; (*colapting the data in one list*)
dataOrg2//MatrixForm;

(*Values x[Tfinal]*)
Length[dataOrg2[[;;,3]]] (*Total number of x[Tfinal] points*)
Total[dataOrg2[[;;,3]]](*Sum of x[Tfinal],(PS.:x[t] is btw zero and 1 for all t*)
ListPlot[dataOrg2[[All,3]],PlotRange→{-0.1,1.1},PlotStyle -> PointSize[0.01]] (*Plotting all x[T
InputForm@MinMax[dataOrg2[[All,3]]]
```

Out[◦]= 5151

Out[◦]= 3597.15

Out[◦]=
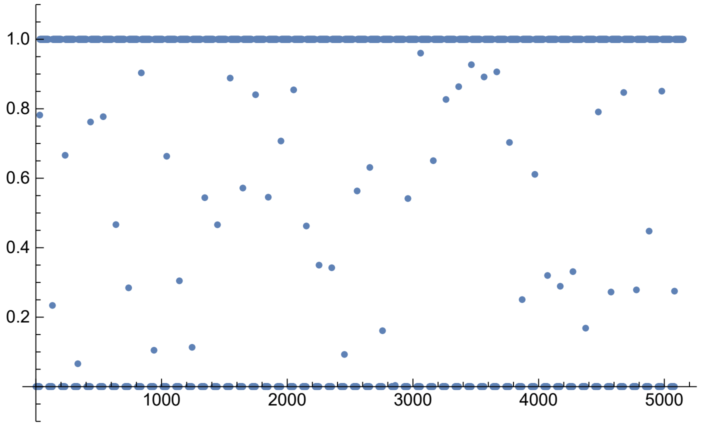

Out[◦]//InputForm=
{1.5*^-323, 1.}

```
(*Parameter values not changed*)
heatMap03 = ListDensityPlot[dataOrg2
,FrameLabel→{Style["",15,"DisplayFormula"],Style["",15,"DisplayFormula"]}
,ColorFunction→"BrownCyanTones"
,PlotRange→{0,1}
,ColorFunctionScaling -> False
,ImageSize→imagemSize
]
```

Out[ ]=

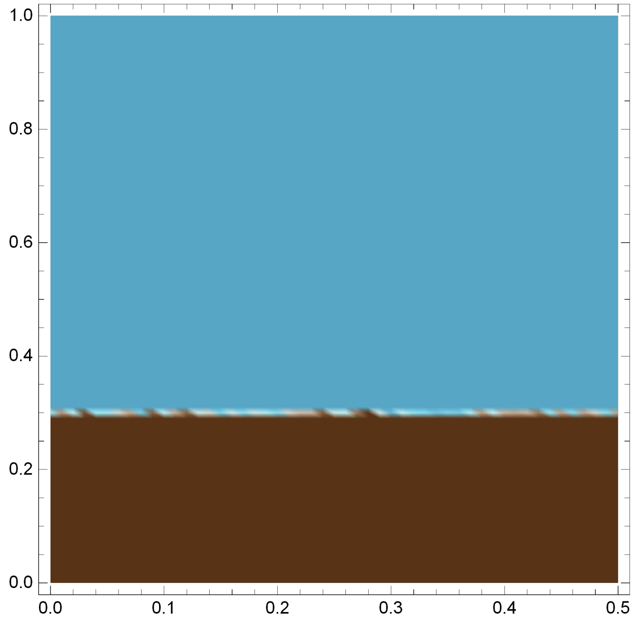

```
plotingLines=Plot[  {cG + x*(q*L/(q*L+q0)), cG + x*(q/(q+q0))},{x,0,1}  ,PlotRange→plotrange  ,
combinedPlots =Show[plotingLines, heatMap03 ,plotingLines];


a03=Graphics[
        {First[combinedPlots]
                    ,Inset[wt1      , wtlocation1    ,Automatic    ,Scaled[scale]]
                    ,Inset[wt2      , wtlocation2    ,Automatic    ,Scaled[scale]]
                    ,Inset[wt3      , wtlocation3    ,Automatic    ,Scaled[scale]]


                    (*,Inset[coexistienceLegend  ,{0.45,.56}    ,Automatic    ,Scaled[.17]]
                    ,Inset[coexistienceLegend  ,{0.45,.56}    ,Automatic    ,Scaled[.17]]
                    ,Inset[coexistienceLegend  ,{0.45,.56}    ,Automatic    ,Scaled[.17]]*)

                    ,Inset[Ag1    ,AgLocation1    ,Automatic    ,Scaled[scale]]
                    ,Inset[Ag2    ,AgLocation2    ,Automatic    ,Scaled[scale]]
                    ,Inset[Ag3    ,AgLocation3    ,Automatic    ,Scaled[scale]]
                    }
        ,PlotRange→plotrange
        ,AbsoluteOptions[combinedPlots]]
```

Out[ ]=

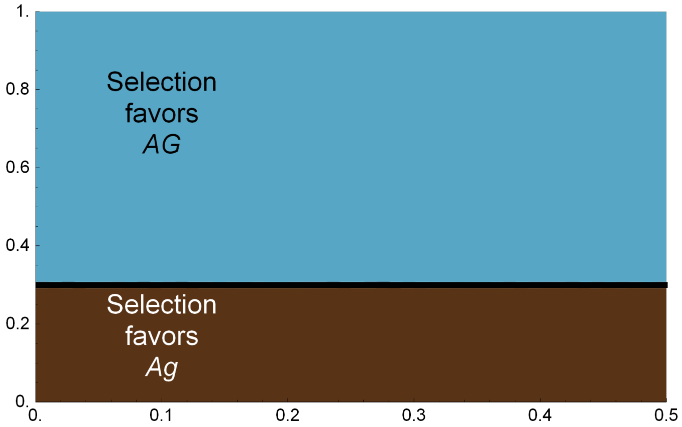

AG vs Ag, $q = 0.0$, $q_0 = 0.1$; graph a01

In[ ]:=

```
(*Parameter values not changed*)
q=0.0;  q0=0.1;
L=0.1;

cG=0.3;
```

```
SeedRandom[1234] (*setting the seed*)

(*Now calculating the time series *)
Tfinal=10000;
step=0.01;




f[L_,e_,q0_,q_,cA_,cG_,x_[t]]:= (x[t]*(1 - (cG + cA)*(q0 + q*(L + x[t] - L*x[t])) + (e + x[t]

dat=Table[{cA,e,RecurrenceTable[{x[t+1]==f[L,e,q0,q,cA,cG,x[t]],x[0]==RandomReal[]},x,{t,0,Tfinal
```

In[•]:=
```
dat // MatrixForm; (*the way that the data come out from Table[{c,e,RecurrenceTable[{x[t+1]==f[c,

parameters = Take[dat, All, All, 2]; (*only getting parameter values used to run the model:=getti
parameters// MatrixForm;

d=Dimensions[parameters];
d[[2]]; (*colecting the number of columns*)

dat[[;; , ;; , 3, -1]] // MatrixForm; (*only getting values of x[tFinal]*)
xFinal=Flatten[dat[[;; , ;; , 3, -1]]]; (*putting x[tFinal] into a single list*)
xFinal2=Partition[xFinal,1];(*first step in x[tFinal] partitioning*)
xFinal3=Partition[xFinal2,d[[2]]]; (*last step in x[tFinal] partitioning*)

dataOrg=Join[parameters,xFinal3,3];(*combining the parameters and x[tFinal] lists by at each 3 lo
dataOrg//MatrixForm;

dataOrg2=Flatten[dataOrg,1]; (*colapting the data in one list*)
dataOrg2//MatrixForm;

(*Values x[Tfinal]*)
Length[dataOrg2[[;;,3]]] (*Total number of x[Tfinal] points*)
Total[dataOrg2[[;;,3]]](*Sum of x[Tfinal],(PS.:x[t] is btw zero and 1 for all t*)
ListPlot[dataOrg2[[All,3]],PlotRange→{-0.1,1.1},PlotStyle -> PointSize[0.01]] (*Plotting all x[T
InputForm@MinMax[dataOrg2[[All,3]]]
```

Out[•]= 5151

Out[•]= 3596.78

Out[•]=
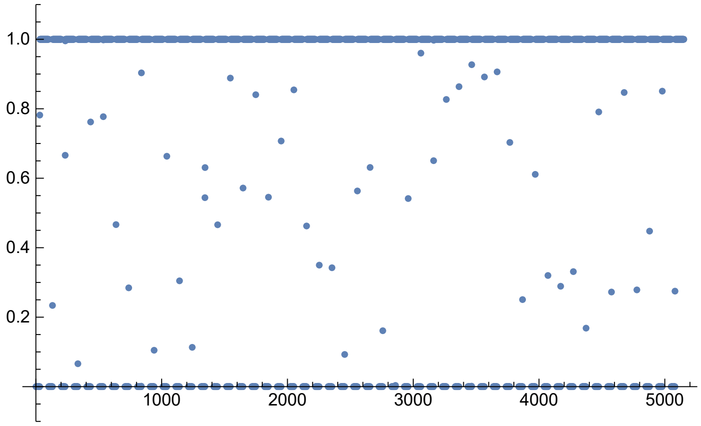

Out[•]//InputForm=
{4.836090905633788*^-140, 1.}

```
(*Parameter values not changed*)
heatMap01 = ListDensityPlot[dataOrg2
,FrameLabel→{Style["",15,"DisplayFormula"],Style["",15,"DisplayFormula"]}
,ColorFunction→"BrownCyanTones"
,PlotRange→{0,1}
,ColorFunctionScaling -> False
,ImageSize→imagemSize
]
```

Out[ ]=

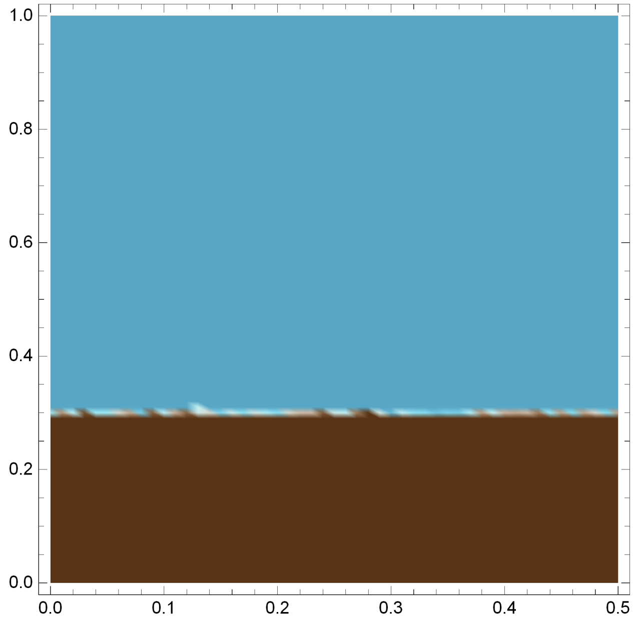

```
plotingLines=Plot[  {cG + x*(q*L/(q*L+q0)), cG + x*(q/(q+q0))},{x,0,1}  ,PlotRange→plotrange  ,
combinedPlots =Show[plotingLines, heatMap01 ,plotingLines];

a01=Graphics[
        {First[combinedPlots]
                    ,Inset[wt1      , wtlocation1    ,Automatic    ,Scaled[scale]]
                    ,Inset[wt2      , wtlocation2    ,Automatic    ,Scaled[scale]]
                    ,Inset[wt3      , wtlocation3    ,Automatic    ,Scaled[scale]]


                    (*,Inset[coexistienceLegend  ,{0.45,.56}    ,Automatic    ,Scaled[.17]]
                    ,Inset[coexistienceLegend  ,{0.45,.56}    ,Automatic    ,Scaled[.17]]
                    ,Inset[coexistienceLegend  ,{0.45,.56}    ,Automatic    ,Scaled[.17]]*)

                    ,Inset[Ag1    ,AgLocation1    ,Automatic    ,Scaled[scale]]
                    ,Inset[Ag2    ,AgLocation2    ,Automatic    ,Scaled[scale]]
                    ,Inset[Ag3    ,AgLocation3    ,Automatic    ,Scaled[scale]]
                    }
        ,PlotRange→plotrange
        ,AbsoluteOptions[combinedPlots]]
```

Out[∘]=

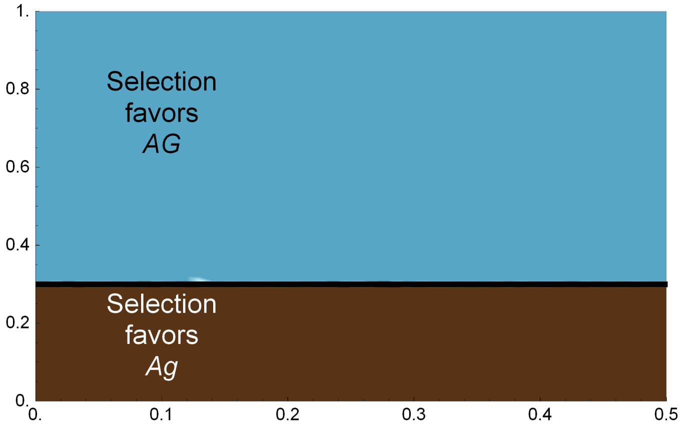

AG vs Ag, $q = 0.0$, $q_0 = 0.0$; graph a00

In[∘]:=

```
(*Parameter values not changed*)
q=0.0;  q0=0.0;
L=0.1;

cG=0.3;
```

```
SeedRandom[1234] (*setting the seed*)

(*Now calculating the time series *)
Tfinal=10000;
step=0.01;



f[L_,e_,q0_,q_,cA_,cG_,x_[t]]:= (x[t]*(1 - (cG + cA)*(q0 + q*(L + x[t] - L*x[t])) + (e + x[t]

dat=Table[{cA,e,RecurrenceTable[{x[t+1]==f[L,e,q0,q,cA,cG,x[t]],x[0]==RandomReal[]},x,{t,0,Tfinal
```

In[∘]:=

```
dat // MatrixForm; (*the way that the data come out from Table[{c,e,RecurrenceTable[{x[t+1]==f[c,


parameters = Take[dat, All, All, 2]; (*only getting parameter values used to run the model:=getti
parameters// MatrixForm;


d=Dimensions[parameters];
d[[2]]; (*colecting the number of columns*)

dat[[;; , ;; , 3, -1]] // MatrixForm; (*only getting values of x[tFinal]*)
xFinal=Flatten[dat[[;; , ;; , 3, -1]]]; (*putting x[tFinal] into a single list*)
xFinal2=Partition[xFinal,1];(*first step in x[tFinal] partitioning*)
xFinal3=Partition[xFinal2,d[[2]]]; (*last step in x[tFinal] partitioning*)

dataOrg=Join[parameters,xFinal3,3];(*combining the parameters and x[tFinal] lists by at each 3 lo
dataOrg//MatrixForm;

dataOrg2=Flatten[dataOrg,1]; (*colapting the data in one list*)
dataOrg2//MatrixForm;

(*Values x[Tfinal]*)
Length[dataOrg2[[;;,3]]] (*Total number of x[Tfinal] points*)
Total[dataOrg2[[;;,3]]](*Sum of x[Tfinal],(PS.:x[t] is btw zero and 1 for all t*)
ListPlot[dataOrg2[[All,3]],PlotRange→{-0.1,1.1},PlotStyle -> PointSize[0.01]] (*Plotting all x[T
InputForm@MinMax[dataOrg2[[All,3]]]
```

Out[∘]= 5151

Out[∘]= 2595.96

Out[∘]=

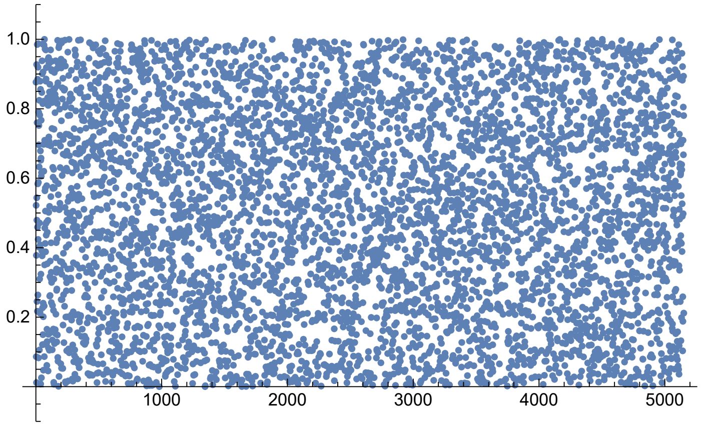

Out[∘]//InputForm=

{0.00005162655407953132, 0.9998965650751561}

```
heatMap00 = ListDensityPlot[dataOrg2
,FrameLabel→{Style["",15,"DisplayFormula"],Style["",15,"DisplayFormula"]}
,ColorFunction→"BrownCyanTones"
,PlotRange→{0,1}
,ColorFunctionScaling -> False
]
```

Out[ ]=

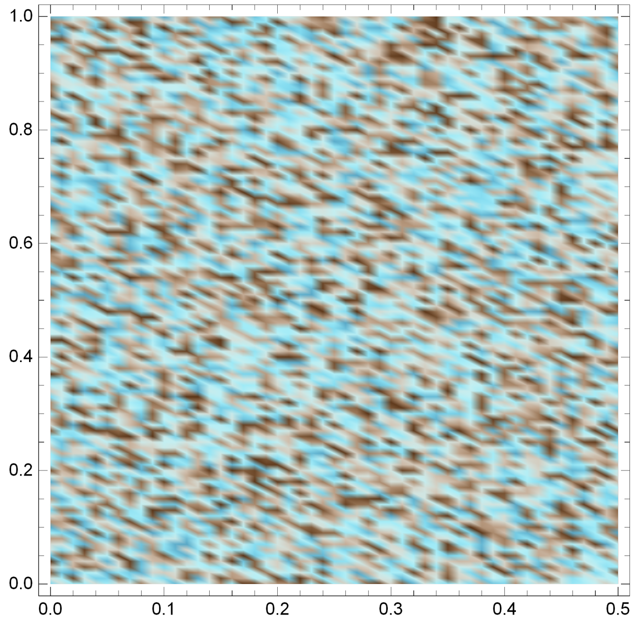

```
plotingLines=Plot[  {},{x,0,1}  ,PlotRange→plotrange  ,PlotStyle→{Black}  ,PlotTheme->{"DashedLi
combinedPlots =Show[plotingLines, heatMap00 ,plotingLines];


a00=Graphics[
        {First[combinedPlots]
                    (*,Inset[wt1      , wtlocation1    ,Automatic    ,Scaled[scale]]
                    ,Inset[wt2      , wtlocation2    ,Automatic    ,Scaled[scale]]
                    ,Inset[wt3      , wtlocation3    ,Automatic    ,Scaled[scale]]*)


                    (*,Inset[coexistienceLegend  ,{0.25,.56}    ,Automatic    ,Scaled[.17]]
                    ,Inset[coexistienceLegend  ,{0.25,.56}    ,Automatic    ,Scaled[.17]]
                    ,Inset[coexistienceLegend  ,{0.25,.56}    ,Automatic    ,Scaled[.17]]*)

                    (*,Inset[Ag1    ,AgLocation1    ,Automatic    ,Scaled[scale]]
                    ,Inset[Ag2    ,AgLocation2    ,Automatic    ,Scaled[scale]]
                    ,Inset[Ag3    ,AgLocation3    ,Automatic    ,Scaled[scale]]*)
                    }
        ,PlotRange→plotrange
        ,AbsoluteOptions[combinedPlots]]
```

Out[∘]=

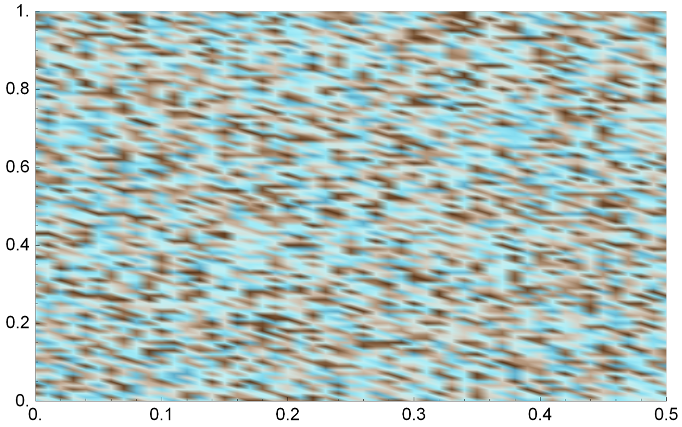

Combining individual figures

In[•]:=

```
barLegenda = BarLegend[{"BrownCyanTones",{0,1}},LegendLabel→Style["Fr. AG",14, Italic]]
```

Out[•]=

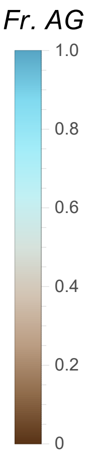

In[•]:=

```
co=Graphics[{Black,Dashed,Thickness[0.07],Line[{{1,1},{1.5,1}}]},ImageSize→{50,5},AspectRatio→Fu
li=Graphics[{Black,       Thickness[0.07],Line[{{1,1},{1.5,1}}]},ImageSize→{50,5},AspectRatio→Fu

lines=Grid[{
{co ,Style["C_A q/(q+q_0)",22,"DisplayFormula"]}
,{li ,Style["C_A qL/(qL+q_0)",22,"DisplayFormula"]}
}]
```

Out[•]=

$$C_A \frac{q}{q+q_0}$$

$$C_A \frac{\mathrm{qL}}{\mathrm{qL}+q_0}$$

In[•]:=

```
tabela=Grid[{{""  ,""                                        ,Style[" q_0 = 0.5",22,"Display
,{"", ""                                  ,a55                   ,a51                    ,a50
,{"", Rotate[Style["e - C_G",22,"DisplayFormula"],90 Degree] ,a35                   ,a31
,{""  , ""                                  ,a05                   ,a01                    ,a00
,{""                    , ""                             ,"" , Style[" Autoinducer's cost, C_A '
,Dividers→{{7→{Gray,Thick}},{2→{Gray,Thick}}}]
```

Out[ ]=

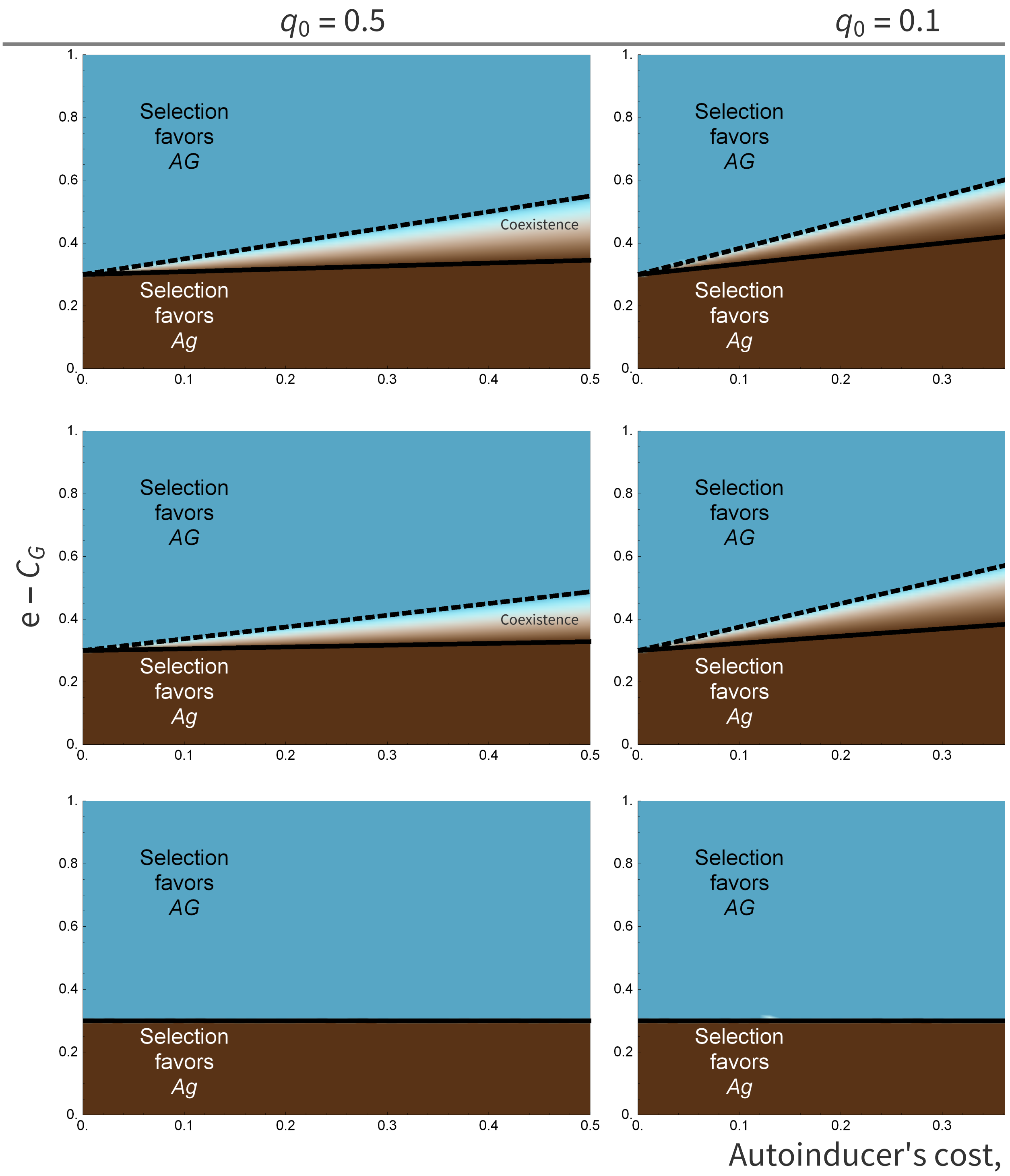

In[ ]:=

```
Export["C:\\Users\\Lucas Santana Souza\\Pictures\\exportmathematica image\\tabelaAG_aG_Heatmap_An
```

Out[ ]= C:\Users\Lucas Santana Souza\Pictures\exportmathematica image\tabelaAG_aG_Heatmap_AnnotationWithLines.png
